# Supplementary material for: Clinical features and natural history of the first 2073 suspected COVID-19 cases in the Corona São Caetano primary care programme: a prospective cohort study
Source: BMJ Open. 2021 Jan 12;11(1):e042745. doi: 10.1136/bmjopen-2020-042745 (PMC7805372; doi:10.1136/bmjopen-2020-042745)
Supplement: Supplementary data [file bmjopen-2020-042745supp001.pdf]

Supplemental material

Contents:

1. **Initial risk assessment**
2. **Screen shots of multimedia platform and the initial questionnaire**
3. **Supplemental Table S1**
4. **Supplemental figs S1-S5**

Initial risk assessment

Patients meeting the definition of a suspected case were called by a medical student (under supervision) to complete a risk assessment. All patients were asked a set of standardized questions:

- Do you feel short of breath?
- Are you breathing quickly or finding it difficult to breath?
- If yes, can you count your respiratory rate over one minute? (respiratory rate >20 breaths/minute was considered tachypnoea)
- Has your fever worsened over the last 3 days or have you had a new fever after 2 days being fever-free?
- Have you felt confused or lethargic?

If the patient answered "yes" to any of these questions they were advised to attend a specialist health service. Among the 132 patients that were triaged to hospital, 76 (58% of 132) had shortness of breath, 76 (58% of 132) reported rapid breathing, 33 (25% of 132) persistent fever and 22 (17% of 132) altered mental status.

Screen shots showing examples of the initial questionnaire completed

1) Welcome page

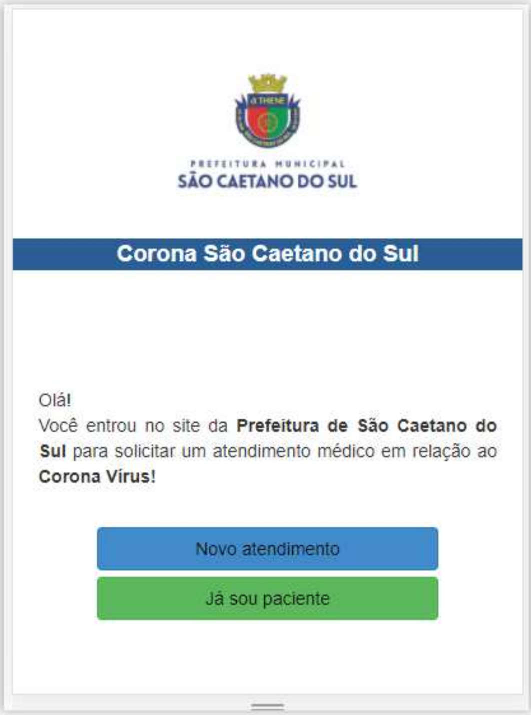

2) Zipcode confirmation

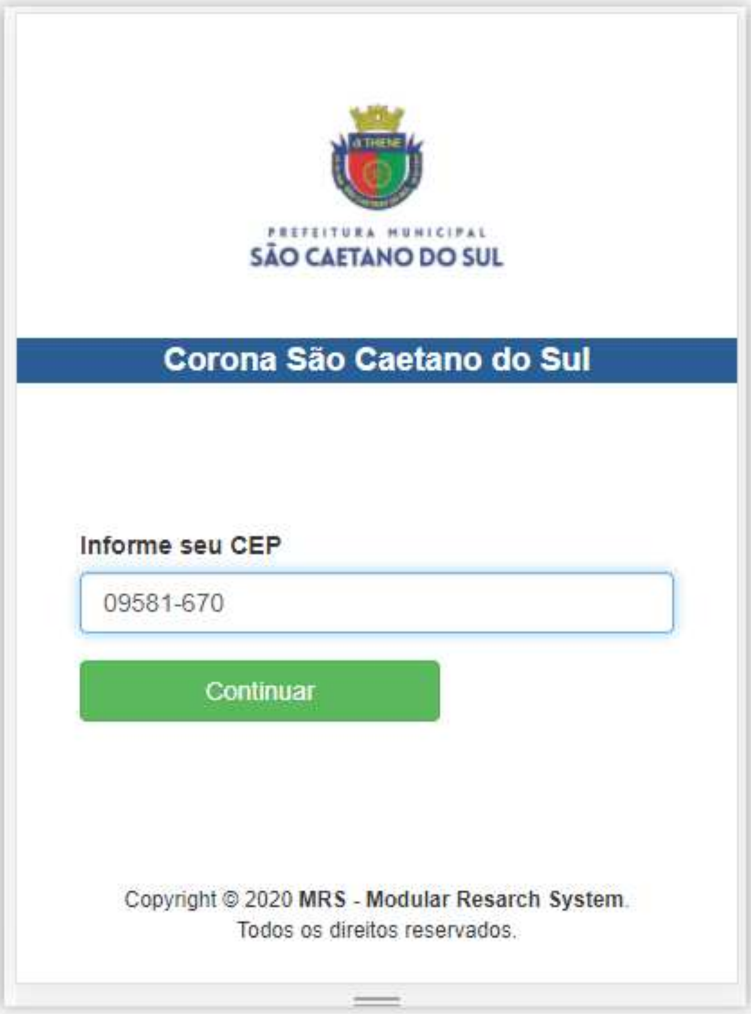

3) Patient basic information

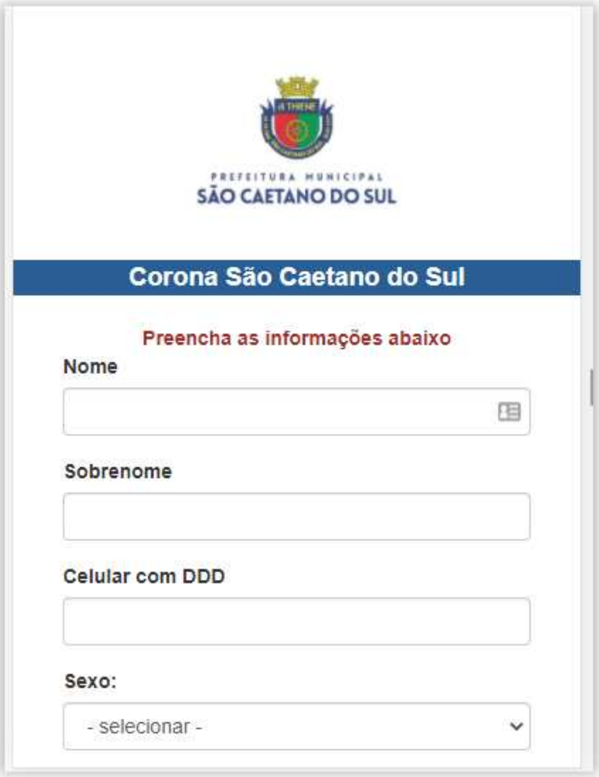

4) Access code confirmation

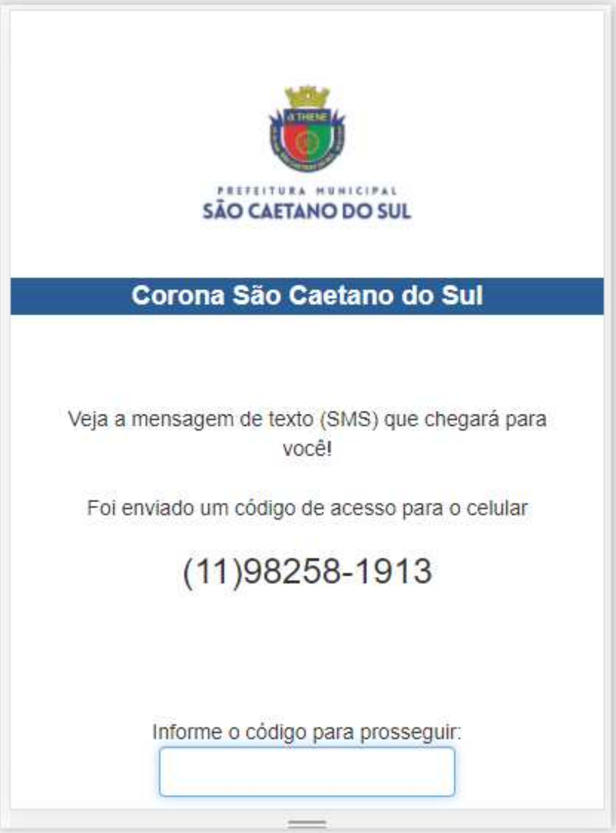

5) Questionnaire

Perguntas obrigatórias, favor responder:

Dados demográficos

Se do sexo feminino, está grávida?

- Não
- Sim
- Não se aplica

A partir do dia 1º de Março você atuou em alguma destas áreas:

- Não
- Profissional da saúde
- Áreas essenciais (segurança, bombeiro, farmácia, supermercado, transporte público)
- Cuidador (a)

Dados clínicos

Teve febre?

- Não
- Sim

Se sim, você mediu a febre?

- Não
- Sim

Se sim, qual foi a temperatura mais alta?

Você tem tosse?

- Não
- Sim

6) Orientation page

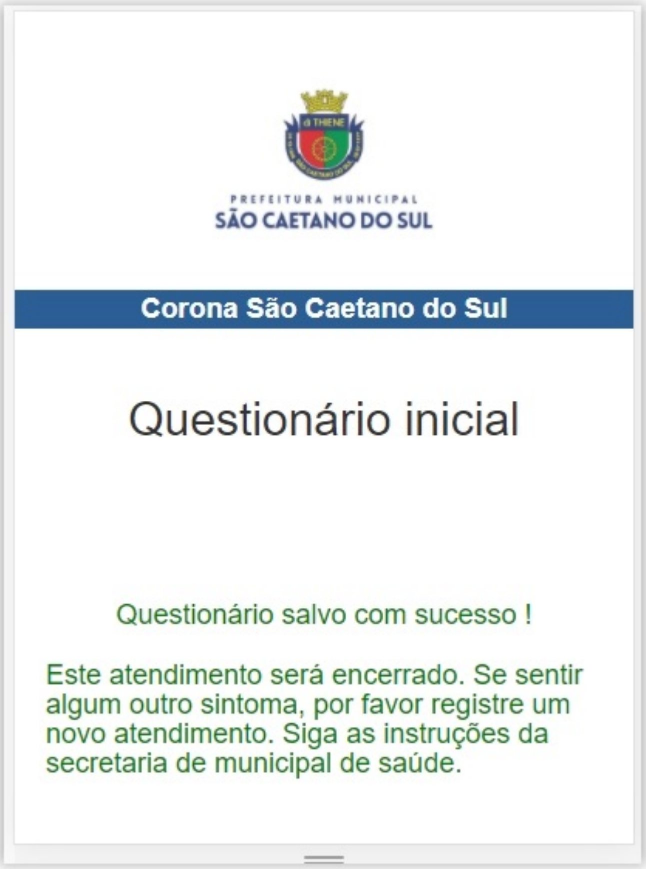

Table S1 Univariable and adjusted associations between RT-PCR cycle thresholds and patient characteristics

	**Unadjusted analysis**		**Adjusted analysis ***	
	**Beta** (difference in means)	**95% Confidence interval**	**Beta** (difference in means)	**95% Confidence interval**
**Age** (years)	**-0.05**	**-0.09 to -0.01**	**-0.06**	**-0.09 to -0.03**
**Male sex**	**-1.36**	**-2.49 to -0.23**	-1.05	-2.09 to <0.001
**Days from symptom onset to swab collection** (days, log_2)	**3.28**	**2.33 to 4.03**	**3.27**	**0.42 to 7.70**
**PCR platform** (ALTONA as reference)	**-1.19**	**-2.37 to -0.02**	**-1.53**	**-2.6 to -0.45**
**Symptoms at presentation**				
Fever	**-1.78**	**-2.96 to -0.59**	**-1.11**	**-2.11 to -0.001**
Myalgia	-1.31	-2.75 to 0.12	-0.78	-2.11 to 0.53
Arthralgia	**-1.64**	**-2.77 to -0.52**	**-1.24**	**-2.18 to -0.10**
Anosmia	**3.15**	**2.04 to 4.25**	**2.21**	**1.0 to 3.29**
Agusia	**2.99**	**1.89 to 4.09**	**1.96**	**0.88 to 3.0**
Diarrhea	**2.19**	**0.84 to 3.53**	**1.36**	**0.12 to 2.61**
Nausea	**1.50**	**0.28 to 2.72**	1.09	-0.04 to 2.24
Vomiting	**2.99**	**0.52 to 5.46**	2.02	-0.28 to 4.33
Anorexia	0.56	-0.57 to 1.70	0.47	-0.58 to 1.51
Headache	-0.58	-2.12 to 0.97	-0.81	-2.25 to 0.63
Fatigue	0.84	-0.50 to 2.18	0.34	-0.91 to 1.59
Coryza	-0.78	-1.92 to 0.34	-0.68	-1.72 to 0.34
Blocked nose	-0.36	-1.53 to 0.81	**-1.48**	**-2.59 to -0.37**
Cough	-1.33	-2.70 to 0.03	**-1.60**	**-2.86 to -0.33**
Sore throat	-0.49	-1.62 to 0.64	-0.45	-1.52 to 0.61

* All variables adjusted for age (continuous in years), sex (female as reference group), PCR platform (ALTONA platform as the reference group) and time between symptom onset and swab collection (log base 2). Analysis was performed within a linear regression framework. Positive beta coefficients indicate higher cycle thresholds (lower viral load) associated with that variable, whereas negative beta coefficients indicate lower cycle thresholds when the variable is present. Results in bold reached statistical significance.

Supplemental figures

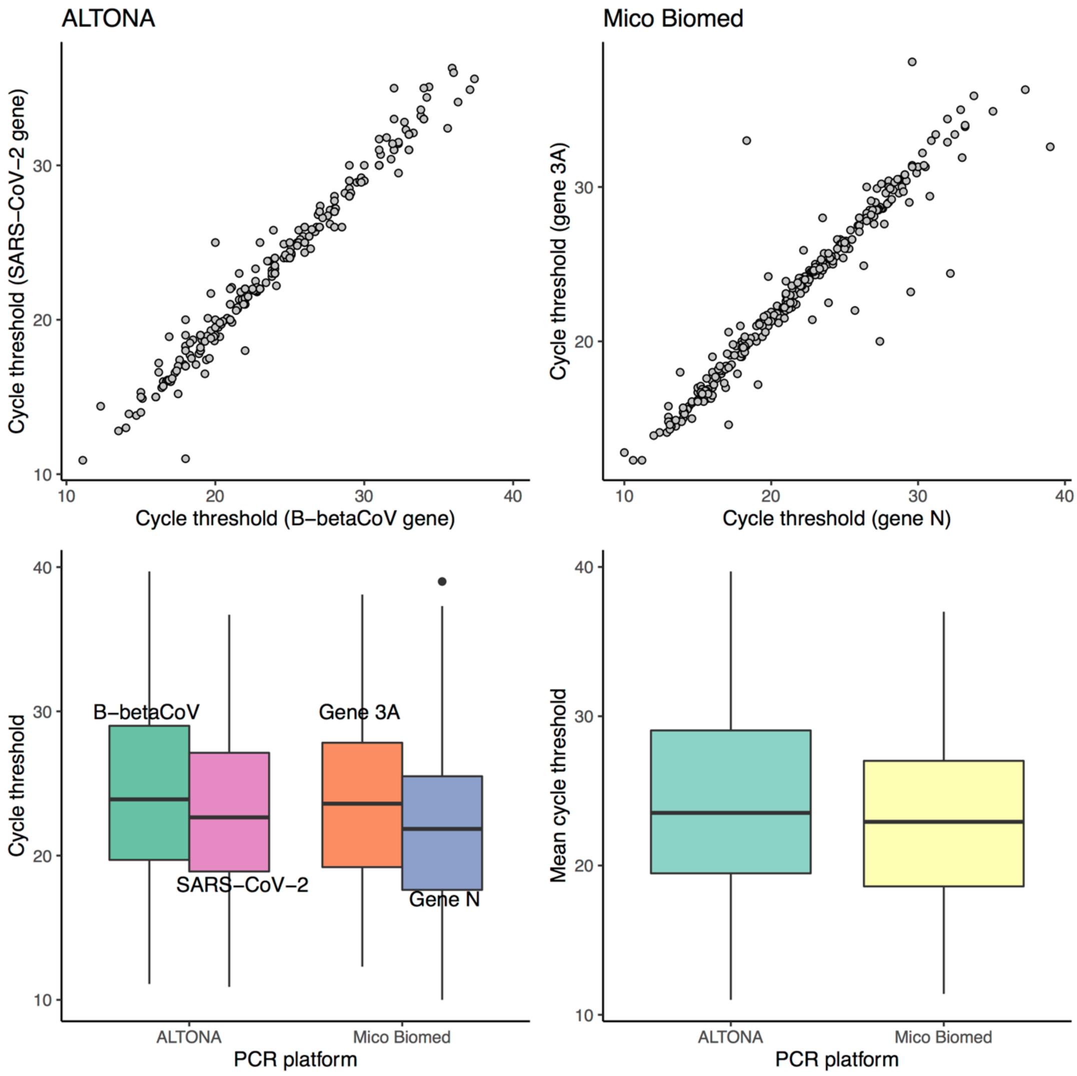

Figure S1 Comparison of cycle thresholds across PCR platforms and genes amplified. Upper two panels show the concordance between cycle thresholds for the two separate genes amplified by the ALTONA (left) and Mico Biomed (right) kits. Lower left panel – distribution of cycle thresholds by gene amplified and RT-PCR platform used. Lower right-hand panel – distribution of the mean cycle threshold (mean of cycle thresholds for separate genes) between different RT-PCR platforms.

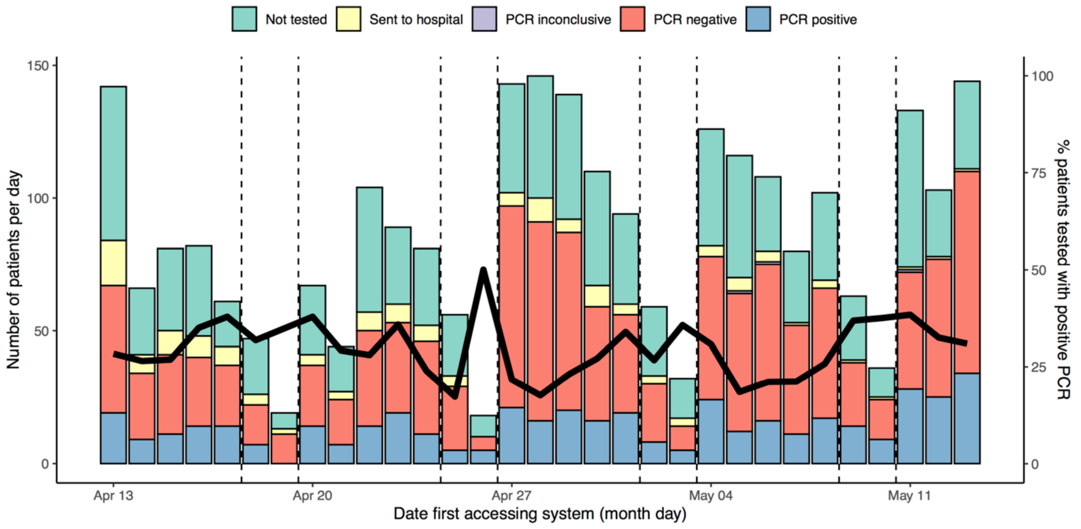

Figure S2 Time series of presentations to the Corona São Caetano platform. Dashed vertical lines denote the weekends with a reduced number of presentations. Thick black line corresponds to the right-hand y-axis: proportion of RT-PCRs performed with positive result.

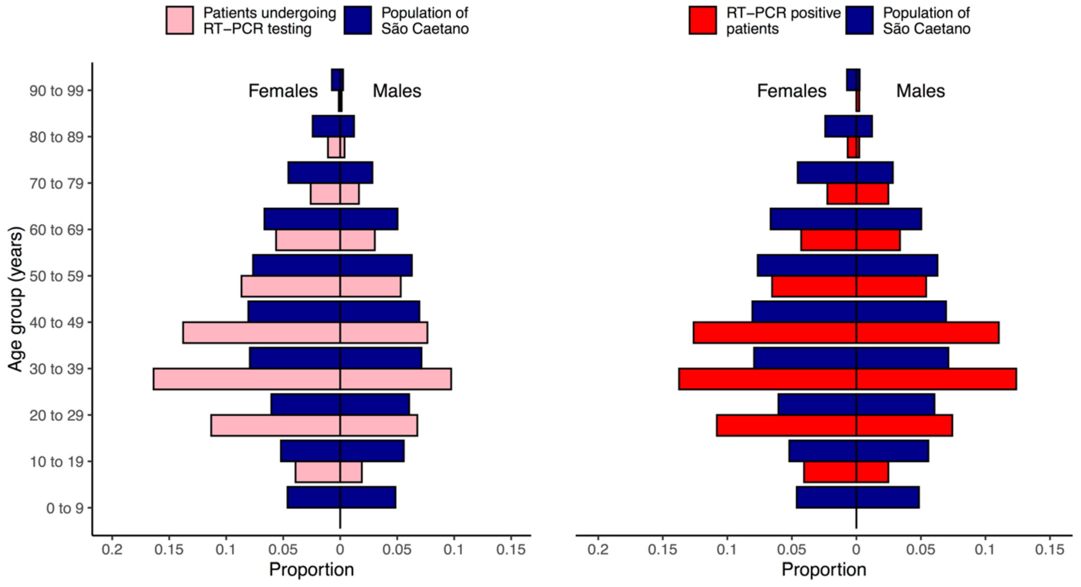

Figure S3 Age-sex distribution the city of São Caetano do Sul compared with that of patients accessing the Corona São Caetano system and being tested with RT-PCR (left-hand panel) and those testing positive for SARS-CoV-2 (right-hand panel).

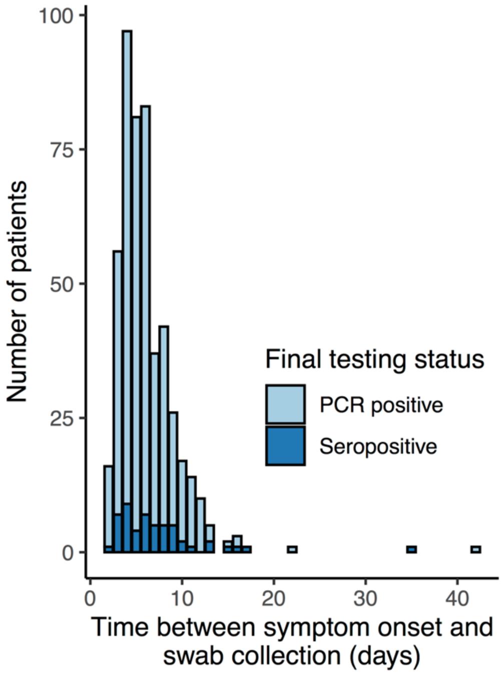

Figure S4 Histogram of delay between symptom onset and swab collection among patients with COVID-19.

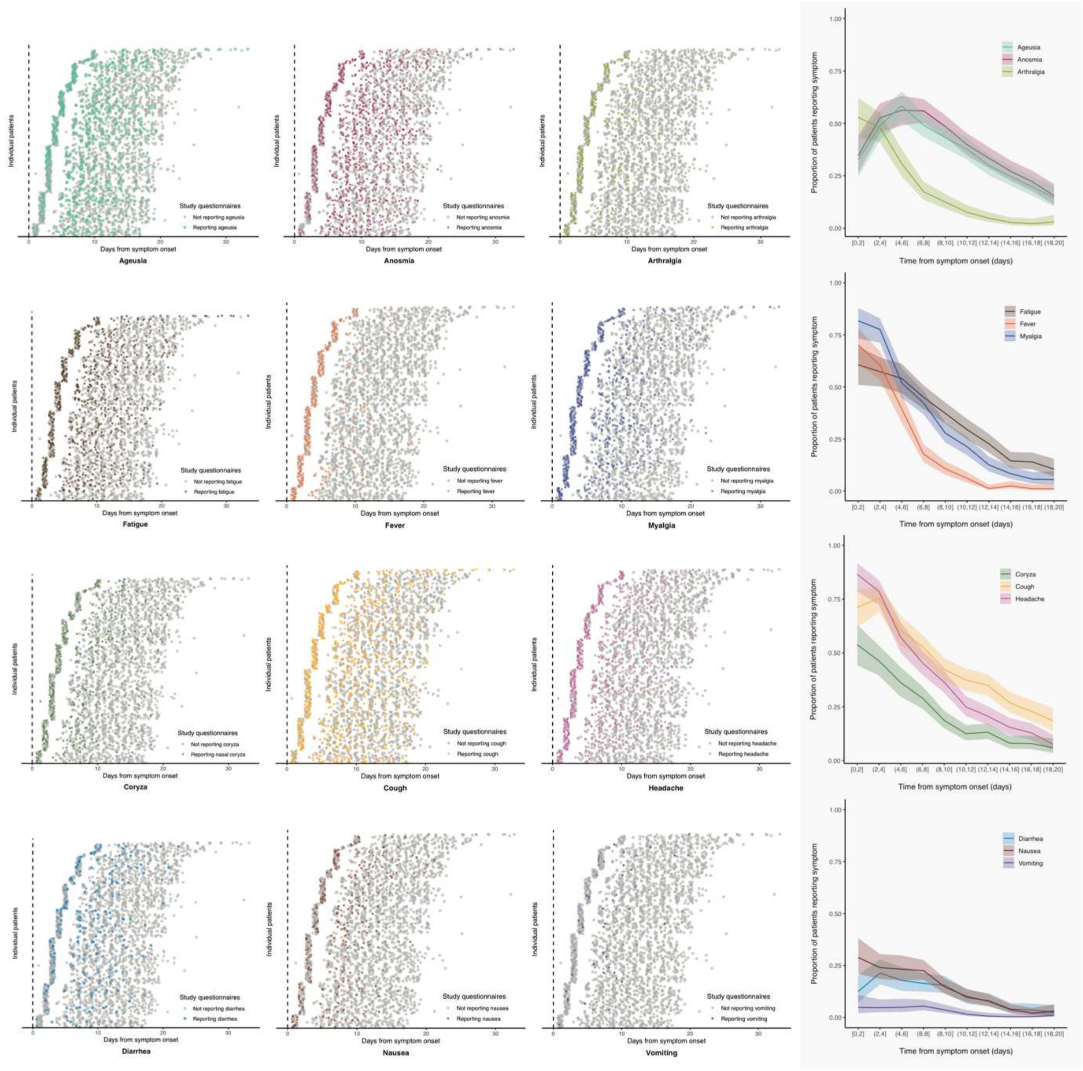

Figure S5 Left hand figures show symptoms at each follow-up questionnaire among patients testing RT-PCR positive and undergoing follow-up. Individual patients are stacked on the y-axis ordered according to the delay from symptom onset to presentation. Each point represents the response to a questionnaire and its position on the horizontal axis the time after symptom onset that the questionnaire was filled in. Grey points are questionnaires where the patient denied the presence of a given symptom. The coloured points correspond to questionnaires in which the patient reported a given symptom. The right-hand figures results from grouping the horizontal axis time into two-day windows and calculating the proportion of completed questionnaires in which each symptom was reported. The denominators for the horizontal axis groups (number of questionnaires completed within a given time window from symptom onset) are 104 at [0-2] days, 192 at (2-4], 185 at (4-6], 293 at (6-8], 338 at (8-10], 329 at (10-12], 335 at (12-14], 324 at (14-16], 280 at (16-18] and 201 at (18-20].
