# Supplementary material for: Clinical features and natural history of the first 2073 suspected COVID-19 cases in the Corona São Caetano primary care programme: a prospective cohort study
Source: BMJ Open. 2021 Jan 12;11(1):e042745. doi: 10.1136/bmjopen-2020-042745 (PMC7805372; doi:10.1136/bmjopen-2020-042745)
Supplement: Author's manuscript [file bmjopen-2020-042745.draft_revisions.pdf]

BMJ Open

BMJ Open is committed to open peer review. As part of this commitment we make the peer review history of every article we publish publicly available.

When an article is published we post the peer reviewers' comments and the authors' responses online. We also post the versions of the paper that were used during peer review. These are the versions that the peer review comments apply to.

The versions of the paper that follow are the versions that were submitted during the peer review process. They are not the versions of record or the final published versions. They should not be cited or distributed as the published version of this manuscript.

BMJ Open is an open access journal and the full, final, typeset and author-corrected version of record of the manuscript is available on our site with no access controls, subscription charges or pay-per-view fees (http://bmjopen.bmj.com).

If you have any questions on BMJ Open's open peer review process please email info.bmjopen@bmj.com

BMJ Open

A primary care approach to the COVID-19 pandemic: clinical features and natural history of 2,073 suspected cases in the Corona Sao Caetano programme, Sao Paulo, Brazil

Journal:	*BMJ Open*
Manuscript ID	bmjopen-2020-042745
Article Type:	Original research
Date Submitted by the Author:	14-Jul-2020

Complete List of Authors:	Leal, Fabio ; Universidade Municipal de São Caetano do Sul; Instituto Nacional de Cancer<br>Mendes-Correa, Maria; Universidade de São Paulo, Instituto de Medicina Tropical (LIM-52, LIM-46, LIM-49) and Departamento de Moléstias Infecciosas e Parasitarias<br>Buss, Lewis; Universidade de São Paulo, Instituto de Medicina Tropical (LIM-52, LIM-46, LIM-49) and Departamento de Moléstias Infecciosas e Parasitarias<br>Costa, Silvia; Universidade de São Paulo, Instituto de Medicina Tropical (LIM-52, LIM-46, LIM-49) and Departamento de Moléstias Infecciosas e Parasitarias<br>Bizario, Joao; Universidade Municipal de São Caetano do Sul<br>de Souza, Sonia ; Universidade Municipal de São Caetano do Sul<br>Thomaz, Osorio; Instituto de Pesquisas Tecnológicas<br>Tozetto-Mendoza, Tania; Universidade de São Paulo, Instituto de Medicina Tropical (LIM-52, LIM-46, LIM-49) and Departamento de Moléstias Infecciosas e Parasitarias<br>Villas-Boas, Lucy; Universidade de São Paulo, Instituto de Medicina Tropical (LIM-52, LIM-46, LIM-49) and Departamento de Moléstias Infecciosas e Parasitarias<br>Oliveira, Lea; Universidade de São Paulo, Instituto de Medicina Tropical (LIM-52, LIM-46, LIM-49) and Departamento de Moléstias Infecciosas e Parasitarias<br>Grespan, Regina; Universidade Municipal de São Caetano do Sul<br>Capuani, Ligia; Modular Research System<br>Buccheri, Renata; Universidade de São Paulo, Instituto de Medicina Tropical (LIM-52, LIM-46, LIM-49) and Departamento de Moléstias Infecciosas e Parasitarias<br>Domingues, Helves; Modular Research System<br>Alexander, Neal; London School of Hygiene & Tropical Medicine<br>Mayaud, Philippe; London School of Hygiene & Tropical Medicine<br>Sabino, Ester; Universidade de São Paulo, Instituto de Medicina Tropical (LIM-52, LIM-46, LIM-49) and Departamento de Moléstias Infecciosas e Parasitarias

Keywords:	PRIMARY CARE, Epidemiology < TROPICAL MEDICINE, INFECTIOUS DISEASES, PUBLIC HEALTH

For peer review only - http://bmjopen.bmj.com/site/about/guidelines.xhtml

I, the Submitting Author has the right to grant and does grant on behalf of all authors of the Work (as defined in the below author licence), an exclusive licence and/or a non-exclusive licence for contributions from authors who are: i) UK Crown employees; ii) where BMJ has agreed a CC-BY licence shall apply, and/or iii) in accordance with the terms applicable for US Federal Government officers or employees acting as part of their official duties; on a worldwide, perpetual, irrevocable, royalty-free basis to BMJ Publishing Group Ltd ("BMJ") its licensees and where the relevant Journal is co-owned by BMJ to the co-owners of the Journal, to publish the Work in this journal and any other BMJ products and to exploit all rights, as set out in our licence.

The Submitting Author accepts and understands that any supply made under these terms is made by BMJ to the Submitting Author unless you are acting as an employee on behalf of your employer or a postgraduate student of an affiliated institution which is paying any applicable article publishing charge ("APC") for Open Access articles. Where the Submitting Author wishes to make the Work available on an Open Access basis (and intends to pay the relevant APC), the terms of reuse of such Open Access shall be governed by a Creative Commons licence – details of these licences and which Creative Commons licence will apply to this Work are set out in our licence referred to above.

Other than as permitted in any relevant BMJ Author's Self Archiving Policies, I confirm this Work has not been accepted for publication elsewhere, is not being considered for publication elsewhere and does not duplicate material already published. I confirm all authors consent to publication of this Work and authorise the granting of this licence.

For peer review only

A primary care approach to the COVID-19 pandemic: clinical features and natural history of 2,073 suspected cases in the Corona São Caetano programme, São Paulo, Brazil

Fabio E Leal[1,2]* (PhD), Maria C Mendes-Correa[3]* (PhD), Lewis F Buss[3]*# (MBBS), Silvia F Costa[3] (PhD), Joao CS Bizario[1] (PhD), Sonia RP de Souza[1] (PhD), Osorio Thomaz[4] (PhD), Tania R Tozetto-Mendoza[1] (PhD), Lucy S Villas-Boas[3], Lea CO Silva[3] (PhD), Regina MZ Grespan (MD)[1], Ligia Capuani[5] (PhD), Renata Buccheri[3] (MD), Helves Domingues (PhD)[5], Neal DE Alexander[6] (PhD), Philippe Mayaud[6] (MD), Ester C Sabino[3] (PhD)

* These authors contributed equally

Affiliations

1. Universidade Municipal de São Caetano do Sul (USCS), Rua Santo Antonio, São Caetano do Sul, Brazil
2. National Cancer Institute (INCA), Praça Cruz Vermelha, Rio de Janeiro, Brazil
3. Instituto de Medicina Tropical (LIM-52, LIM-46, LIM-49) and Departamento de Moléstias Infecciosas e Parasitarias da Faculdade de Medicina da Universidade de São Paulo Avenida Dr. Enéas Carvalho de Aguiar, São Paulo, Brazil
4. Instituto de Pesquisas Tecnológicas do Estado de São Paulo - IPT
5. Modular Research System, Avenida Paulista, São Paulo, Brazil
6. London School of Hygiene and Tropical Medicine (LSHTM), Keppel Street, London, United Kingdom

Corresponding author:

Dr Lewis F Buss

Instituto de Medicina Tropical da Faculdade de Medicina da Universidade de São Paulo

lewisbuss@usp.br

KEY WORDS: SARS-CoV-2, COVID-19, pandemic, community, primary care, Brazil

For peer review only

ABSTRACT

Background: Despite most cases not requiring hospital care, there are limited community-based clinical data on COVID-19.

Methods: The Corona São Caetano program is a primary care initiative offering COVID-19 care to all residents of São Caetano do Sul, Brazil. After triage of potentially severe cases, consecutive patients presenting between 13th April and 13th May 2020 were tested at home with SARS-CoV-2 reverse transcriptase (RT) PCR; positive patients were followed up for 14 days. RT-PCR-negative patients were offered SARS-CoV-2 serology. We describe the clinical features, virology and natural history of this prospective population-based cohort.

Findings: Of 2,073 suspected COVID-19 cases, 1,583 (76.4%) were tested by RT-PCR, of whom 444 (28.0%, 95%CI: 25.9% - 30.3%) were positive; 604/1,136 (53%) RT-PCR-negative patients underwent serology, of whom 52 (8.6%) tested SARS-CoV-2 seropositive. The most common symptoms of COVID-19 were cough, fatigue, myalgia and headache; whereas self-reported fever (OR 3.0, 95%CI 2.4-3.9), anosmia (OR 3.3, 95%CI 2.6-4.4), and ageusia (2.9, 95%CI 2.3-3.8) were most associated with a positive COVID-19 diagnosis. RT-PCR cycle thresholds were lower in men, older patients, those with fever and arthralgia, and around symptom onset. The rates of hospitalization and death among 444 RT-PCR-positive cases were 6.7% and 0.7%, respectively, with older age and obesity more frequent in the hospitalized group.

Conclusion: COVID-19 presents in a similar way to other mild respiratory disease in the community, but some symptoms can assist the differential diagnosis. Most patients recovered without requiring hospitalization with a low fatality rate compared to hospital-based studies.

For peer review only

Strengths and limitations of this study

1. The clinical features of COVID-19 have mostly been described in hospital-based studies which are necessarily biased towards severe disease
2. We report a prospective cohort of suspected and confirmed COVID-19 cases from a primary care initiative in the Brazilian municipality of São Caetano do Sul
3. By systematically testing consecutive suspected community cases with molecular and serological tests we were able to address the diagnostic value of clinical features of mild-moderate COVID-19 in primary care
4. Prospective follow-up of confirmed cases and linkage with hospital datasets allowed us to describe the natural history of a primary care COVID-19 population
5. A limitation of the work was that not all participants underwent serology testing due to loss to follow-up

For peer review only

For peer review only - http://bmjopen.bmj.com/site/about/guidelines.xhtml

INTRODUCTION

A comprehensive public health response is vital but difficult to achieve during an epidemic. The COVID-19 pandemic, caused by the novel severe acute respiratory syndrome coronavirus 2 (SARS-CoV-2), started in China in late 2019.[1] According to the World Health Organization (WHO)[2] and others[3], the ideal early response should have been multipronged, with identification, isolation, treatment and contact tracing of symptomatic cases, relying on a strong testing programme. Primary health care (PHC) is well placed to implement such a response, by identifying cases early and managing them in a way that minimizes overcrowding of emergency rooms and intensive care units.[4] Real-time data analysis coming from these primary care response systems can inform policy decisions.

In Brazil, the first case of COVID-19 was identified in the city of São Paulo on 26th February 2020.[5] As of 15th June 2020 there were 1,400,000 cases nationally with São Paulo contributing a fifth of these.[6] In March 2020, the Municipal Health Department of the municipality of São Caetano do Sul – part of the Greater Metropolitan Region of São Paulo – began to develop a clinical and testing platform to organize its COVID-19 response. The aim was to provide universal detection and management of symptomatic cases and their contacts. The platform was developed in partnership with two local universities – the Municipal University of São Caetano do Sul (USCS) and the University of Sao Paulo (USP) – and called "Corona São Caetano".

Large scale community-based observational cohorts are difficult to establish under epidemic circumstances, particularly if the risk of exposure for research personnel is high. Hence, most COVID-19 epidemiological and clinical studies have been hospital-based,[7–9] and therefore tend to include more severe cases whose findings may not be generalizable to the general population.[10] The objectives of this study were to describe the epidemiological indicators of the early phase of the programme rollout; and to describe the clinical, virologic and natural history features (including hospitalization and deaths) of SARS-CoV-2 infection among patients identified in primary care.

For peer review only - http://bmjopen.bmj.com/site/about/guidelines.xhtml

METHODS

Setting

The municipality of São Caetano do Sul has a population of 161,000 inhabitants.[11] The active aging index (i.e., the ratio of population aged >60 yr / population aged ≤14 yr) is 135, compared to the Brazilian average of 52, reflecting an aging population;[11] its Human Development Index is one of the highest in the country; nearly all (97.4%) children aged 6-14 are in education and 31% of the population have completed higher education[12] (Brazilian national average is 11%).

Corona São Caetano platform

Residents of the municipality aged 12 years and older with suspected COVID-19 symptoms were encouraged to contact the dedicated Corona São Caetano platform via the website (access at https://coronasaocaetano.org/) or by phone. They were invited to complete an initial screening questionnaire that included socio-demographic data; information on symptoms type, onset and duration; and recent contacts.

Patients meeting the suspected COVID-19 case definition (i.e., having at least two of the following symptoms: fever, cough, sore throat, coryza, or change in/loss of smell (anosmia); or one of these symptoms plus at least two other symptoms consistent with COVID-19) were further evaluated, whilst people not meeting these criteria were reassured, advised to stay at home and contact the service again if they were to develop new symptoms or worsening of current ones. Patients were then called by a medical student to complete a risk assessment. All pregnant women, and patients meeting pre-defined triage criteria for severe disease (see Supplemental Material), were advised to attend a hospital service - either an emergency department or outpatient service, depending on availability. All other patients were offered a home visit for self-collection of a nasopharyngeal swab.

Sample collection

Nasopharyngeal swabs (NPS – both nostrils and throat) were collected at the patients' homes under the supervision of trained healthcare personnel. A link to a video

For peer review only

(https://youtu.be/rWZzV2ZP7KY) was sent to the patients, before the home visit, to provide guidance on self-collection procedures. Healthcare personnel were instructed to maintain a distance of six feet from the patient and to wear personal protective equipment at all times. Samples were immediately put on a cool box between 2-8°C and stored at 4°C in a fridge until shipment to the lab within 24 hours.

Follow-up procedures

Patients testing SARS-CoV-2 RT-PCR positive were followed up to 14 days (a maximum of 7 phone calls) from completion of their initial questionnaire. They were contacted every 48 hours by a medical student who completed another risk assessment and recorded any ongoing or new symptoms. Patients testing RT-PCR negative were followed up by the primary health care program for their residential area. They were advised to contact the platform for a new consultation if they developed new symptoms. Starting on May 19th, when serological testing became available, RT-PCR-negative patients were re-contacted to offer antibody (IgG/IgM combined) testing 14 days after their initial registration as long as they had become asymptomatic.

Study dates

The Corona São Caetano programme was launched on 6th April 2020 and is still ongoing at the time of writing. For this analysis, we opted to include all patients making their first contact with the programme between 13th April and 13th May 2020. This comprises the first 31 days of the response, having excluded the first week, which corresponded to a pilot phase designed to test instruments before roll-out. The period of follow-up (last date of data extraction) was 4th June 2020, to account for the accrual period (three weeks) of possible hospitalizations in the last included patients.

Laboratory methods

Due to shortages of some reagents, two RT-PCR platforms were used at different times during the study: ALTONA RealStar® SARS-CoV-2 RT-PCR Kit 1.0 (Hamburg, Germany) and the Mico BioMed RT-qPCR kit (Seongnam, South Korea). For serology we tested 10µL of serum or plasma (equivalent in performance) using a qualitative rapid chromatographic

For peer review only - http://bmjopen.bmj.com/site/about/guidelines.xhtml

immunoassay (Wondfo Biotech Co., Guangzhou, China), that jointly detects anti-SARS-CoV-2 IgG/IgM. The assay has been found to have a sensitivity of 81.5% and specificity of 99.1% in a US study[13]. In our local validation, after two weeks of symptoms, the sensitivity in 59 RT-PCR confirmed cases was 94.9%, and specificity in 106 biobank samples from 2019 was 100%.

Statistical methods

We estimated the contribution of our platform to COVID-19 diagnosis in São Caetano do Sul. We compared the number of cases diagnosed in our programme with official data released by the Municipal Department of Health in its daily bulletins (accessed here https://coronavirus.saocaetanodosul.sp.gov.br).

Clinical and demographic data were extracted directly from the Corona São Caetano information system, with the last export on 5th June, to allow for follow-up of patients at the end of the study period. To analyse clinical presentation, we first calculated the proportion and exact binomial 95% confidence intervals (CI) of cases reporting each symptom in the three testing groups: SARS-CoV-2 RT-PCR positive; RT-PCR negative / seropositive; and RT-PCR negative / seronegative. We next combined RT-PCR and serology positive cases to make confirmed COVID-19 group, and those negative on both tests to make a SARS-CoV-2 negative control group. We express the association between each symptom and a positive COVID-19 diagnosis as odds ratios (OR) and 95% CIs.

Next, we assessed associations between RT-PCR cycle thresholds (Cts) and other clinical features. ALTONA and MiCo BioMed RT-PCR kits each separately amplify two different SARS-CoV-2 viral genes, as such each patient had two Ct values. There was a high concordance between Cts for the two genes within each kit (Figure S1), and we opted therefore to use the mean of the two Ct values for each patient in all analyses. We calculated univariable associations between Cts and age, sex, delay from symptom onset to NPS collection, and presenting symptoms using simple linear regression. We then built a multivariable linear regression model to assess independent associations between presenting symptoms and RT-PCR Cts. As age, sex, and time of swab collection may confound this relationship we included these variables, as well as the RT-PCR platform (ALTONA vs MiCo BioMed), as covariates in the model.

For peer review only - http://bmjopen.bmj.com/site/about/guidelines.xhtml

For RT-PCR positive patients (followed up for 14 days), hospitalizations and deaths were extracted from the study platform. To extend the follow-up period and to capture RT-PCR negative patients and those initially triaged to hospital (no study follow-up), hospitalization and vital status was confirmed by linkage with two administrative databases: the municipal epidemiological surveillance dataset, as well as the state-wide influenza-like illness notification system (SIVEP-Gripe). Linkage was last performed on 5th June 2020, 23 days after the last patient was enrolled. Categorical patient characteristics were compared according to hospitalization status using a Chi-squared or Fisher exact test. Continuous variables were compared using the Wilcoxon rank sum test.

The cohort sample included consecutive cases presenting to the Corona São Caetano program and a formal sample size calculation was not performed. Missing data were excluded. All analyses were conducted in R Software for Statistical Computing, version 3.6.3.[14]

Ethics

The study was approved by the local ethics committee (Comissão de Ética para Análise de Projeto de Pesquisa - CAPPesq, protocol No. 13915, dated June 03, 2020). The committee waived the need for informed consent and allowed the development of an analytical dataset with no personal identification for the current analysis.

Patient and public involvement

Patients were not involved in the planning of this research.

For peer review only - http://bmjopen.bmj.com/site/about/guidelines.xhtml

RESULTS

Epidemiological and programmatic indicators

Between 13th April and 13th May 2020, there were 2,073 presentations, from 2,011 individual patients, that met the criteria for a suspected COVID-19 case (See Figure 1 for study flow). At initial phone interview, 132 (6%) potential cases were advised to go directly to a health service based on the triage questions, and 12 (0.6%) because of pregnancy. Only four (3%) of referred patients were admitted to hospital and none died.

In total 1,583 individual patients were tested with RT-PCR for SARS-CoV-2; 444 (28.0%, 95%CI 25.9%-30.3%) were positive. The proportion of positive results was stable over the study (Figure S2). Among the RT-PCR negative group, 604 (53% of 1,136) underwent serology testing, of whom 52 (8.6%, 95%CI 6.6% - 11.1%) were seropositive. The median [IQR] time from symptom onset to serology collection was 31 [26 – 37] days. The age-sex structure of patients being tested differed from the underlying population of São Caetano do Sul (Figure S3) with an overrepresentation of working-age adults and women. At the beginning of programme role out, 75% of notified COVID-19 cases in São Caetano do Sul were diagnosed in outpatient or hospital services. Over the study period, adherence to the programme increased, and by May 13th, 2020, 78% of cases in the municipality were diagnosed within our programme.

Of 444 RT-PCR positive patients eligible for longitudinal follow-up, 326 (73%) had their final follow-up visit at least 14 days after their initial presentation. Of the seven possible follow-up questionnaires, 384 (86%) COVID-19 patients completed three or more, and 162 (36%) completed all seven.

Participant characteristics

Patient characteristics are shown in Table 1. Although women were overrepresented in the cohort, there were proportionally more males in the RT-PCR positive and seropositive groups compared to the seronegative group. Of note, 55% of RT-PCR negative/seronegative patients had completed higher education compared to 35% RT-PCR-positive patients ($p < 0.001$, Chi-

For peer review only - http://bmjopen.bmj.com/site/about/guidelines.xhtml

squared test). The median number of days from symptom onset to swab collection was 5.0 (interquartile range [IQR], 4.0-7.0) among RT-PCR positive patients and 6.0 (IQR, 4.0-8.3) among RT-PCR negative/seropositive patients (p = 0.06, Wilcoxon rank sum) (Figure S4). Chronic respiratory disease was less frequent in RT-PCR positive than dual-negative patients.

Symptoms of COVID-19

The prevalence of individual symptoms at presentation is shown in Figure 2A stratified by final diagnostic category. The most frequent symptoms among RT-PCR and seropositive patients were headache (82% and 75%), myalgia (80% and 80%), cough (77% and 63%), and fatigue (77% and 79%). Anosmia was present in 56% and 63% of RT-PCR positive and seropositive patients, respectively, compared to 30% in those testing doubly negative. A similar pattern was observed for ageusia (53% and 53% versus 30%). Upper respiratory tract symptoms - including coryza, blocked nose, ageusia, and anosmia - were more frequent in younger people (Figure 2B). The evolution of symptoms over time among RT-PCR positive patients is shown in Figure S5.

The odds ratios for testing positive for SARS-CoV-2 (RT-PCR or serology) associated with each presenting symptom are shown in Figure 3. The symptoms with strongest associations were anosmia (OR 3.3, 95%CI 2.6-4.4), fever (3.0, 95%CI 2.4-3.9) and ageusia (2.9, 95%CI 2.3-3.8). The presence of sore throat (0.53, 95%CI 0.41-0.68) and diarrhoea (0.72, 95%CI 0.55-0.96) were associated with a negative SARS-CoV-2 test.

Associations between SARS-CoV-2 RT-PCR Cycle threshold (Ct) values, and demographic and clinical features

Figure 4 shows the associations between mean RT-PCR cycle threshold and demographic features and symptoms at presentation. Older age was associated with lower cycle thresholds, with a change in mean Ct of -0.05 (95%CI -0.09 to -0.01) for each additional year of age. The mean difference in Ct value was -1.36 (95% CI -2.49 to -0.23) in men compared to women. For each doubling in the number of days from symptom onset to swab collection the mean Ct value increased by 3.28 (95%CI 2.33 to 4.03). Presenting symptoms of fever and arthralgia were associated with lower Cts, whereas anosmia, ageusia, vomiting, diarrhoea, and nausea were associated with higher Cts (Figure 4 and Table S1). After adjustment for age, sex, delay

For peer review only - http://bmjopen.bmj.com/site/about/guidelines.xhtml

from symptom onset, and RT-PCR platform used, fever (-0.06, 95%CI -2.11 to -0.001) and arthralgia (-1.24, -2.18 to -0.10) remained associated with lower Cts, and anosmia (2.21, 1.0 to 3.29), ageusia (1.96, 0.88 to 3.0), and diarrhoea (1.36, 0.12 to 2.61) with higher Cts (Table S1).

Hospitalizations and deaths

Of the 444 RT-PCR positive patients, 30 (6.8%) had been hospitalized by 5th June 2020, when the database linkage was last updated, and three (0.7%) had died; in-hospital mortality was therefore 10% (3/30). In 28 cases the date of admission was available. The median time from symptom onset to hospital admission was 7 (range 2 to 14) days. Among 1,136 RT-PCR-negative patients, six (0.5%) had been admitted to hospital. One (<0.01% of 1,136) of these six patients died. None of the 604 RT-PCR negative patients that underwent serology were admitted to hospital or died. Table 2 compares patient characteristics by hospitalization status. Notably, hospitalized patients were older, had more cardiovascular comorbidities and were more frequently obese.

For peer review only

DISCUSSION

We present a community-based cohort of suspected COVID-19 cases recruited through a primary care initiative in the Brazilian municipality of São Caetano do Sul. Offering RT-PCR testing to all patients presenting with symptoms compatible with COVID-19, the positivity rate was 28%, with 8.6% of those testing negative subsequently found to be seropositive - i.e. > 35% of the cohort had a diagnosis of COVID-19. Anosmia, ageusia, and self-reported fever provided the greatest diagnostic value in identifying COVID-19. The rate of hospitalization and deaths among RT-PCR positive patients was low, at 6.8% and 0.7%, respectively. Our results provide important information on the clinical presentation, diagnostic testing and natural history of COVID-19 identified in the community.

Extrapolating the seropositivity rate among RT-PCR negative patients to the 532 that were not tested with serology, we estimate that an additional 46 seropositive cases would have been identified. This corresponds to a false-negative rate of 18% among potential symptomatic COVID-19 cases. This is lower than a recent pooled analysis: nadir of 20% at three days post-symptom onset.[15] Viral load peaks around the time of symptom onset and remains high over the first symptomatic week (also see Figure 4A).[16,17] Consistent with this, we found a slightly longer delay to swab collection in RT-PCR false-negative patients than RT-PCR positive patients (Figure S4).

COVID-19 presents in a similar way to other respiratory viral illnesses. Indeed, in our cohort the most common symptoms of COVID-19 - such as cough, fatigue, headache, etc. - were reported with a similar frequency among patients testing negative. It is therefore important to have identified anosmia, ageusia, self-reported fever, myalgia, and anorexia as the symptoms with greatest value in the differential diagnosis of COVID-19 in primary care. Conversely, sore throat and diarrhoea - both considered symptoms of COVID-19 in other settings –[18] were more frequently due to other aetiologies in this primary care context. These results are robust for a number of reasons. Firstly, our sample is representative of the population of interest - i.e. consecutive patients with suspected COVID-19 in the community - instead of extrapolating from hospital cases. Symptom data were collected prospectively, eliminating recall or interviewer bias. Finally, we have a control group of patients who were negative for both RT-PCR and serology, minimizing misclassification due to false negative RT-PCR.

For peer review only - http://bmjopen.bmj.com/site/about/guidelines.xhtml

In our study, the proportion of patients with a positive SARS-CoV-2 RT-PCR requiring hospitalization was low (7%). Early reports from China were of 13.8% of cases being severe[19], but this value was lower when under ascertainment of cases was accounted for.[20,21] This is because our cohort reflects mild to moderate cases, as severely ill patients are likely to have attended hospital directly. As such, only 3% of patients we triaged to attend health services were ultimately hospitalized, possibly due to self-selection of patients presenting to our service. Supporting this notion, our overall case fatality ratio among RT-PCR positive patients was 0.7%.

Our study has some limitations. Serology was not performed on all RT-PCR negative patients due to on-going symptoms, loss to follow-up, or patient refusal. Of note, none of the RT-PCR-negative patients that were admitted to hospital underwent serology testing. This suggests that patients who were not tested with serology may have had a higher prevalence of COVID-19 than those that were tested. In addition, imperfect serology test performance (81% sensitivity)[13] will introduced false-negative results. Taken together, these biases may have underestimated the true seroprevalence among RT-PCR-negative cases, as well as the false-negative rate of RT-PCR. The latter calculation may also have been influenced by the inclusion of RT-PCR positive patients in the denominator, introducing an incorporation bias.[22]

A key strength to our study relates to the provision of primary healthcare in Brazil and its symbiosis with medical training nationwide. Primary health care - within the family health strategy (*Estratégia Saúde da Família*) - is centered around a healthcare unit with a multi-professional team that is responsible for all residents in the immediate catchment area [23]. São Caetano do Sul has 100% coverage with the family health strategy, and medical students from the municipal university (USCS) are integrated into the healthcare teams and progressively trained from the first year of medical school. Our initiative took advantage of this existing system, with the addition of an online platform allowing remote clinical assessment and follow-up. The suspension of normal clinical training at the medical school provided the workforce. The partnership with the University of São Paulo, which provided the laboratory diagnostics, created the unique opportunity to establish our prospective community cohort of suspected and confirmed COVID-19 cases. But we believe that this infrastructure can be implemented in other regions with less resources. Other respiratory disease such as influenza, measles, or tuberculosis may benefit from similar approach.

For peer review only - http://bmjopen.bmj.com/site/about/guidelines.xhtml

CONTRIBUTION STATEMENT

FL, MC, SC, MC, RB, and ES conceived and designed the study. FL, RG, and JB provided clinical oversight and supervision of medical students. FL, MC, LB, HD, and SS collected and curated the data. MC, TM, LV, and LS performed the laboratory analysis. LB performed the formal statistical analysis with assistance from FL, SS, NA, PM, ES. LB, FL, PM and ES wrote the first draft, and all authors reviewed, contributed to and approved the final version.

CONFLICTS OF INTEREST STATEMENT

The authors have no conflicts of interests.

FUNDING STATEMENT

The municipal health department of São Caetano do Sul (Secretaria Municipal de Saúde da Prefeitura de São Caetano do Sul) funded the establishment and implementation of the platform. We also acknowledge an award from FAPESP (2018/14389-0) and the UK Medical Research Council (MR/S0195/1) to the Brazil-UK Centre for Arbovirus Discovery, Diagnosis, Genomics and Epidemiology (CADDE).

DATA SHARING STATEMENT

Data will be made available by reasonable request to the corresponding author and following local ethnics approval.

For peer review only

REFERENCES

1 World Health Organization. (2020). Pneumonia of unknown cause – China. (https://www.who.int/csr/don/05-january-2020-pneumonia-of-unkown-cause-china/en/, accessed 2020-06-18).

2 World Health Organization. Critical preparedness, readiness and response actions for COVID-19 (Interim Guidance) (https://www.who.int/publications-detail/critical-preparedness-readiness-and-response-actions-for-covid-19, accessed 15 June 2020).

3 Hellewell J, Abbott S, Gimma A, et al. Feasibility of controlling COVID-19 outbreaks by isolation of cases and contacts. Lancet Glob Health 2020; 8: e488–96.

4 World Health Organization. Regional Office for the Western Pacific. (2020). Role of primary care in the COVID-19 response. (https://apps.who.int/iris/handle/10665/331921, accessed 15 June, 2020).

5 World Health Organization. Situation report 27 (2020). (https://www.who.int/emergencies/diseases/novel-coronavirus-2019/situation-reports, accessed 15 June 2020).

6 Brazilian Ministry of Health. (https://covid.saude.gov.br/, accessed 15 June 2020).

7 Docherty AB, Harrison EM, Green CA, et al. Features of 20 133 UK patients in hospital with covid-19 using the ISARIC WHO Clinical Characterisation Protocol: prospective observational cohort study. BMJ 2020; m1985.

8 Richardson S, Hirsch JS, Narasimhan M, et al. Presenting Characteristics, Comorbidities, and Outcomes Among 5700 Patients Hospitalized With COVID-19 in the New York City Area. JAMA 2020; **323**: 2052.

9 Zhou F, Yu T, Du R, et al. Clinical course and risk factors for mortality of adult inpatients with COVID-19 in Wuhan, China: a retrospective cohort study. Lancet 2020; **395**: 1054–62.

10 Rauh AL, Linder JA. Covid-19 care before, during, and beyond the hospital. BMJ 2020; m2035.

11 Instituto Brasileiro de Geografia e Estatística (IBGE). (2020). São Caetano do Sul / Panorama. (https://cidades.ibge.gov.br/brasil/sp/sao-caetano-do-sul/panorama, accessed 20 June 2020).

12 Human Development Atlas Brazil. (2020). São Caetano do Sul (http://atlasbrasil.org.br/2013/pt/perfil_m/sao-caetano-do-sul_sp, accessed 20 June 2020).

13 Whitman JD, Hiatt J, Mowery CT, et al. Test performance evaluation of SARS-CoV-2 serological assays. medRxiv 2020; 2020.04.25.20074856.

14 R Core Team. R: A language and environment for statistical computing. R Foundation for Statistical Computing, Vienna, Austria. (https://www.R-project.org/. 2019).

15 Kucirka LM, Lauer SA, Laeyendecker O, Boon D, Lessler J. Variation in False-Negative Rate of Reverse Transcriptase Polymerase Chain Reaction–Based SARS-CoV-2 Tests by Time Since Exposure. Ann Int Med 2020; M20-1495.

16 To KK-W, Tsang OT-Y, Leung W-S, et al. Temporal profiles of viral load in posterior oropharyngeal saliva samples and serum antibody responses during infection by SARS-CoV-2: an observational cohort study. Lancet Inf Dis 2020; **20**: 565–74.

17 He X, Lau EHY, Wu P, et al. Temporal dynamics in viral shedding and transmissibility of COVID-19. Nat Med 2020; **26**: 672–5.

18 Vetter P, Vu DL, L'Huillier AG, Schibler M, Kaiser L, Jacquerioz F. Clinical features of covid-19. BMJ 2020; m1470.

For peer review only

19 T. Team, Vital Surveillances: The Epidemiological Characteristics of an Outbreak of 2019 Novel Coronavirus Diseases (COVID-19)-China. China CDC Weekly 2020; **2**: 113–22.
20 Salje H, Tran Kiem C, Lefrancq N, et al. Estimating the burden of SARS-CoV-2 in France. Science 2020; eabc3517.
21 Verity R, Okell LC, Dorigatti I, et al. Estimates of the severity of coronavirus disease 2019: a model-based analysis. Lancet Infect Dis 2020; **20**: 669–77.
22 Worster A, Carpenter C. Incorporation bias in studies of diagnostic tests: how to avoid being biased about bias. CJEM 2008; **10**: 174–5.
23 Macinko J, Harris MJ. Brazil's Family Health Strategy — Delivering Community-Based Primary Care in a Universal Health System. NEJM 2015; **372**: 2177–81.

TABLE LEGENDS

Table 1 Demographic and clinical characteristics of 1,048 suspected COVID-19 cases undergoing diagnostic testing in the Corona São Caetano program. * Security, emergency services, supermarket, public transport, and pharmacy workers. IQR: interquartile range; HCW: health care workers, COPD: chronic obstructive pulmonary disease. Missing data – educational level 2; essential occupation 2; body mass index 4; cardiovascular disease 28; diabetes 31 mellitus; chronic resp. disease 65; chronic kidney disease 27; COPD 28. P-values calculated by Chi-squared, Fisher exact, or Wilcoxon rank sum.

Table 2 Characteristics of RT-PCR positive patients stratified by hospitalization status. Missing data – body mass index 2; cardiovascular disease 12; diabetes mellitus 12; chronic respiratory disease 29; COPD 11; chronic kidney disease 12; COPD - chronic obstructive pulmonary disease; IQR - interquartile range.

FIGURE LEGENDS

Figure 1 Patient flowchart for the Corona São Caetano platform between 13th April and 13th May 2020. In the upper section (white background) the numbers correspond to individual presentations to the system; among suspected cases 2,073 suspected cases, 60 had two presentations and one had three. In the lower section (grey background) numbers correspond to individual patients making up the final analytic groups.

Figure 2 Panel A presents prevalence (point) and exact binomial 95% confidence intervals (vertical lines) of symptoms at presentation among patients with suspected COVID-19 according to RT-PCR result and serostatus (A). Panels B and C present the prevalence of

For peer review only

presenting symptoms among patients with COVID-19 (RT-PCR and serology positive) stratified by age (B) and sex (C).

Figure 3 Odds ratios (black dot) and 95% confidence intervals (lines) for testing positive for COVID-19 (RT-PCR positive or serology positive) associated with the presence of each presenting symptom. Horizontal axis is on log scale. Point estimates of odds ratios are shown inline with their corresponding symptom.

Figure 4 Relationship between mean RT-PCR cycle threshold (Ct) and day of illness course when the nasopharyngeal swab was collected (A), patient age (B), patient sex (C), and different symptoms at presentation. Panels A and B show the best fit linear regression lines, panels C and D are violin plots (rotated kernel density plots showing the full distribution of data) of the Ct values with median (black dot) and interquartile range (black line).

For peer review only

Table 1

	**RT-PCR +ve (G1)** N = 444 n (%) or median (IQR)	**RT-PCR -ve Sero +ve (G2)** N=52 n (%) or median (IQR)	**RT-PCR -ve Sero -ve (G3)** N = 552 n (%) or median (IQR)	**p-value** G1 versus G2	**p-value** G1 versus G3
**Sex**					
Male	200 (45·0)	23 (44·2)	185 (33·5)		
Female	244 (55·0)	29 (55·8)	367 (66·5)	1·0	<0·001
**Age groups (years)**					
10 to 19	29 (6·5)	1 (1·9)	25 (4·5)		
20 to 39	197 (44·4)	17 (32·7)	236 (42·8)		
40 to 59	158 (35·6)	28 (53·8)	218 (39·5)		
60+	60 (13·5)	6 (11·5)	73 (13·2)	0·07	0·40
**Educational level**					
Up to primary education	75 (16·9)	7 (13·5)	56 (10·2)		
High school	214 (48·3)	19 (36·5)	194 (35·2)		
University	154 (34·8)	26 (50·0)	301 (54·6)	0·10	<0·001
**Essential Occupation**					
Non-HCW essential job *	137 (30·9)	12 (23·1)	148 (26·9)		
Carers	10 (2·3)	0 (0·0)	8 (1·5)		
HCW	32 (7·2)	5 (9·6)	73 (13·2)		
No	264 (59·6)	35 (67·3)	322 (58·4)	0·45	0·01
**Body mass index (kg/m²)**					
<25	151 (34·2)	22 (42·3)	211 (38·4)		
25-29	182 (41·2)	17 (32·7)	187 (34·0)		
30-35	79 (17·9)	9 (17·3)	112 (20·4)		
35+	30 (6·8)	4 (7·7)	40 (7·3)	0·62	0·14
**Comorbidities**					
Cardiovascular disease	88 (20·4)	9 (17·6)	129 (24·0)	0·89	0·40
Diabetes mellitus	48 (11·1)	4 (7·8)	39 (7·3)	0·86	0·12
Any chronic resp. disease	37 (8·9)	9 (18·0)	79 (15·3)	0·13	0·01
COPD	24 (5·5)	5 (9·8)	54 (10·1)	0·47	0·03
Chronic kidney disease	1 (<1)	0 (0·0)	3 (1·0)	1·0	0·83
**Time from symptom onset to swab collection (days)**, median (IQR)	5·0 (4·0-7·0)	6·0 (4·0-8·3)	6·0 (4·0-9·0)	0·06	<0·001

For peer review only

Table 2

	Hospitalized<br>n=30<br>n (%) or median (IQR)	Not hospitalized<br>n=414<br>n (%) or median (IQR)	p-value
**Age (years)**			
10 to 19	1 (3)	28 (97)	
20 to 39	6 (3)	191 (97)	
40 to 59	14 (9)	144 (91)	
60+	9 (15)	51 (85)	0·006
**Sex**			
Female	16 (7)	228 (93)	
Male	14 (7)	186 (93)	0·852
**Comorbidities**			
Cardiovascular disease	11 (13)	77 (87)	0·001
Diabetes mellitus	8 (17)	40 (83)	0·007
Any chronic resp. disease	2 (5)	35 (95)	1·0
COPD	1 (5)	23 (95)	1·0
Chronic kidney disease	1 (100)	0 (0)	0·06
**Body mass index (Kg/m²)**			
<25	4 (3)	147 (97)	
25-29	8 (4)	174 (96)	
30-35	12 (15)	67 (85)	
35+	6 (20)	24 (80)	<0·001
**Time to presentation (days)**	3 (3 to 4)	4 (3 to 5)	0·072

For peer review only

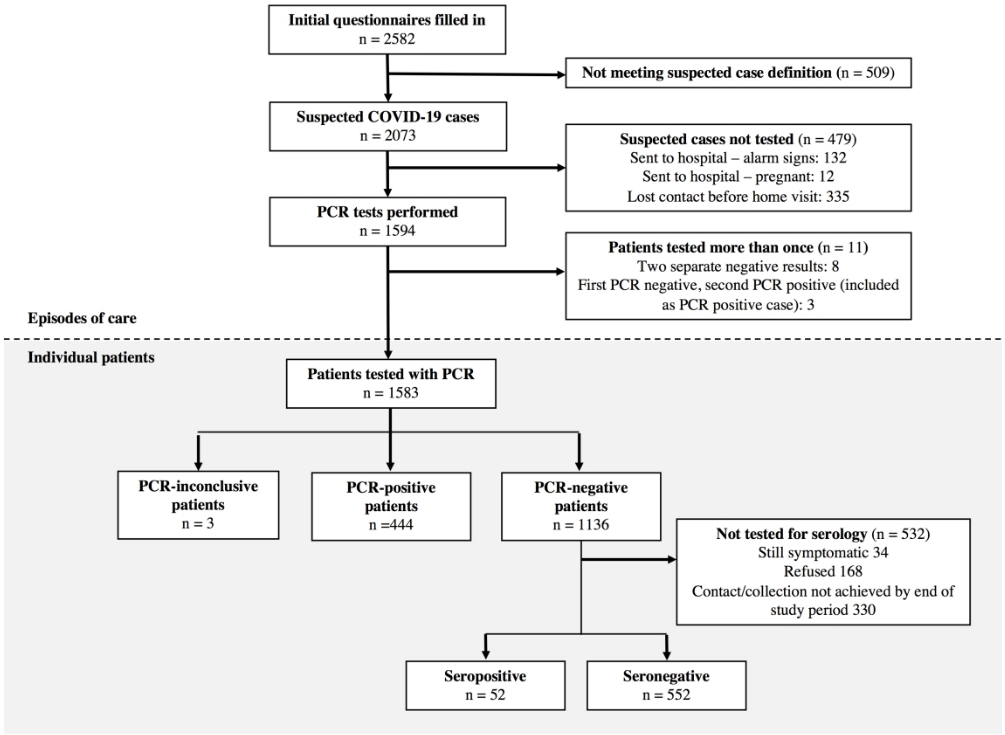

Figure 1

246x181mm (300 x 300 DPI)

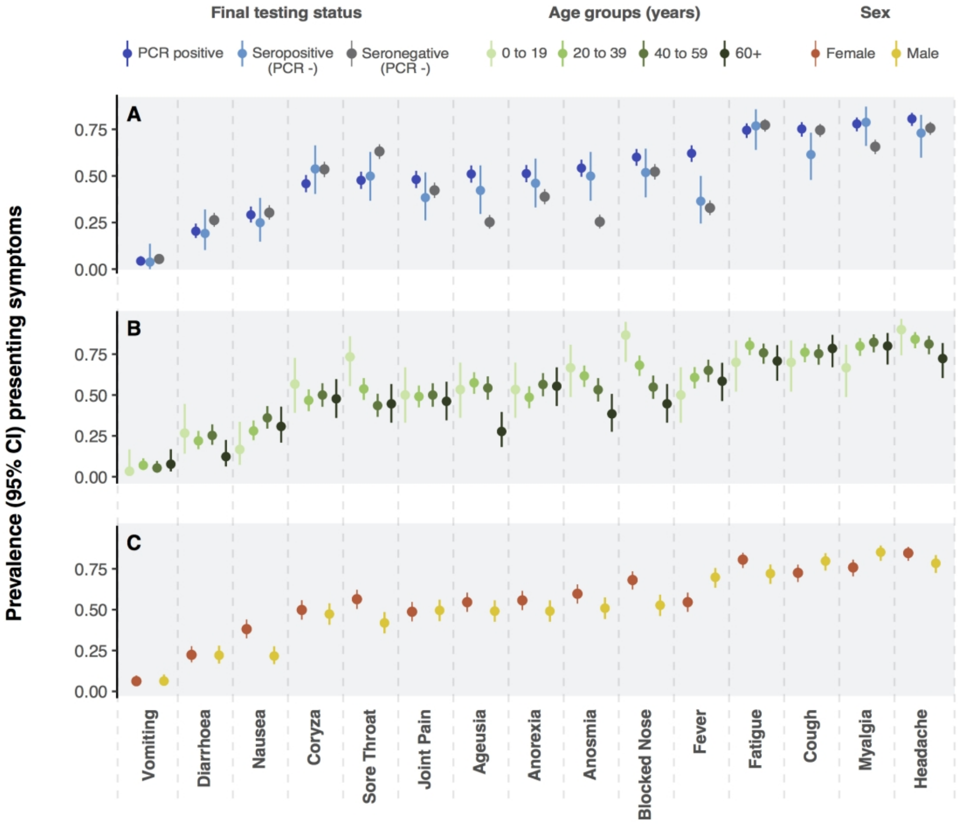

Figure 2

187x158mm (300 x 300 DPI)

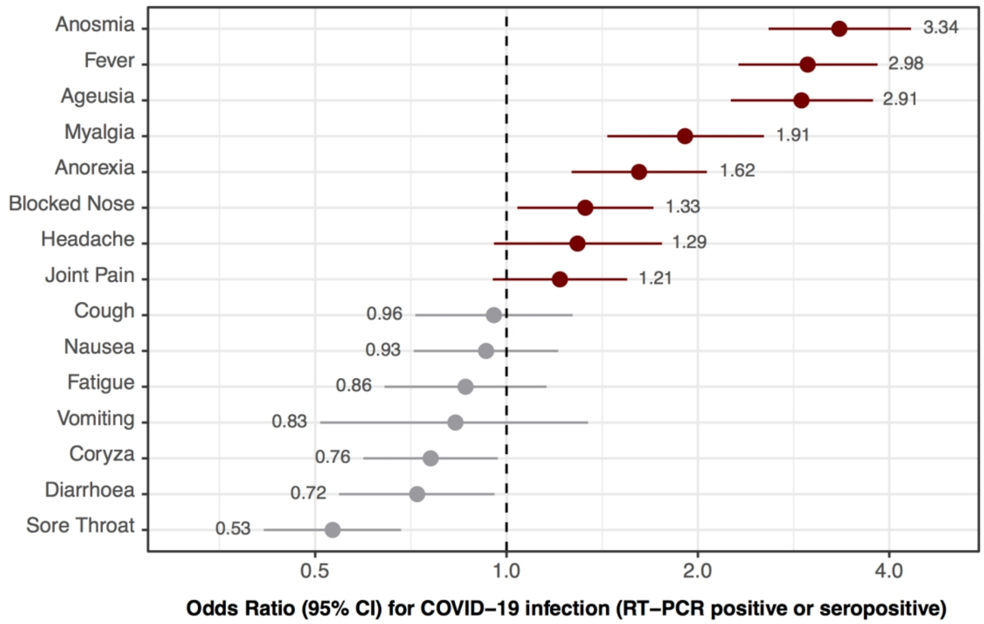

Figure 3

154x100mm (300 x 300 DPI)

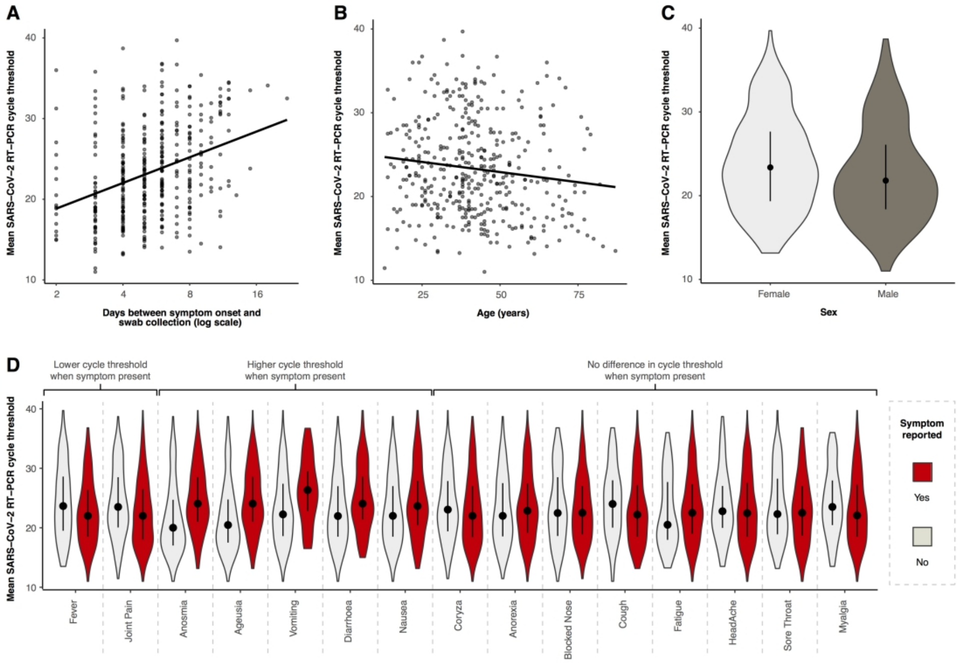

Figure 4

327x222mm (300 x 300 DPI)

Supplemental material

Initial risk assessment

Patients meeting the definition of a suspected case were called by a medical student (under supervision) to complete a risk assessment. All patients were asked a set of standardized questions:

- Do you feel short of breath?
- Are you breathing quickly or finding it difficult to breath?
- If yes, can you count your respiratory rate over one minute? (respiratory rate >20 breaths/minute was considered tachypnoea)
- Has your fever worsened over the last 3 days or have you had a new fever after 2 days being fever-free?
- Have you felt confused or lethargic?

If the patient answered "yes" to any of these questions they were advised to attend a specialist health service. Among the 132 patients that were triaged to hospital, 76 (58% of 132) had shortness of breath, 76 (58% of 132) reported rapid breathing, 33 (25% of 132) persistent fever and 22 (17% of 132) altered mental status.

For peer review only

Table S1 Univariable and adjusted associations between RT-PCR cycle thresholds and patient characteristics

	**Unadjusted analysis**		**Adjusted analysis ***	
	**Beta** (difference in means)	**95% Confidence interval**	**Beta** (difference in means)	**95% Confidence interval**
**Age** (years)	**-0.05**	**-0.09 to -0.01**	**-0.06**	**-0.09 to -0.03**
**Male sex**	**-1.36**	**-2.49 to -0.23**	-1.05	-2.09 to <0.001
**Days from symptom onset to swab collection** (days, log$_2$)	**3.28**	**2.33 to 4.03**	**3.27**	**0.42 to 7.70**
**PCR platform** (ALTONA as reference)	**-1.19**	**-2.37 to -0.02**	**-1.53**	**-2.6 to -0.45**
**Symptoms at presentation**				
Fever	**-1.78**	**-2.96 to -0.59**	**-1.11**	**-2.11 to -0.001**
Myalgia	-1.31	-2.75 to 0.12	-0.78	-2.11 to 0.53
Arthralgia	**-1.64**	**-2.77 to -0.52**	**-1.24**	**-2.18 to -0.10**
Anosmia	**3.15**	**2.04 to 4.25**	**2.21**	**1.0 to 3.29**
Agusia	**2.99**	**1.89 to 4.09**	**1.96**	**0.88 to 3.0**
Diarrhea	**2.19**	**0.84 to 3.53**	**1.36**	**0.12 to 2.61**
Nausea	**1.50**	**0.28 to 2.72**	1.09	-0.04 to 2.24
Vomiting	**2.99**	**0.52 to 5.46**	2.02	-0.28 to 4.33
Anorexia	0.56	-0.57 to 1.70	0.47	-0.58 to 1.51
Headache	-0.58	-2.12 to 0.97	-0.81	-2.25 to 0.63
Fatigue	0.84	-0.50 to 2.18	0.34	-0.91 to 1.59
Coryza	-0.78	-1.92 to 0.34	-0.68	-1.72 to 0.34
Blocked nose	-0.36	-1.53 to 0.81	**-1.48**	**-2.59 to -0.37**
Cough	-1.33	-2.70 to 0.03	**-1.60**	**-2.86 to -0.33**
Sore throat	-0.49	-1.62 to 0.64	-0.45	-1.52 to 0.61

* All variables adjusted for age (continuous in years), sex (female as reference group), PCR platform (ALTONA platform as the reference group) and time between symptom onset and swab collection (log base 2). Analysis was performed within a linear regression framework. Positive beta coefficients indicate higher cycle thresholds (lower viral load) associated with that variable, whereas negative beta coefficients indicate lower cycle thresholds when the variable is present. Results in bold reached statistical significance.

For peer review only

Supplemental figure legends

Figure S1 Comparison of cycle thresholds across PCR platforms and genes amplified. Upper two panels show the concordance between cycle thresholds for the two separate genes amplified by the ALTONA (left) and Mico Biomed (right) kits. Lower left panel – distribution of cycle thresholds by gene amplified and RT-PCR platform used. Lower right-hand panel – distribution of the mean cycle threshold (mean of cycle thresholds for separate genes) between different RT-PCR platforms.

Figure S2 Time series of presentations to the Corona São Caetano platform. Dashed vertical lines denote the weekends with a reduced number of presentations. Thick black line corresponds to the right-hand y-axis: proportion of RT-PCRs performed with positive result.

Figure S3 Age-sex distribution the city of São Caetano do Sul compared with that of patients accessing the Corona São Caetano system and being tested with RT-PCR (left-hand panel) and those testing positive for SARS-CoV-2 (right-hand panel).

Figure S4 Distribution of delay between symptom onset and swab collection among 444 RT-PCR-positive patients and 52 RT-PCR-negative patients that subsequently tested seropositive (left-hand panel). Histogram of delay between symptom onset and swab collection among patients with COVID-19 (right-hand panel).

Figure S5 Left hand figures show symptoms at each follow-up questionnaire among patients testing RT-PCR positive and undergoing follow-up. Individual patients are stacked on the y-axis ordered according to the delay from symptom onset to presentation. Each point represents the response to a questionnaire and its position on the horizontal axis the time after symptom onset that the questionnaire was filled in. Grey points are questionnaires where the patient denied the presence of a given symptom. The coloured points correspond to questionnaires in which the patient reported a given symptom. The right-hand figures results from grouping the horizontal axis time into two-day windows and calculating the proportion of completed questionnaires in which each symptom was reported. The denominators for the horizontal axis groups (number of questionnaires completed within a given time window from symptom onset) are 104 at [0-2] days, 192 at (2-4], 185 at (4-6], 293 at (6-8], 338 at (8-10], 329 at (10-12], 335 at (12-14], 324 at (14-16], 280 at (16-18] and 201 at (18-20].

For peer review only

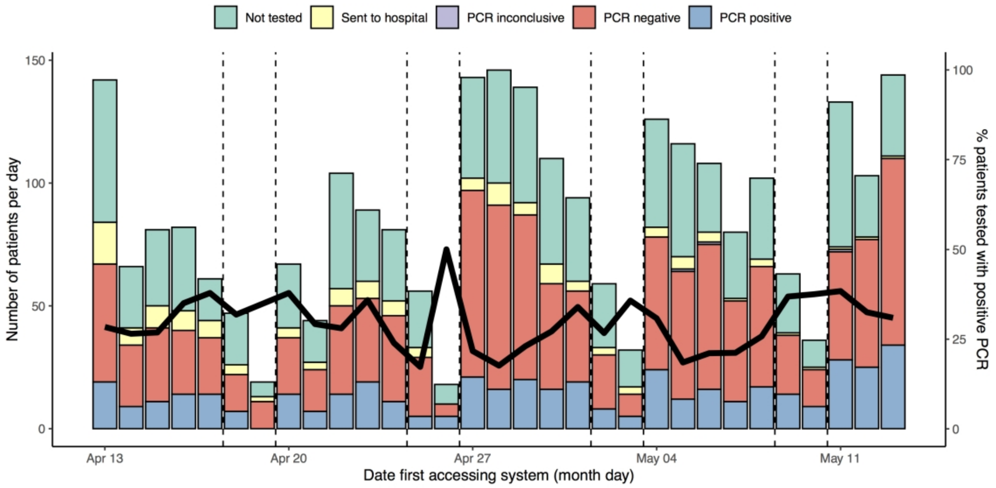

Figure S2

254x127mm (300 x 300 DPI)

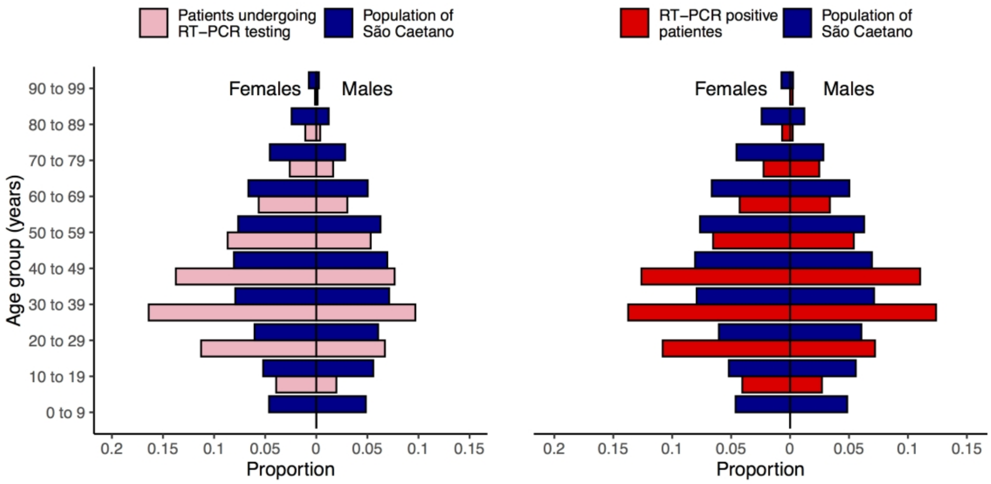

Figure S3

203x101mm (300 x 300 DPI)

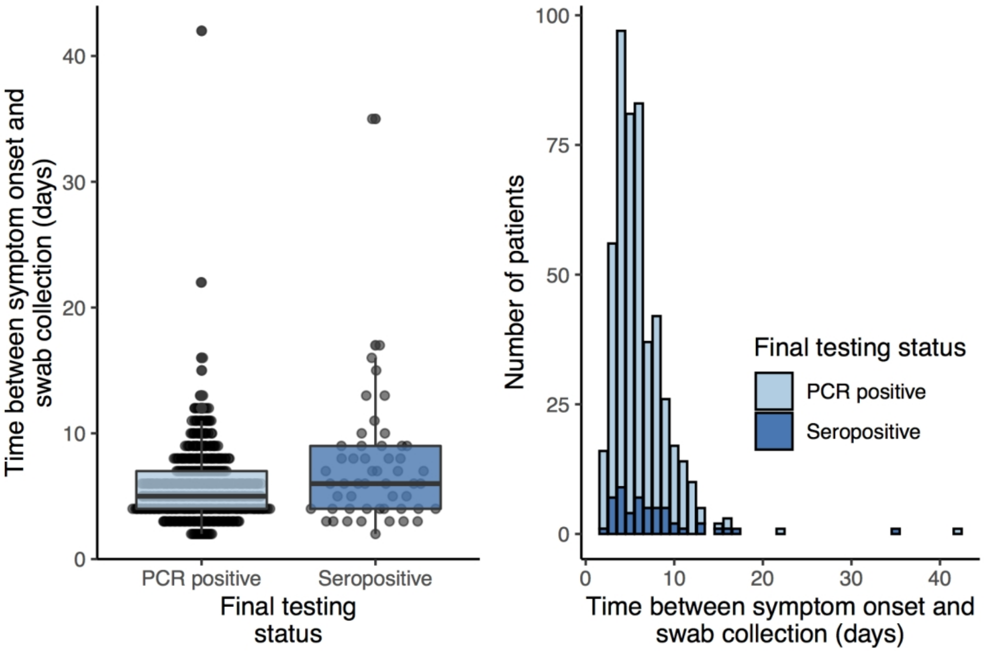

Figure S4

152x101mm (300 x 300 DPI)

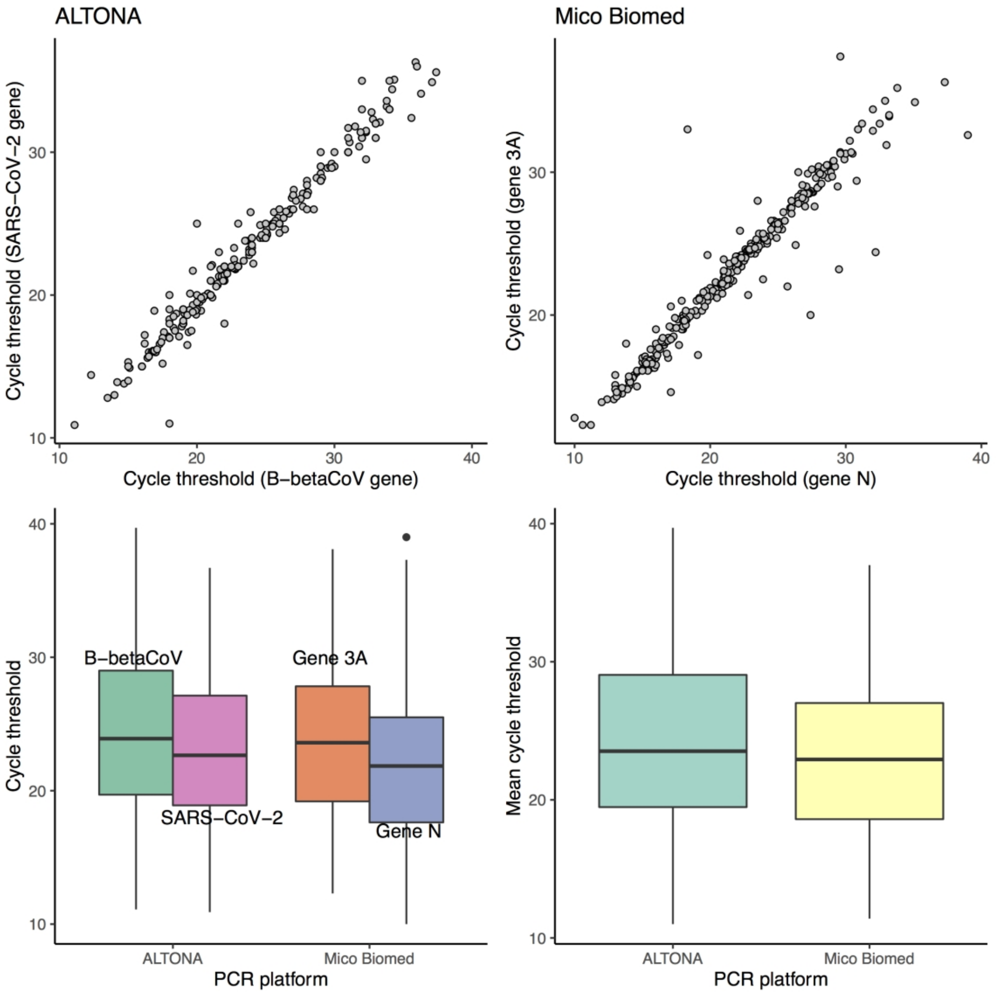

Figure S1

203x203mm (300 x 300 DPI)

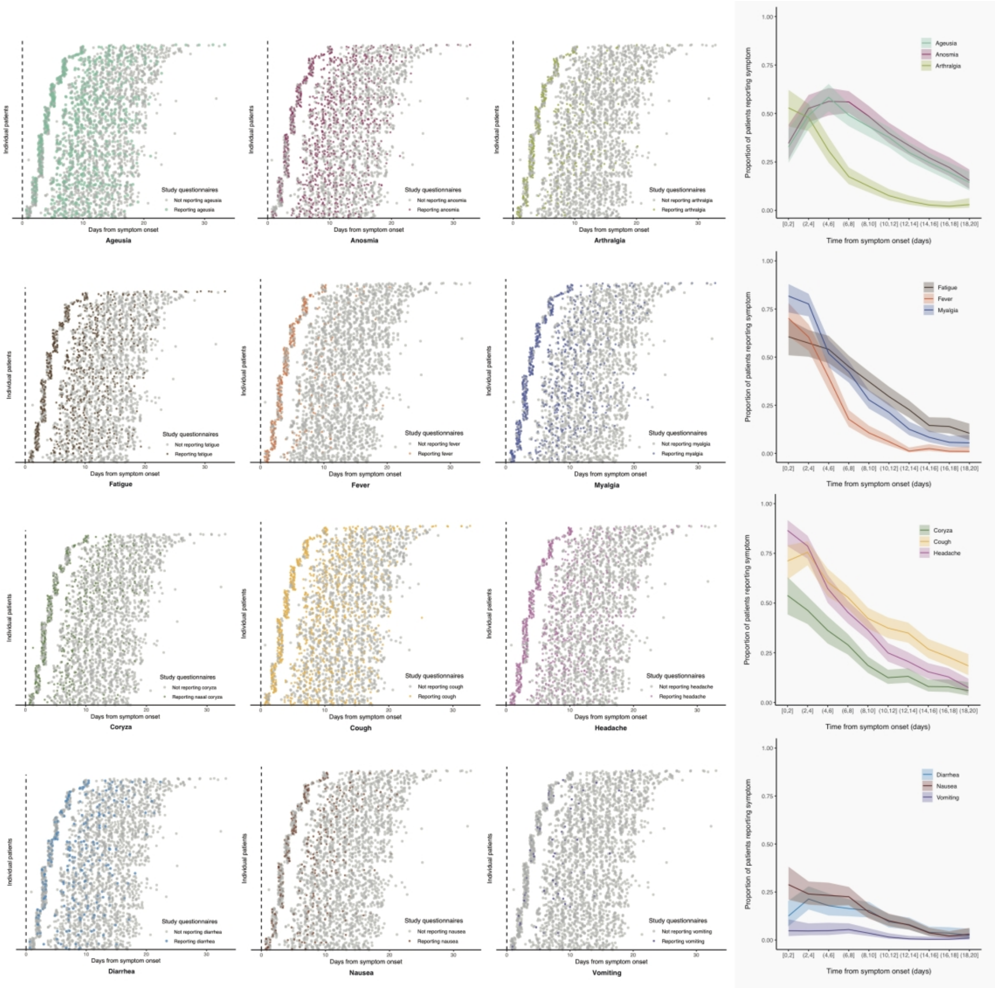

Figure S5

433x427mm (150 x 150 DPI)

STROBE Statement—Checklist of items that should be included in reports of ***cohort studies***

	Item No	Recommendation	Page No
**Title and abstract**	1	(*a*) Indicate the study's design with a commonly used term in the title or the abstract<br>(*b*) Provide in the abstract an informative and balanced summary of what was done and what was found	1 and 2
**Introduction**			
Background/rationale	2	Explain the scientific background and rationale for the investigation being reported	4
Objectives	3	State specific objectives, including any prespecified hypotheses	4
**Methods**			
Study design	4	Present key elements of study design early in the paper	5 to 7
Setting	5	Describe the setting, locations, and relevant dates, including periods of recruitment, exposure, follow-up, and data collection	5 to 6
Participants	6	(*a*) Give the eligibility criteria, and the sources and methods of selection of participants. Describe methods of follow-up<br>(*b*) For matched studies, give matching criteria and number of exposed and unexposed	A – 5<br>B - NA
Variables	7	Clearly define all outcomes, exposures, predictors, potential confounders, and effect modifiers. Give diagnostic criteria, if applicable	7 to 8
Data sources/ measurement	8*	For each variable of interest, give sources of data and details of methods of assessment (measurement). Describe comparability of assessment methods if there is more than one group	5 to 7
Bias	9	Describe any efforts to address potential sources of bias	7 to 8
Study size	10	Explain how the study size was arrived at	8
Quantitative variables	11	Explain how quantitative variables were handled in the analyses. If applicable, describe which groupings were chosen and why	
Statistical methods	12	(*a*) Describe all statistical methods, including those used to control for confounding<br>(*b*) Describe any methods used to examine subgroups and interactions<br>(*c*) Explain how missing data were addressed<br>(*d*) If applicable, explain how loss to follow-up was addressed<br>(*e*) Describe any sensitivity analyses	A – 7 to 8<br>B – NA<br>C – 8<br>D – NA<br>E – NA
**Results**			
Participants	13*	(a) Report numbers of individuals at each stage of study—eg numbers potentially eligible, examined for eligibility, confirmed eligible, included in the study, completing follow-up, and analysed<br>(b) Give reasons for non-participation at each stage<br>(c) Consider use of a flow diagram	Figure 1 and page 9
Descriptive data	14*	(a) Give characteristics of study participants (eg demographic, clinical, social) and information on exposures and potential confounders<br>(b) Indicate number of participants with missing data for each variable of interest	A – table 1 and pages 9to10<br>B - Table 1 and 2 legends

For peer review only - http://bmjopen.bmj.com/site/about/guidelines.xhtml

		(c) Summarise follow-up time (eg, average and total amount)	C – page 9
Outcome data	15*	Report numbers of outcome events or summary measures over time	11
Main results	16	(*a*) Give unadjusted estimates and, if applicable, confounder-adjusted estimates and their precision (eg, 95% confidence interval). Make clear which confounders were adjusted for and why they were included<br>(*b*) Report category boundaries when continuous variables were categorized<br>(*c*) If relevant, consider translating estimates of relative risk into absolute risk for a meaningful time period	Page 7 and results section
Other analyses	17	Report other analyses done—eg analyses of subgroups and interactions, and sensitivity analyses	NA
**Discussion**			
Key results	18	Summarise key results with reference to study objectives	12
Limitations	19	Discuss limitations of the study, taking into account sources of potential bias or imprecision. Discuss both direction and magnitude of any potential bias	13
Interpretation	20	Give a cautious overall interpretation of results considering objectives, limitations, multiplicity of analyses, results from similar studies, and other relevant evidence	13
Generalisability	21	Discuss the generalisability (external validity) of the study results	13
**Other information**			
Funding	22	Give the source of funding and the role of the funders for the present study and, if applicable, for the original study on which the present article is based	14

*Give information separately for exposed and unexposed groups.

Note: An Explanation and Elaboration article discusses each checklist item and gives methodological background and published examples of transparent reporting. The STROBE checklist is best used in conjunction with this article (freely available on the Web sites of PLoS Medicine at http://www.plosmedicine.org/, Annals of Internal Medicine at http://www.annals.org/, and Epidemiology at http://www.epidem.com/). Information on the STROBE Initiative is available at http://www.strobe-statement.org.

For peer review only

BMJ Open

Clinical features and natural history of the first 2,073 suspected COVID-19 cases in the Corona São Caetano primary care programme: a prospective cohort study

Journal:	*BMJ Open*
Manuscript ID	bmjopen-2020-042745.R1
Article Type:	Original research
Date Submitted by the Author:	11-Nov-2020

Complete List of Authors:	Leal, Fabio ; Universidade Municipal de São Caetano do Sul; Instituto Nacional de Cancer<br>Mendes-Correa, Maria; Universidade de São Paulo, Instituto de Medicina Tropical (LIM-52, LIM-46, LIM-49) and Departamento de Moléstias Infecciosas e Parasitarias<br>Buss, Lewis; Universidade de São Paulo, Instituto de Medicina Tropical (LIM-52, LIM-46, LIM-49) and Departamento de Moléstias Infecciosas e Parasitarias<br>Costa, Silvia; Universidade de São Paulo, Instituto de Medicina Tropical (LIM-52, LIM-46, LIM-49) and Departamento de Moléstias Infecciosas e Parasitarias<br>Bizario, Joao; Universidade Municipal de São Caetano do Sul<br>de Souza, Sonia ; Universidade Municipal de São Caetano do Sul<br>Thomaz, Osorio; Instituto de Pesquisas Tecnológicas<br>Tozetto-Mendoza, Tania; Universidade de São Paulo, Instituto de Medicina Tropical (LIM-52, LIM-46, LIM-49) and Departamento de Moléstias Infecciosas e Parasitarias<br>Villas-Boas, Lucy; Universidade de São Paulo, Instituto de Medicina Tropical (LIM-52, LIM-46, LIM-49) and Departamento de Moléstias Infecciosas e Parasitarias<br>Oliveira, Lea; Universidade de São Paulo, Instituto de Medicina Tropical (LIM-52, LIM-46, LIM-49) and Departamento de Moléstias Infecciosas e Parasitarias<br>Grespan, Regina; Universidade Municipal de São Caetano do Sul<br>Capuani, Ligia; Modular Research System<br>Buccheri, Renata; Universidade de São Paulo, Instituto de Medicina Tropical (LIM-52, LIM-46, LIM-49) and Departamento de Moléstias Infecciosas e Parasitarias<br>Domingues, Helves; Modular Research System<br>Alexander, Neal; London School of Hygiene & Tropical Medicine<br>Mayaud, Philippe; London School of Hygiene & Tropical Medicine<br>Sabino, Ester; Universidade de São Paulo, Instituto de Medicina Tropical (LIM-52, LIM-46, LIM-49) and Departamento de Moléstias Infecciosas e Parasitarias

**Primary Subject Heading**:	Infectious diseases
Secondary Subject Heading:	Epidemiology, Public health, Global health

Keywords:	PRIMARY CARE, Epidemiology < TROPICAL MEDICINE, INFECTIOUS DISEASES, PUBLIC HEALTH

SCHOLARONE™
Manuscripts

I, the Submitting Author has the right to grant and does grant on behalf of all authors of the Work (as defined in the below author licence), an exclusive licence and/or a non-exclusive licence for contributions from authors who are: i) UK Crown employees; ii) where BMJ has agreed a CC-BY licence shall apply, and/or iii) in accordance with the terms applicable for US Federal Government officers or employees acting as part of their official duties; on a worldwide, perpetual, irrevocable, royalty-free basis to BMJ Publishing Group Ltd ("BMJ") its licensees and where the relevant Journal is co-owned by BMJ to the co-owners of the Journal, to publish the Work in this journal and any other BMJ products and to exploit all rights, as set out in our licence.

The Submitting Author accepts and understands that any supply made under these terms is made by BMJ to the Submitting Author unless you are acting as an employee on behalf of your employer or a postgraduate student of an affiliated institution which is paying any applicable article publishing charge ("APC") for Open Access articles. Where the Submitting Author wishes to make the Work available on an Open Access basis (and intends to pay the relevant APC), the terms of reuse of such Open Access shall be governed by a Creative Commons licence – details of these licences and which Creative Commons licence will apply to this Work are set out in our licence referred to above.

Other than as permitted in any relevant BMJ Author's Self Archiving Policies, I confirm this Work has not been accepted for publication elsewhere, is not being considered for publication elsewhere and does not duplicate material already published. I confirm all authors consent to publication of this Work and authorise the granting of this licence.

For peer review only

Clinical features and natural history of the first 2,073 suspected COVID-19 cases in the Corona São Caetano primary care programme: a prospective cohort study

Fabio E Leal[1,2]* (PhD), Maria C Mendes-Correa[3]* (PhD), Lewis F Buss[3]*# (MBBS), Silvia F Costa[3] (PhD), Joao CS Bizario[1] (PhD), Sonia RP de Souza[1] (PhD), Osorio Thomaz[4] (PhD), Tania R Tozetto-Mendoza[1] (PhD), Lucy S Villas-Boas[3], Lea CO Silva[3] (PhD), Regina MZ Grespan (MD)[1], Ligia Capuani[5] (PhD), Renata Buccheri[3] (MD), Helves Domingues (PhD)[5], Neal DE Alexander[6] (PhD), Philippe Mayaud[6] (MD), Ester C Sabino[3] (PhD)

* These authors contributed equally

Affiliations

1. Universidade Municipal de São Caetano do Sul (USCS), Rua Santo Antonio, São Caetano do Sul, Brazil
2. National Cancer Institute (INCA), Praça Cruz Vermelha, Rio de Janeiro, Brazil
3. Instituto de Medicina Tropical (LIM-52, LIM-46, LIM-49) and Departamento de Moléstias Infecciosas e Parasitarias da Faculdade de Medicina da Universidade de São Paulo Avenida Dr. Enéas Carvalho de Aguiar, São Paulo, Brazil
4. Instituto de Pesquisas Tecnológicas do Estado de São Paulo - IPT
5. Modular Research System, Avenida Paulista, São Paulo, Brazil
6. London School of Hygiene and Tropical Medicine (LSHTM), Keppel Street, London, United Kingdom

Corresponding author:

Dr Lewis F Buss
Instituto de Medicina Tropical da Faculdade de Medicina da Universidade de São Paulo
lewisbuss@usp.br

KEY WORDS: SARS-CoV-2, COVID-19, pandemic, community, primary care, Brazil

For peer review only

ABSTRACT

Background: Despite most cases not requiring hospital care, there are limited community-based clinical data on COVID-19.

Methods: The Corona São Caetano program is a primary care initiative providing care to all residents with COVID-19 in São Caetano do Sul, Brazil. It was designed to capture standardised clinical data on community COVID-19 cases. After triage of potentially severe cases, consecutive patients presenting to a multimedia screening platform between 13th April and 13th May 2020 were tested at home with SARS-CoV-2 reverse transcriptase (RT) PCR; positive patients were followed up for 14 days with phone calls every 2 days. RT-PCR-negative patients were offered additional SARS-CoV-2 serology testing to establish their infection status. We describe the clinical, virologic and natural history features of this prospective population-based cohort.

Findings: Of 2,073 suspected COVID-19 cases, 1,583 (76.4%) were tested by RT-PCR, of whom 444 (28.0%, 95%CI: 25.9-30.3) were positive; 604/1,136 (53%) RT-PCR-negative patients underwent serology, of whom 52 (8.6%) tested SARS-CoV-2 seropositive. The most common symptoms of confirmed COVID-19 were cough, fatigue, myalgia and headache; whereas self-reported fever (OR 3.0, 95%CI: 2.4-3.9), anosmia (OR 3.3, 95%CI: 2.6-4.4), and ageusia (2.9, 95%CI: 2.3-3.8) were most strongly associated with a positive COVID-19 diagnosis by RT-PCR or serology. RT-PCR cycle thresholds were lower in men, older patients, those with fever and arthralgia, and closer to symptom onset. The rates of hospitalization and death among 444 RT-PCR-positive cases were 6.7% and 0.7%, respectively, with older age and obesity more frequent in the hospitalized group.

Conclusion: COVID-19 presents in a similar way to other mild community-acquired respiratory diseases, but the presence of fever, anosmia, and ageusia can assist the specific diagnosis. Most patients recovered without requiring hospitalization with a low fatality rate compared to other hospital-based studies.

For peer review only - http://bmjopen.bmj.com/site/about/guidelines.xhtml

Strengths and limitations of this study

1. The clinical features of COVID-19 have mostly been described in hospital-based studies which are biased towards severe disease
2. We report a prospective cohort of suspected and confirmed COVID-19 cases from a primary care initiative in the Brazilian municipality of São Caetano do Sul
3. By systematically testing consecutive suspected community cases with molecular and serological tests we were able to address the diagnostic value of clinical features of mild-moderate COVID-19 in primary care
4. Prospective follow-up of confirmed cases and linkage with hospital datasets allowed us to describe the natural history of a primary care COVID-19 population
5. A limitation of the work was that not all PCR-negative participants underwent serology testing due to loss to follow-up

For peer review only

INTRODUCTION

A comprehensive public health response is vital but difficult to achieve during an epidemic. The COVID-19 pandemic, caused by the novel severe acute respiratory syndrome coronavirus 2 (SARS-CoV-2), started in China in late 2019.[1] According to the World Health Organization (WHO)[2,3] and others[4,5], the ideal early response should have been multipronged, with identification, isolation, treatment and contact tracing of symptomatic cases, relying on a strong testing programme. Primary health care is well placed to implement such a response, by identifying cases early and managing them in a way that minimizes overcrowding of emergency rooms and intensive care units.[6,7] Real-time data analysis coming from these primary care response systems can inform policy decisions.

Primary health care (PHC) in Brazil is provided by the publicly funded Unified Health System (SUS – Portuguese acronym) within the family health strategy (*Estratégia Saúde da Família*). Provision of care is centred around a healthcare unit with a multi-professional team that is responsible for all residents in the immediate catchment area[8]. Nearly two-thirds of the Brazilian population is covered by the family health strategy[8].

In Brazil, the first case of COVID-19 was identified in the city of São Paulo on 26th February 2020.[9] As of 15th June 2020 there were 1,400,000 cases nationally, with São Paulo contributing a fifth of these.[10] In March 2020, the Municipal Health Department of the municipality of São Caetano do Sul – part of the Greater Metropolitan Region of São Paulo – began to develop a clinical and testing platform to organize its COVID-19 response. The aim was to provide universal detection and management of symptomatic cases and their contacts. The platform was developed in partnership with two local universities – the Municipal University of São Caetano do Sul (USCS) and the University of Sao Paulo (USP) – and called "Corona São Caetano".

Large scale community-based observational cohorts are difficult to establish under epidemic circumstances, particularly if the risk of exposure for research personnel is high. Hence, most COVID-19 epidemiological and clinical studies have been hospital-based,[11–13] and therefore tend to include more severe cases whose findings may not be generalizable to the general population[14], although some limited descriptions from ambulatory settings are available[15–17].

For peer review only - http://bmjopen.bmj.com/site/about/guidelines.xhtml

The objectives of this study were to describe the epidemiological indicators of the early phase of the programme rollout; and to describe the clinical, virological and natural history features (including hospitalization and deaths) of SARS-CoV-2 infection among patients identified in primary care.

METHODS

Setting

The municipality of São Caetano do Sul has a population of 161,000 inhabitants.[18] The city's population is older than the Brazilian population[18] and its Human Development Index is one of the highest in the country. Nearly all (97.4%) children aged 6-14 are in education and 31% of the population have completed higher education[19] (Brazilian national average is 11%).

Corona São Caetano platform

The objective of the platform was to offer clinical care for patients with flu syndrome and suspected COVID-19. Through the multimedia platform, patients could be triaged and guided in relation to their clinical needs and tested, without having to leave their homes or go to health facilities, unless seriously ill. This strategy aimed at reducing the workload in health units and the risk of SARS-CoV-2 transmission in the population served by these health units. Patients' GPs were informed of lab results and had access to clinical data stored in the platform. GPs were expected to call patients being assisted by the platform and provide medical assistance through home visits or at the primary care clinic if needed. In general, the drugs prescribed through the platform were restricted to analgesics and antipyretics. The platform was designed so that clinical information was collected in a standardized way for research purposes.

Residents of the municipality aged 12 years and older with suspected COVID-19 symptoms were encouraged, through local media reports, to contact the dedicated Corona São Caetano platform via the website (access at https://coronasaocaetano.org/) or by phone. They were invited to complete an initial screening questionnaire that included socio-demographic data; information on symptoms type, onset and duration; and recent contacts.

For peer review only

Patients meeting the suspected COVID-19 case definition (i.e., having at least two of the following symptoms: fever, cough, sore throat, coryza, or change in/loss of smell (anosmia); or one of these symptoms plus at least two other symptoms consistent with COVID-19) were further evaluated, whilst people not meeting these criteria were reassured, advised to stay at home and contact the service again if they were to develop new symptoms or worsening of current ones. The case definition was developed in consultation with infectious disease and primary care specialists to encompass the known symptoms of COVID-19 and is similar to the Brazilian national case definition[20]. Patients were then called by a medical student to complete a risk assessment. All pregnant women, and patients meeting pre-defined triage criteria for severe disease (see Supplemental Material), were advised to attend a hospital service - either an emergency department or outpatient service, depending on availability. All other patients were offered a home visit for self-collection of a nasopharyngeal swab.

Sample collection

Patients self-collected nasopharyngeal swabs (NPS – both nostrils and throat) at their own homes under the supervision of trained healthcare personnel. We sent a link to an instructional video (https://youtu.be/rWZzV2ZP7KY) before the home visit to provide guidance on self-collection procedures. Nasopharyngeal swabs for the molecular detection of SARS-CoV-2 has been recommended as an alternative method of collection for samples from patients with suspected COVID-19[21], as well as other respiratory diseases, and has the advantage of reducing the chance of aerosol transmission to healthcare professionals. Healthcare personnel were instructed to maintain a distance of six feet from the patient and to wear personal protective equipment at all times. Samples were immediately put on a cool box between 2-8°C and stored at 4°C in a fridge until shipment to the lab within 24 hours.

Follow-up procedures

Patients testing SARS-CoV-2 RT-PCR positive were followed up to 14 days[22] (a maximum of 7 phone calls) from completion of their initial questionnaire. They were contacted every 48 hours by a medical student who completed another risk assessment and recorded any ongoing or new symptoms. The purpose of the follow-up was to assess clinical evolution. Where patients were judged to be deteriorating or developing severe disease they were signposted to secondary care services. Patients testing RT-PCR negative were followed up by the primary

For peer review only - http://bmjopen.bmj.com/site/about/guidelines.xhtml

health care program for their residential area. They were advised to contact the platform for a new consultation if they developed new symptoms. Starting on May 19th, when serological testing became available, RT-PCR-negative patients were re-contacted to offer antibody (IgG/IgM combined) testing 14 days after their initial registration as long as they had become asymptomatic.

Study dates

The Corona São Caetano programme was launched on 6th April 2020, with a one week pilot phase designed to test instruments before roll-out. For this analysis, we included all patients making their first contact with the programme in its first month, ie between 13th April and 13th May 2020. The period of follow-up (last date of data extraction) was 4th June 2020, to account for the accrual period (three weeks) of possible hospitalizations in the last included patients.

Laboratory methods

Due to shortages of some reagents, we used two RT-PCR platforms at different times during the study: ALTONA RealStar® SARS-CoV-2 RT-PCR Kit 1.0 (Hamburg, Germany) and the Mico BioMed RT-qPCR kit (Seongnam, South Korea). For serology we tested 10µL of serum or plasma (equivalent in performance) using a qualitative rapid chromatographic immunoassay (Wondfo Biotech Co., Guangzhou, China), that jointly detects anti-SARS-CoV-2 IgG/IgM. The assay has been found to have a sensitivity of 81.5% and specificity of 99.1% in a US study[23]. In our local validation, after two weeks of symptoms, the sensitivity in 59 RT-PCR confirmed cases was 94.9%, and specificity in 106 biobank samples from 2019 was 100%.

Statistical methods

We estimated the contribution of our platform to total number of COVID-19 cases diagnosed in São Caetano do Sul. To do this, we compared the number of cases diagnosed in our programme with official data released by the Municipal Department of Health in its daily bulletins (accessed here https://coronavirus.saocaetanodosul.sp.gov.br).

For peer review only - http://bmjopen.bmj.com/site/about/guidelines.xhtml

Clinical and demographic data were extracted directly from the Corona São Caetano information system. To analyse clinical presentation, we first calculated the proportion and exact binomial 95% confidence intervals (CI) of cases reporting each symptom in the three testing groups: SARS-CoV-2 RT-PCR positive; RT-PCR negative / seropositive; and RT-PCR negative / seronegative. We next combined RT-PCR and serology positive cases to make a confirmed COVID-19 group, and those negative on both tests to make a SARS-CoV-2 negative control group. We express the association between each symptom and a positive COVID-19 diagnosis as odds ratios (OR) and 95% CIs.

Next, we assessed associations between RT-PCR cycle thresholds (Cts) and other clinical features. ALTONA and MiCo BioMed RT-PCR kits each separately amplify two different SARS-CoV-2 viral genes, as such each patient had two Ct values. There was a high concordance between Cts for the two genes within each kit (Figure S1), and we opted therefore to use the mean of the two Ct values for each patient in all analyses. We calculated univariable associations between Cts and age, sex, delay from symptom onset to NPS collection, and presenting symptoms using simple linear regression. We then built a multivariable linear regression model to assess independent associations between presenting symptoms and RT-PCR Cts. As age, sex, and time of swab collection may confound this relationship we included these variables, as well as the RT-PCR platform (ALTONA vs MiCo BioMed), as covariates in the model.

For RT-PCR positive patients, hospitalizations and deaths were extracted from the study platform. To extend the follow-up period and to capture RT-PCR negative patients and those initially triaged to hospital (no study follow-up), hospitalization and vital status was confirmed by linkage with two administrative databases: the municipal epidemiological surveillance dataset, as well as the state-wide influenza-like illness notification system (SIVEP-Gripe). Linkage was last performed on 5th June 2020, 23 days after the last patient was enrolled, by the author SRPS who did not have access to the full analytic dataset. This author searched the SIVEP-Gripe system and the municipal epidemiological surveillance dataset using full name and date of birth. Categorical patient characteristics were compared between patients requiring and those not requiring hospitalization using a Chi-squared or Fisher exact test. Continuous variables were compared using the Wilcoxon rank sum test. A multivariate analysis was not conducted due to the small number of individuals experiencing this outcome.

For peer review only - http://bmjopen.bmj.com/site/about/guidelines.xhtml
For peer review only

The cohort sample included consecutive cases presenting to the Corona São Caetano program and a formal sample size calculation was not performed. Missing data were excluded. All analyses were conducted in R Software for Statistical Computing, version 3.6.3.[24]

Ethics

The study was approved by the local ethics committee (Comissão de Ética para Análise de Projeto de Pesquisa - CAPPesq, protocol No. 13915, dated June 03, 2020). The committee waived the need for informed consent and allowed the development of an analytical dataset with no personal identification for the current analysis.

Patient and public involvement

Patients were not involved in the planning of this research.

RESULTS

Epidemiological and programmatic indicators

Over the study period, there were 2,073 presentations, from 2,011 individual patients, that met the criteria for a suspected COVID-19 case (See Figure 1 for study flow). At initial phone interview, 132 (6%) potential cases were advised to go directly to a health service based on the triage questions, and 12 (0.6%) because of pregnancy. Only four (3%) of referred patients were admitted to hospital and none died.

In total 1,583 individual patients were tested with RT-PCR for SARS-CoV-2; 444 (28.0%, 95%CI: 25.9-30.3) were positive. The proportion of positive results was stable over the study (Figure S2). Among the RT-PCR negative group, 604 (53% of 1,136) underwent serology testing, of whom 52 (8.6%, 95%CI: 6.6-11.1) were seropositive. The median [IQR] time from symptom onset to serology collection was 31 [26–37] days. The age-sex structure of patients being tested differed from the underlying population of São Caetano do Sul (Figure S3) with an overrepresentation of working-age adults and women. At the beginning of programme role out, 25% of notified COVID-19 cases in São Caetano do Sul were diagnosed in our

For peer review only - http://bmjopen.bmj.com/site/about/guidelines.xhtml

programme. Over the study period, adherence to the programme increased, and by May 13th, 2020, this figure had risen to 78%.

Of 444 RT-PCR positive patients eligible for longitudinal follow-up, 326 (73%) had their final follow-up visit at least 14 days after their initial presentation. Of the seven possible follow-up questionnaires, 384 (86%) COVID-19 patients completed three or more, and 162 (36%) completed all seven.

Participant characteristics

Patient characteristics are shown in Table 1. Although women were overrepresented in the cohort, there were proportionally more males in the RT-PCR positive and seropositive groups compared to the seronegative group. Of note, 55% of RT-PCR negative/seronegative patients had completed higher education compared to 35% RT-PCR-positive patients (p <0.001, Chi-squared test). The median number of days from symptom onset to swab collection was 5.0 (IQR, 4.0-7.0) among RT-PCR positive patients and 6.0 (IQR, 4.0-8.3) among RT-PCR negative/seropositive patients (p = 0.06, Wilcoxon rank sum) (Figure S4). Chronic respiratory disease was less frequent in RT-PCR positive than dual-negative patients.

Symptoms of COVID-19

The prevalence of individual symptoms at presentation is shown in Figure 2A stratified by final diagnostic category. The most frequent symptoms among RT-PCR and seropositive patients were headache (82% and 75%), myalgia (80% and 80%), cough (77% and 63%), and fatigue (77% and 79%). Anosmia was present in 56% and 63% of RT-PCR positive and seropositive patients, respectively, compared to 30% in those testing doubly negative. A similar pattern was observed for ageusia (53% and 53% versus 30%). Upper respiratory tract symptoms - including coryza, blocked nose, ageusia, and anosmia - were more frequent in younger people (Figure 2B). The evolution of symptoms over time among RT-PCR positive patients is shown in Figure S5.

The odds ratios for testing positive for SARS-CoV-2 (RT-PCR or serology) associated with each presenting symptom are shown in Figure 3. The symptoms with strongest associations were anosmia (OR 3.3, 95%CI: 2.6-4.4), fever (3.0, 95%CI: 2.4-3.9) and ageusia (2.9,

For peer review only - http://bmjopen.bmj.com/site/about/guidelines.xhtml

95%CI: 2.3-3.8). The presence of sore throat (0.53, 95%CI: 0.41-0.68) and diarrhoea (0.72, 95%CI: 0.55-0.96) were associated with a negative SARS-CoV-2 test.

Associations between SARS-CoV-2 RT-PCR Cycle threshold (Ct) values, and demographic and clinical features

Figure 4 shows the associations between mean RT-PCR cycle threshold and demographic features and symptoms at presentation (the median [IQR] time from presentation to swab was 1 [1-2] day). Older age was associated with lower cycle thresholds, with a change in mean Ct of -0.05 (95%CI -0.09 to -0.01) for each additional year of age. The mean difference in Ct value was -1.36 (95% CI -2.49 to -0.23) in men compared to women. For each doubling in the number of days from symptom onset to swab collection the mean Ct value increased by 3.28 (95%CI 2.33 to 4.03). Presenting symptoms of fever and arthralgia were associated with lower Cts, whereas anosmia, ageusia, vomiting, diarrhoea, and nausea were associated with higher Cts (Figure 4 and Table S1). After adjustment for age, sex, delay from symptom onset, and RT-PCR platform used, fever (-0.06, 95%CI: -2.11 to -0.001) and arthralgia (-1.24, 95%CI: -2.18 to -0.10) remained associated with lower Cts, and anosmia (2.21, 95%CI: 1.0-3.29), ageusia (1.96, 95%CI: 0.88-3.0), and diarrhoea (1.36, 95%CI: 0.12-2.61) with higher Cts (Table S1).

Hospitalizations and deaths

Of the 444 RT-PCR positive patients, 30 (6.8%) had been hospitalized by 5th June 2020, when the database linkage was last updated, and three (0.7%) had died; in-hospital mortality was therefore 10% (3/30). In 28 cases the date of admission was available. The median time from symptom onset to hospital admission was 7 (range 2 to 14) days. Among 1,136 RT-PCR-negative patients, six (0.5%) had been admitted to hospital. One (<0.01% of 1,136) of these six patients died. None of the 604 RT-PCR negative patients that underwent serology were admitted to hospital or died. Table 2 compares patient characteristics by hospitalization status. Notably, hospitalized patients were older, had more cardiovascular comorbidities and were more frequently obese.

DISCUSSION

For peer review only - http://bmjopen.bmj.com/site/about/guidelines.xhtml

We present a community-based cohort of suspected COVID-19 cases recruited through a primary care initiative in the Brazilian municipality of São Caetano do Sul. Offering RT-PCR testing to all patients presenting with symptoms compatible with COVID-19, the positivity rate was 28%, with 8.6% of those testing negative subsequently found to be seropositive - i.e. > 35% of the cohort had a diagnosis of COVID-19. Anosmia, ageusia, and self-reported fever provided the greatest diagnostic value in identifying COVID-19. The rate of hospitalization and deaths among RT-PCR positive patients was low, at 6.8% and 0.7%, respectively. Our results provide important information on the clinical presentation, diagnostic testing and natural history of COVID-19 identified in the community.

The profile of suspected cases that tested positive for COVID-19 differed in some important respects from those testing negative. The lower educational level among positive cases suggests that, in São Caetano do Sul, the risk of exposure to COVID-19 follows a socioeconomic gradient, consistent with other findings from Brazil[25,26]. Although more women presented to the platform, proportionally more men tested positive, consistent with data from São Paulo showing a higher seroprevalence in men than women[27], but also potentially reflecting different health seeking behaviours. Comorbidities were mostly similar, although chronic respiratory disease was less frequent in those testing RT-PCR positive. This may be due to a proportion of presentations in those with chronic respiratory disease being explained by exacerbations of their underlying pathology from aetiologies other than SARS-CoV-2, as well as higher anxiety about COVID-19 in those with pre-existing respiratory disease.

Extrapolating the seropositivity rate among RT-PCR negative patients to the 532 who were not tested with serology, we estimate that an additional 46 seropositive cases would have been identified. As such, 18% (98/542) of COVID-19 cases were missed by RT-PCR in the setting of symptomatic presentations to primary care. This is similar to a pooled analysis showing a false-negative rate for RT-PCR of 20% at three days post-symptom onset.[28] Viral load peaks around the time of symptom onset and remains high over the first symptomatic week (also see Figure 4A).[29,30] Consistent with this, we found a slightly longer delay to swab collection (due to delay in presentation to the platform) in RT-PCR false-negative patients than RT-PCR positive patients (Figure S4).

For peer review only - http://bmjopen.bmj.com/site/about/guidelines.xhtml

COVID-19 presents in a similar way to other respiratory viral illnesses. Indeed, in our cohort the most common symptoms of COVID-19 - such as cough, fatigue, headache, etc. - were reported with a similar frequency among patients testing negative. It is therefore important to have identified anosmia, ageusia, self-reported fever, myalgia, and anorexia as the symptoms with greatest value in the differential diagnosis of COVID-19 in primary care. This is consistent with systematic review evidence highlighting anosmia and ageusia as key diagnostic features of COVID19[31]. It is of note that 30% of jointly RT-PCR and serology negative patients reported these symptoms, indicating that although indicative of COVID-19, the specificity of these symptoms is not high enough to rule in the diagnosis alone. Sore throat and diarrhoea - both considered symptoms of COVID-19 in other settings –[32] were more frequently due to other possible aetiologies in this primary care context.

These results are robust for a number of reasons. Firstly, our sample is representative of the population of interest - i.e. consecutive patients with suspected COVID-19 in the community - instead of extrapolating from hospital cases. Symptom data were collected prospectively, eliminating recall or interviewer bias. Finally, we have a control group of patients who were negative for both RT-PCR and serology, minimizing misclassification due to false negative RT-PCR.

In our study, the proportion of patients with a positive SARS-CoV-2 RT-PCR requiring hospitalization was low (7%). Early reports from China were of 13.8% of cases being severe[33], but this value was lower when under ascertainment of cases was accounted for.[34,35] This is because our cohort reflects mild to moderate cases, as severely ill patients are likely to have attended hospital directly. As such, only 3% of patients we triaged to attend health services were ultimately hospitalized, possibly due to self-selection of patients presenting to our service. Supporting this, our overall case fatality ratio among RT-PCR positive patients was 0.7%. The rate of hospitalization was lower (0.5%) in those testing PCR-negative. These patients were admitted with a severe acute respiratory syndrome of an aetiology other than SARS-CoV-2. The 14-fold higher admission rate among PCR-positive cases highlights the importance of molecular testing for SARS-CoV-2 in patients presenting with features of respiratory viral illness to primary care.

As expected, the main determinant of Ct was the delay between symptom onset and swab collection, mostly due to the delay in reporting to the platform. After adjusting for this, as

For peer review only - http://bmjopen.bmj.com/site/about/guidelines.xhtml

well as age and sex, we found that a self-reported fever and arthralgia were associated with lower Cts. The presence of these symptoms may identify patients with a higher viral load in the community. However, these results should be seen as purely exploratory, and the wide spread of Ct values around the regression line precludes a direct clinical application at present.

Our study has some limitations. Firstly, serology was not performed on all RT-PCR negative patients due to on-going symptoms, loss to follow-up, or patient refusal. Of note, none of the RT-PCR-negative patients that were admitted to hospital underwent serology testing. This suggests that patients who were not tested with serology may have had a higher prevalence of COVID-19 than those that were tested. In addition, imperfect serology test performance (81% sensitivity)[23] will introduced false-negative results. Taken together, these biases may have underestimated the true seroprevalence among RT-PCR-negative cases, as well as the false-negative rate of RT-PCR. The latter calculation may also have been influenced by the inclusion of RT-PCR positive patients in the denominator, introducing an incorporation bias.[36] Furthermore, the association between symptoms and COVID-19 diagnosis was based on the comparison with doubly PCR and serology negative individuals. It is not clear how the exclusion of individuals that did not undergo serology testing would have influenced these associations. Finally, patients were not involved in the planning of the Corona platform or the research proposal.

A key strength to our study relates to the provision of primary healthcare in Brazil and its symbiosis with medical training nationwide. Primary health care - within the family health strategy (*Estratégia Saúde da Família*) - is centered around a healthcare unit with a multi-professional team that is responsible for all residents in the immediate catchment area [8]. São Caetano do Sul has enough GP units within the family health strategy that all residents have access to primary care. Medical students from the municipal university (USCS) are integrated into the primary healthcare teams and progressively trained from the first year of medical school. Our initiative took advantage of this existing system, with the addition of an online platform allowing remote clinical assessment and follow-up. The suspension of normal clinical training at the medical school provided the workforce. The partnership with the University of São Paulo, which provided the laboratory diagnostics, created the unique opportunity to establish our prospective community cohort of suspected and confirmed COVID-19 cases. But we believe that this infrastructure could be implemented in other

For peer review only - http://bmjopen.bmj.com/site/about/guidelines.xhtml

regions with less resources. Other respiratory disease such as influenza, measles, or tuberculosis may benefit from similar approach. However, further evaluation of the impact of the Corona Platform are required.

CONCLUSION

Systematic testing of all suspected COVID-19 cases was feasible within primary care services in a Brazilian municipality. Anosmia, agueusia, and fever provide the greatest diagnostic discrimination from other similar primary care presentations. Home-care is a valid approach for most for most of these patients with a low rate of hospitalization and death. Our programme model – integrating multimedia technology, telehealth with universal access to primary care – may be successful in other contexts.

CONTRIBUTION STATEMENT

FEL, MCMC, SFC, MC, RB, and ECS conceived and designed the study. FEL, RMZG, and JCSB provided clinical oversight and supervision of medical students. FEL, MCMC, LFB, HD, OT, LC, and SRPS collected and curated the data. MCMC, TRTM, LSVB, and LCOS performed the laboratory analysis. LFB performed the formal statistical analysis with assistance from FEL, SRPS, NDEA, PM, ECS and OT. LFB, FEL, PM and ECS wrote the first draft, and all authors reviewed, contributed to and approved the final version.

CONFLICTS OF INTEREST STATEMENT

The authors have no conflicts of interests. FL, RG, and JB were involved in providing clinical care within the Corona São Caetano Platform.

FUNDING STATEMENT

The municipal health department of São Caetano do Sul (Secretaria Municipal de Saúde da Prefeitura de São Caetano do Sul) funded the establishment and implementation of the platform. We also acknowledge an award from FAPESP (2018/14389-0) and the UK Medical Research Council (MR/S0195/1) to the Brazil-UK Centre for Arbovirus Discovery, Diagnosis, Genomics and Epidemiology (CADDE).

For peer review only - http://bmjopen.bmj.com/site/about/guidelines.xhtml

For peer review only

DATA SHARING STATEMENT

Data will be made available via the linked Figshare repository (URL) upon acceptance of the manuscript.

REFERENCES

1. World Health Organization. (2020). Pneumonia of unknown cause – China. (https://www.who.int/csr/don/05-january-2020-pneumonia-of-unkown-cause-china/en/, accessed 2020-06-18).
2. World Health Organization. Critical preparedness, readiness and response actions for COVID-19 (Interim Guidance) (https://www.who.int/publications-detail/critical-preparedness-readiness-and-response-actions-for-covid-19, accessed 15 June 2020).
3. Bedford, J. et al. COVID-19: towards controlling of a pandemic. The Lancet **395**, 1015–1018 (2020).
4. Hellewell, J. et al. Feasibility of controlling COVID-19 outbreaks by isolation of cases and contacts. Lancet Glob Health **8**, e488–e496 (2020).
5. Dunlop, C., Howe, A., Li, D. & Allen, L. N. The coronavirus outbreak: the central role of primary care in emergency preparedness and response. BJGP Open **4**, bjgpopen20X101041 (2020).
6. World Health Organization. Regional Office for the Western Pacific. (2020). Role of primary care in the COVID-19 response. (https://apps.who.int/iris/handle/10665/331921, accessed 15 June, 2020).

For peer review only

7. Redwood-Campbell, L. & Abrahams, J. Primary Health care and Disasters—The Current State of the Literature: What We Know, Gaps and Next Steps. Prehosp. Disaster med. **26**, 184–191 (2011).

8. Macinko, J. & Harris, M. J. Brazil's Family Health Strategy — Delivering Community-Based Primary Care in a Universal Health System. NEJM **372**, 2177–2181 (2015).

9. World Health Organization. Situation report 27 (2020). (https://www.who.int/emergencies/diseases/novel-coronavirus-2019/situation-reports, accessed 15 June 2020).

10. Brazilian Ministry of Health. (https://covid.saude.gov.br/).

11. Docherty, A. B. et al. Features of 20 133 UK patients in hospital with covid-19 using the ISARIC WHO Clinical Characterisation Protocol: prospective observational cohort study. BMJ m1985 (2020) doi:10.1136/bmj.m1985.

12. Richardson, S. et al. Presenting Characteristics, Comorbidities, and Outcomes Among 5700 Patients Hospitalized With COVID-19 in the New York City Area. JAMA **323**, 2052 (2020).

13. Zhou, F. et al. Clinical course and risk factors for mortality of adult inpatients with COVID-19 in Wuhan, China: a retrospective cohort study. The Lancet **395**, 1054–1062 (2020).

14. Rauh, A. L. & Linder, J. A. Covid-19 care before, during, and beyond the hospital. BMJ m2035 (2020) doi:10.1136/bmj.m2035.

For peer review only

15. Lapostolle, F. et al. Clinical features of 1487 COVID-19 patients with outpatient management in the Greater Paris: the COVID-call study. Intern Emerg Med **15**, 813–817 (2020).

16. Mizrahi, B. et al. Longitudinal symptom dynamics of COVID-19 infection in primary care. http://medrxiv.org/lookup/doi/10.1101/2020.07.13.20151795 (2020) doi:10.1101/2020.07.13.20151795.

17. Williamson, E. J. et al. Factors associated with COVID-19-related death using OpenSAFELY. Nature **584**, 430–436 (2020).

18. Instituto Brasileiro de Geografia e Estatística (IBGE). (2020). São Caetano do Sul / Panorama. (https://cidades.ibge.gov.br/brasil/sp/sao-caetano-do-sul/panorama).

19. Human Development Atlas Brazil. (2020). São Caetano do Sul (http://atlasbrasil.org.br/2013/pt/perfil_m/sao-caetano-do-sul_sp).

20. Brazilian COVID-19 case definition and notification criteria. Brazilian Ministry of Health (https://coronavirus.saude.gov.br/definicao-de-caso-e-notificacao).

21. Akmatov, M. K., Gatzemeier, A., Schughart, K. & Pessler, F. Equivalence of Self- and Staff-Collected Nasal Swabs for the Detection of Viral Respiratory Pathogens. PLoS ONE **7**, e48508 (2012).

22. Walsh, K. A. et al. SARS-CoV-2 detection, viral load and infectivity over the course of an infection. Journal of Infection **81**, 357–371 (2020).

23. Whitman, J. D. et al. Test performance evaluation of SARS-CoV-2 serological assays. medRxiv 2020.04.25.20074856 (2020) doi:10.1101/2020.04.25.20074856.

For peer review only

24. R Core Team. R: A language and environment for statistical computing. R Foundation for Statistical Computing, Vienna, Austria. URL https://www.R-project.org/. (2019).

25. Baqui, P., Bica, I., Marra, V., Ercole, A. & van der Schaar, M. Ethnic and regional variations in hospital mortality from COVID-19 in Brazil: a cross-sectional observational study. The Lancet Global Health **8**, e1018–e1026 (2020).

26. Hallal, P. C. et al. SARS-CoV-2 antibody prevalence in Brazil: results from two successive nationwide serological household surveys. The Lancet Global Health S2214109X20303879 (2020) doi:10.1016/S2214-109X(20)30387-9.

27. Buss, L. F. et al. COVID-19 herd immunity in the Brazilian Amazon. http://medrxiv.org/lookup/doi/10.1101/2020.09.16.20194787 (2020) doi:10.1101/2020.09.16.20194787.

28. Kucirka, L. M., Lauer, S. A., Laeyendecker, O., Boon, D. & Lessler, J. Variation in False-Negative Rate of Reverse Transcriptase Polymerase Chain Reaction–Based SARS-CoV-2 Tests by Time Since Exposure. Ann Int Med M20-1495 (2020) doi:10.7326/M20-1495.

29. To, K. K.-W. et al. Temporal profiles of viral load in posterior oropharyngeal saliva samples and serum antibody responses during infection by SARS-CoV-2: an observational cohort study. Lancet Inf Dis **20**, 565–574 (2020).

30. He, X. et al. Temporal dynamics in viral shedding and transmissibility of COVID-19. Nat Med **26**, 672–675 (2020).

31. Carpenter, C. R., Mudd, P. A., West, C. P., Wilber, E. & Wilber, S. T. Diagnosing COVID-19 in the Emergency Department: A Scoping Review of Clinical

For peer review only

Examinations, Laboratory Tests, Imaging Accuracy, and Biases. Acad Emerg Med **27**, 653–670 (2020).

32. Vetter, P. et al. Clinical features of covid-19. BMJ m1470 (2020) doi:10.1136/bmj.m1470.
33. T. Team, Vital Surveillances: The Epidemiological Characteristics of an Outbreak of 2019 Novel Coronavirus Diseases (COVID-19)-China. China CDC Weekly **2**, 113–122 (2020).
34. Salje, H. et al. Estimating the burden of SARS-CoV-2 in France. Science eabc3517 (2020) doi:10.1126/science.abc3517.
35. Verity, R. et al. Estimates of the severity of coronavirus disease 2019: a model-based analysis. Lancet Infect Dis **20**, 669–677 (2020).
36. Worster, A. & Carpenter, C. Incorporation bias in studies of diagnostic tests: how to avoid being biased about bias. CJEM **10**, 174–175 (2008).

For peer review only

TABLE LEGENDS

Table 1 Demographic and clinical characteristics of 1,048 suspected COVID-19 cases undergoing diagnostic testing in the Corona São Caetano program. * Security, emergency services, supermarket, public transport, and pharmacy workers. IQR: interquartile range; HCW: health care workers, COPD: chronic obstructive pulmonary disease. Missing data – educational level 2; essential occupation 2; body mass index 4; cardiovascular disease 28; diabetes 31 mellitus; chronic resp. disease 65; chronic kidney disease 27; COPD 28. P-values calculated by Chi-squared, Fisher exact, or Wilcoxon rank sum.

Table 2 Characteristics of RT-PCR positive patients stratified by hospitalization status. Missing data – body mass index 2; cardiovascular disease 12; diabetes mellitus 12; chronic respiratory disease 29; COPD 11; chronic kidney disease 12; COPD - chronic obstructive pulmonary disease; IQR - interquartile range.

FIGURE LEGENDS

Figure 1 Patient flowchart for the Corona São Caetano platform between 13th April and 13th May 2020. In the upper section (white background) the numbers correspond to individual presentations to the system; among suspected cases 2,073 suspected cases, 60 had two presentations and one had three. In the lower section (grey background) numbers correspond to individual patients making up the final analytic groups.

Figure 2 Panel A presents prevalence (point) and exact binomial 95% confidence intervals (vertical lines) of symptoms at presentation among patients with suspected COVID-19 according to RT-PCR result and serostatus (A). Panels B and C present the prevalence of presenting symptoms among patients with COVID-19 (RT-PCR and serology positive) stratified by age (B) and sex (C).

Figure 3 Odds ratios (black dot) and 95% confidence intervals (lines) for testing positive for COVID-19 (RT-PCR positive or serology positive) associated with the presence of each presenting symptom. Horizontal axis is on log scale. Point estimates of odds ratios are shown inline with their corresponding symptom.

For peer review only

Figure 4 Relationship between mean RT-PCR cycle threshold (Ct) and day of illness course when the nasopharyngeal swab was collected (A), patient age (B), patient sex (C), and different symptoms at presentation. Panels A and B show the best fit linear regression lines, panels C and D are violin plots (rotated kernel density plots showing the full distribution of data) of the Ct values with median (black dot) and interquartile range (black line).

For peer review only

For peer review only - http://bmjopen.bmj.com/site/about/guidelines.xhtml

Table 1

	**RT-PCR +ve (G1)** N = 444 n (%) or median (IQR)	**RT-PCR -ve Sero +ve (G2)** N=52 n (%) or median (IQR)	**RT-PCR -ve Sero -ve (G3)** N = 552 n (%) or median (IQR)	**p-value** G1 versus G2	**p-value** G1 versus G3
**Sex**					
Male	200 (45·0)	23 (44·2)	185 (33·5)		
Female	244 (55·0)	29 (55·8)	367 (66·5)	1·0	<0·001
**Age groups (years)**					
10 to 19	29 (6·5)	1 (1·9)	25 (4·5)		
20 to 39	197 (44·4)	17 (32·7)	236 (42·8)		
40 to 59	158 (35·6)	28 (53·8)	218 (39·5)		
60+	60 (13·5)	6 (11·5)	73 (13·2)	0·07	0·40
**Educational level**					
Up to primary education	75 (16·9)	7 (13·5)	56 (10·2)		
High school	214 (48·3)	19 (36·5)	194 (35·2)		
University	154 (34·8)	26 (50·0)	301 (54·6)	0·10	<0·001
**Essential Occupation**					
Non-HCW essential job *	137 (30·9)	12 (23·1)	148 (26·9)		
Carers	10 (2·3)	0 (0·0)	8 (1·5)		
HCW	32 (7·2)	5 (9·6)	73 (13·2)		
No	264 (59·6)	35 (67·3)	322 (58·4)	0·45	0·01
**Body mass index (kg/m^2)**					
<25	151 (34·2)	22 (42·3)	211 (38·4)		
25-29	182 (41·2)	17 (32·7)	187 (34·0)		
30-35	79 (17·9)	9 (17·3)	112 (20·4)		
35+	30 (6·8)	4 (7·7)	40 (7·3)	0·62	0·14
**Comorbidities**					
Cardiovascular disease	88 (20·4)	9 (17·6)	129 (24·0)	0·89	0·40
Diabetes mellitus	48 (11·1)	4 (7·8)	39 (7·3)	0·86	0·12
Any chronic resp. disease	37 (8·9)	9 (18·0)	79 (15·3)	0·13	0·01
COPD	24 (5·5)	5 (9·8)	54 (10·1)	0·47	0·03
Chronic kidney disease	1 (<1)	0 (0·0)	3 (1·0)	1·0	0·83
**Time from symptom onset to swab collection (days)**, median (IQR)	5·0 (4·0-7·0)	6·0 (4·0-8·3)	6·0 (4·0-9·0)	0·06	<0·001

For peer review only - http://bmjopen.bmj.com/site/about/guidelines.xhtml

Table 2

	Hospitalized n=30 n (%) or median (IQR)	Not hospitalized n=414 n (%) or median (IQR)	p-value
**Age (years)**			
10 to 19	1 (3)	28 (97)	
20 to 39	6 (3)	191 (97)	
40 to 59	14 (9)	144 (91)	
60+	9 (15)	51 (85)	0·006
**Sex**			
Female	16 (7)	228 (93)	
Male	14 (7)	186 (93)	0·852
**Comorbidities**			
Cardiovascular disease	11 (13)	77 (87)	0·001
Diabetes mellitus	8 (17)	40 (83)	0·007
Any chronic resp. disease	2 (5)	35 (95)	1·0
COPD	1 (5)	23 (95)	1·0
Chronic kidney disease	1 (100)	0 (0)	0·06
**Body mass index (Kg/m^2)**			
<25	4 (3)	147 (97)	
25-29	8 (4)	174 (96)	
30-35	12 (15)	67 (85)	
35+	6 (20)	24 (80)	<0·001
**Time to presentation (days)**	3 (3 to 4)	4 (3 to 5)	0·072

For peer review only

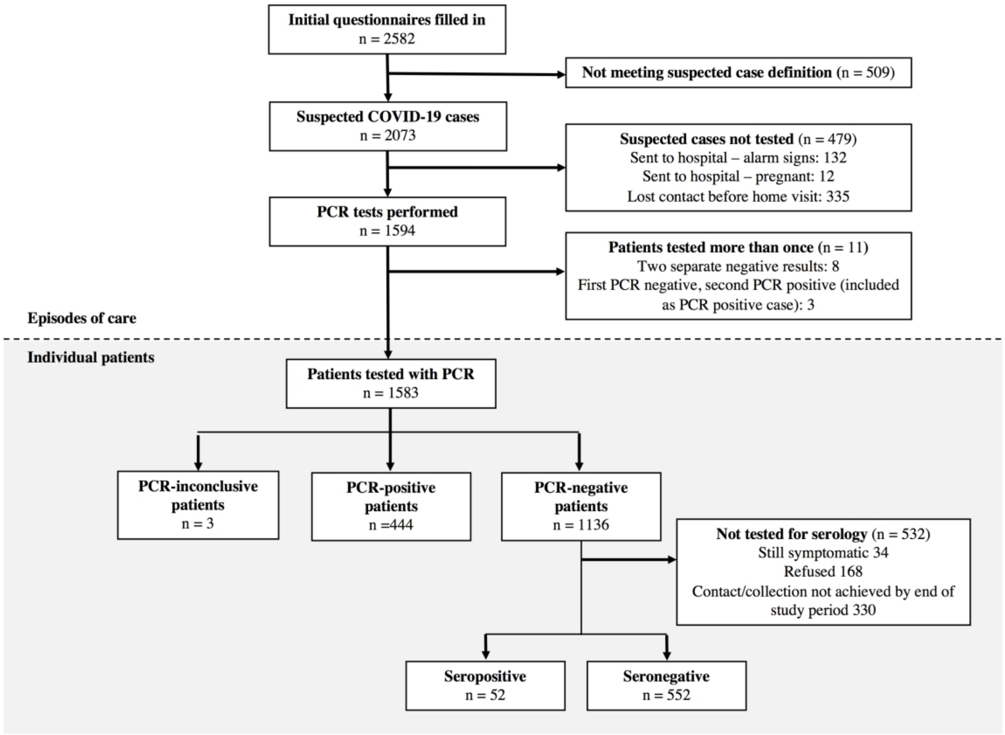

Figure 1

246x181mm (300 x 300 DPI)

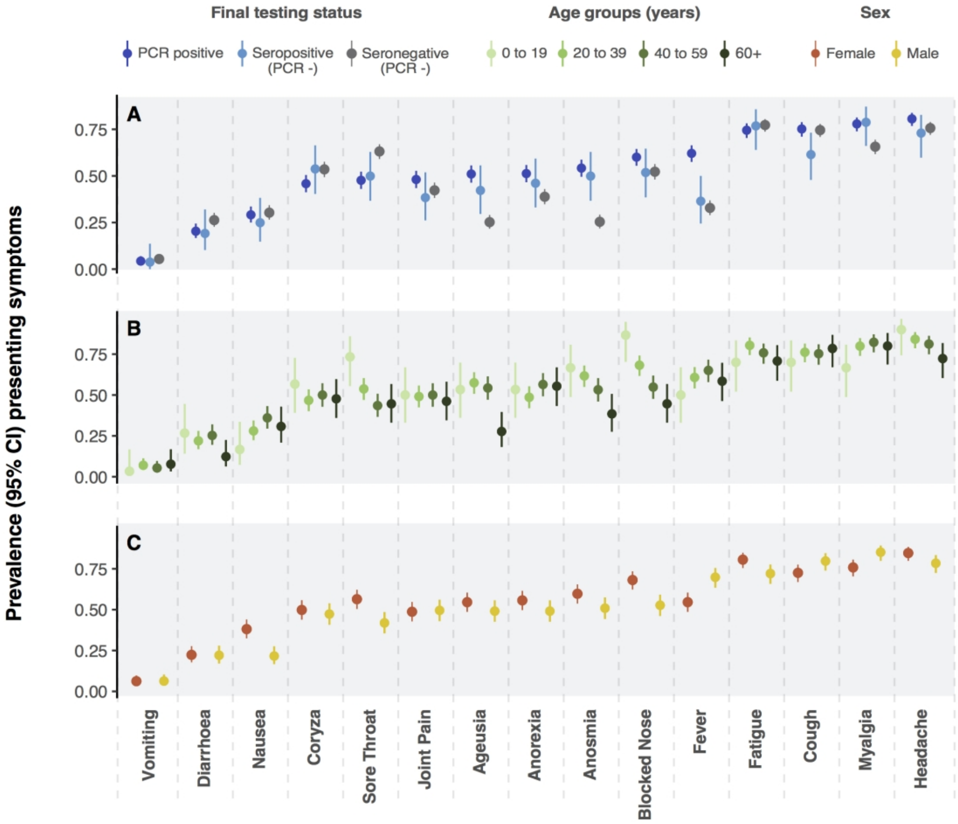

Figure 2

187x158mm (300 x 300 DPI)

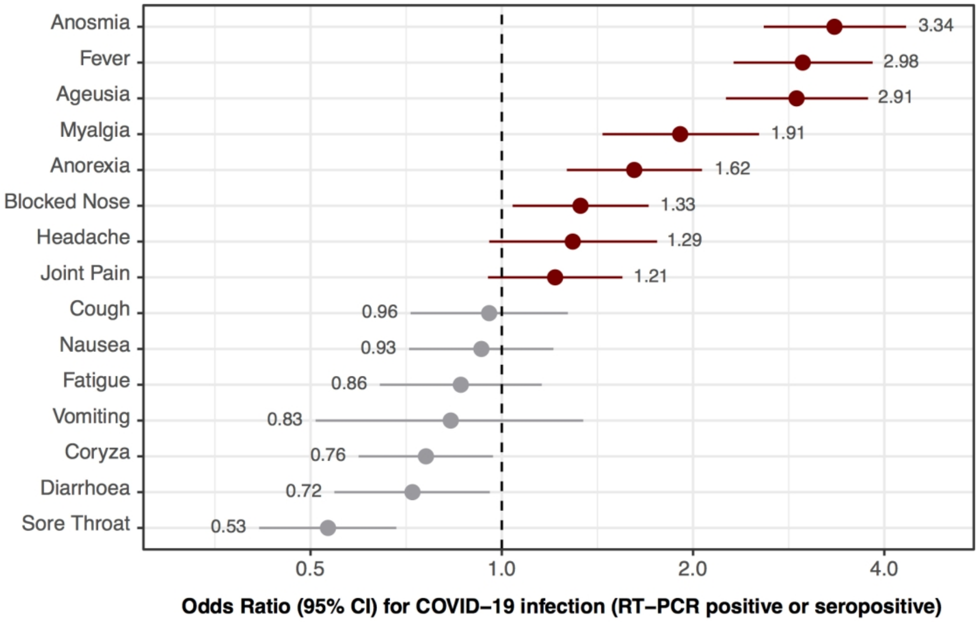

Figure 3

154x100mm (300 x 300 DPI)

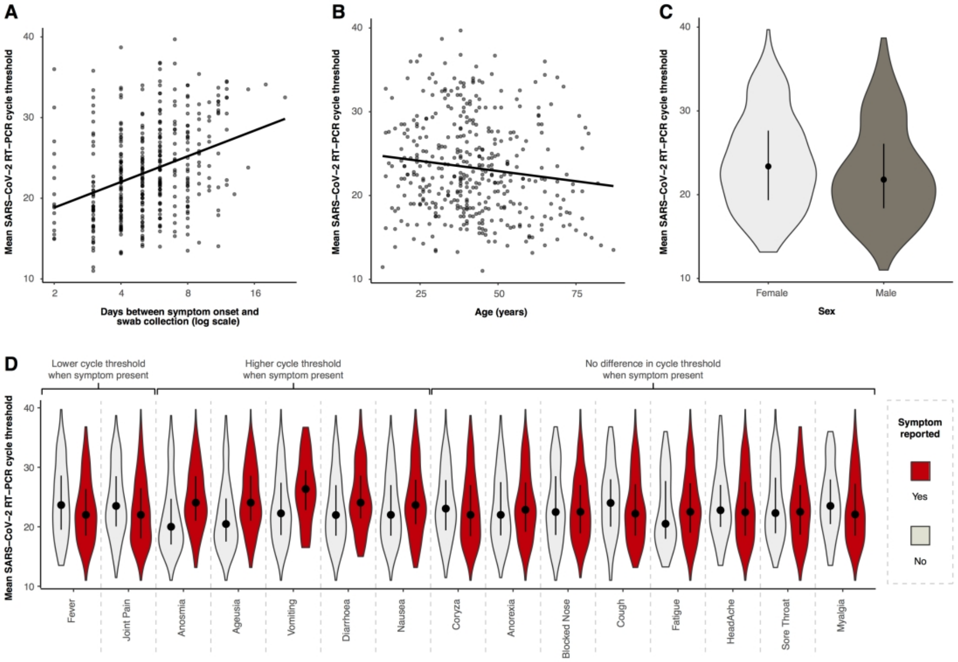

Figure 4

327x222mm (300 x 300 DPI)

Supplemental material

Initial risk assessment

Patients meeting the definition of a suspected case were called by a medical student (under supervision) to complete a risk assessment. All patients were asked a set of standardized questions:

- Do you feel short of breath?
- Are you breathing quickly or finding it difficult to breath?
- If yes, can you count your respiratory rate over one minute? (respiratory rate >20 breaths/minute was considered tachypnoea)
- Has your fever worsened over the last 3 days or have you had a new fever after 2 days being fever-free?
- Have you felt confused or lethargic?

If the patient answered "yes" to any of these questions they were advised to attend a specialist health service. Among the 132 patients that were triaged to hospital, 76 (58% of 132) had shortness of breath, 76 (58% of 132) reported rapid breathing, 33 (25% of 132) persistent fever and 22 (17% of 132) altered mental status.

For peer review only

Table S1 Univariable and adjusted associations between RT-PCR cycle thresholds and patient characteristics

	**Unadjusted analysis**		**Adjusted analysis ***	
	**Beta** (difference in means)	**95% Confidence interval**	**Beta** (difference in means)	**95% Confidence interval**
**Age** (years)	**-0.05**	**-0.09 to -0.01**	**-0.06**	**-0.09 to -0.03**
**Male sex**	**-1.36**	**-2.49 to -0.23**	-1.05	-2.09 to <0.001
**Days from symptom onset to swab collection** (days, log_2)	**3.28**	**2.33 to 4.03**	**3.27**	**0.42 to 7.70**
**PCR platform** (ALTONA as reference)	**-1.19**	**-2.37 to -0.02**	**-1.53**	**-2.6 to -0.45**
**Symptoms at presentation**				
Fever	**-1.78**	**-2.96 to -0.59**	**-1.11**	**-2.11 to -0.001**
Myalgia	-1.31	-2.75 to 0.12	-0.78	-2.11 to 0.53
Arthralgia	**-1.64**	**-2.77 to -0.52**	**-1.24**	**-2.18 to -0.10**
Anosmia	**3.15**	**2.04 to 4.25**	**2.21**	**1.0 to 3.29**
Agusia	**2.99**	**1.89 to 4.09**	**1.96**	**0.88 to 3.0**
Diarrhea	**2.19**	**0.84 to 3.53**	**1.36**	**0.12 to 2.61**
Nausea	**1.50**	**0.28 to 2.72**	1.09	-0.04 to 2.24
Vomiting	**2.99**	**0.52 to 5.46**	2.02	-0.28 to 4.33
Anorexia	0.56	-0.57 to 1.70	0.47	-0.58 to 1.51
Headache	-0.58	-2.12 to 0.97	-0.81	-2.25 to 0.63
Fatigue	0.84	-0.50 to 2.18	0.34	-0.91 to 1.59
Coryza	-0.78	-1.92 to 0.34	-0.68	-1.72 to 0.34
Blocked nose	-0.36	-1.53 to 0.81	**-1.48**	**-2.59 to -0.37**
Cough	-1.33	-2.70 to 0.03	**-1.60**	**-2.86 to -0.33**
Sore throat	-0.49	-1.62 to 0.64	-0.45	-1.52 to 0.61

* All variables adjusted for age (continuous in years), sex (female as reference group), PCR platform (ALTONA platform as the reference group) and time between symptom onset and swab collection (log base 2). Analysis was performed within a linear regression framework. Positive beta coefficients indicate higher cycle thresholds (lower viral load) associated with that variable, whereas negative beta coefficients indicate lower cycle thresholds when the variable is present. Results in bold reached statistical significance.

For peer review only

Supplemental figures

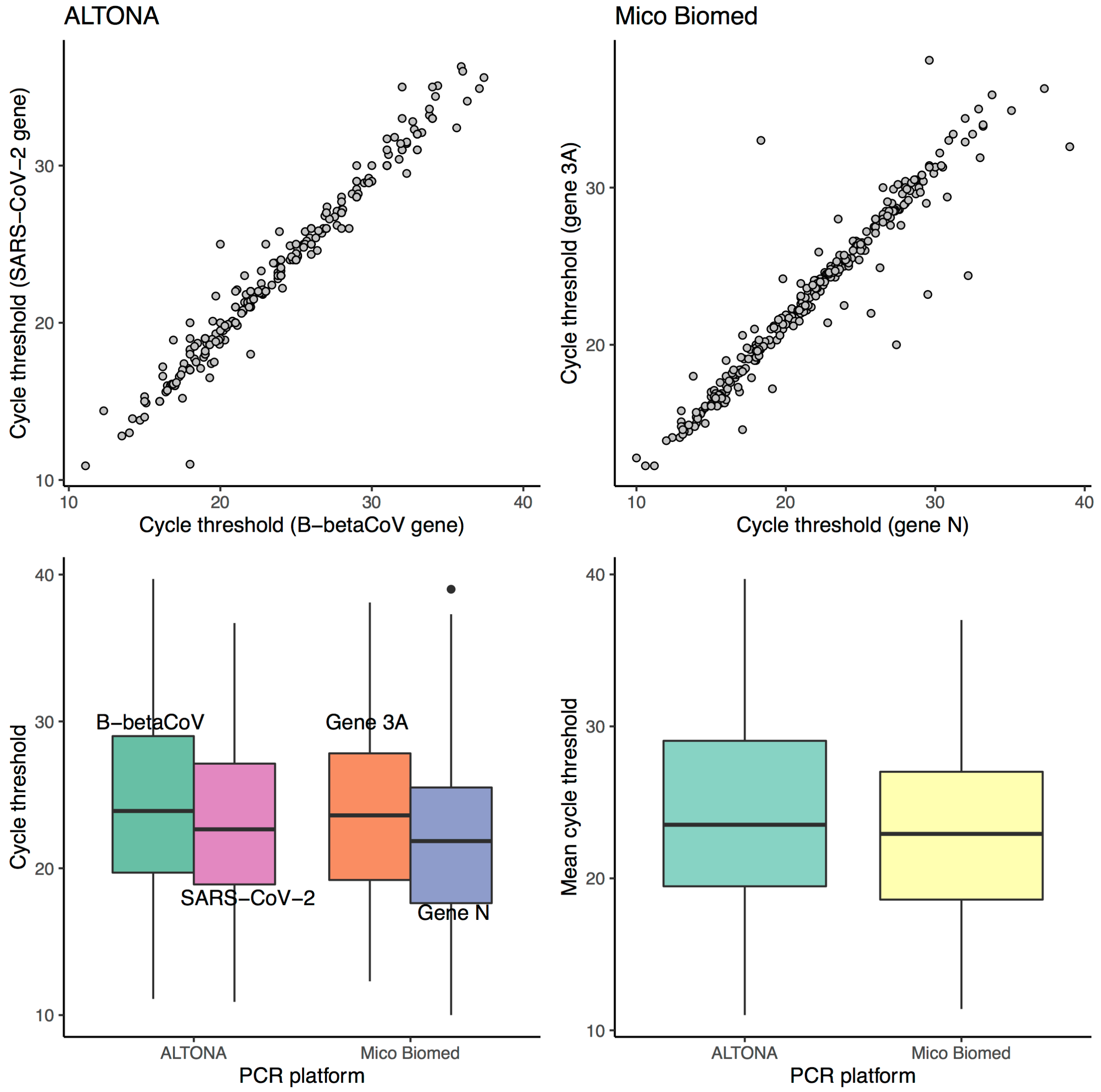

Figure S1 Comparison of cycle thresholds across PCR platforms and genes amplified. Upper two panels show the concordance between cycle thresholds for the two separate genes amplified by the ALTONA (left) and Mico Biomed (right) kits. Lower left panel – distribution of cycle thresholds by gene amplified and RT-PCR platform used. Lower right-hand panel – distribution of the mean cycle threshold (mean of cycle thresholds for separate genes) between different RT-PCR platforms.

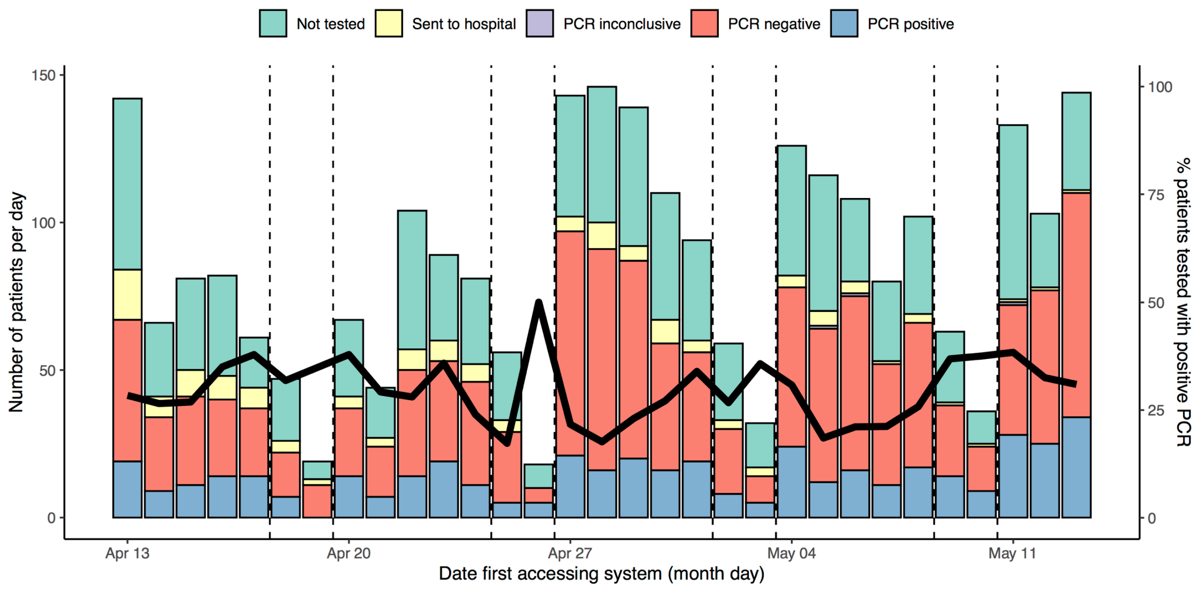

Figure S2 Time series of presentations to the Corona São Caetano platform. Dashed vertical lines denote the weekends with a reduced number of presentations. Thick black line corresponds to the right-hand y-axis: proportion of RT-PCRs performed with positive result.

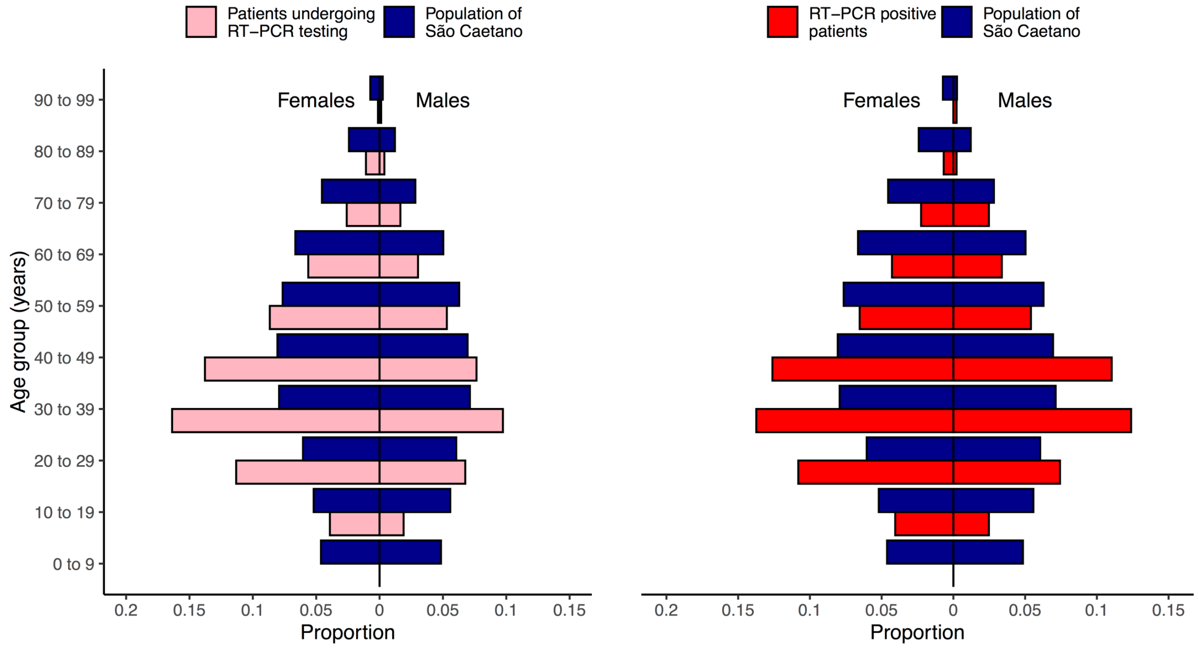

Figure S3 Age-sex distribution the city of São Caetano do Sul compared with that of patients accessing the Corona São Caetano system and being tested with RT-PCR (left-hand panel) and those testing positive for SARS-CoV-2 (right-hand panel).

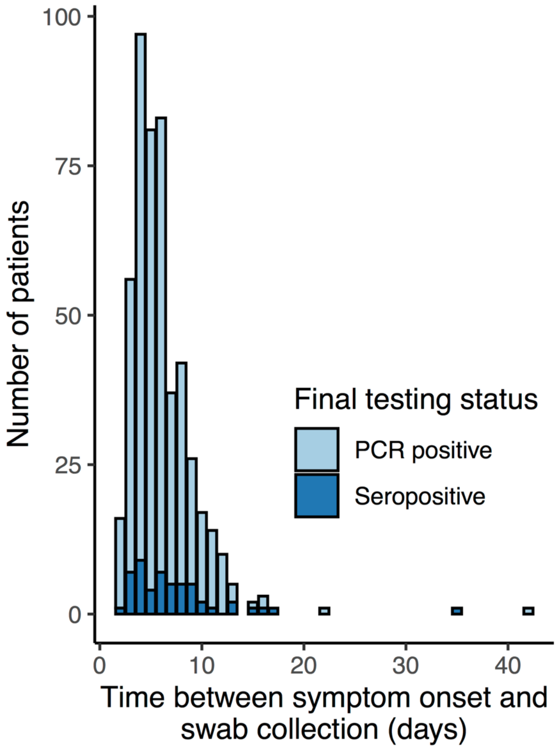

Figure S4 Histogram of delay between symptom onset and swab collection among patients with COVID-19.

For peer review only

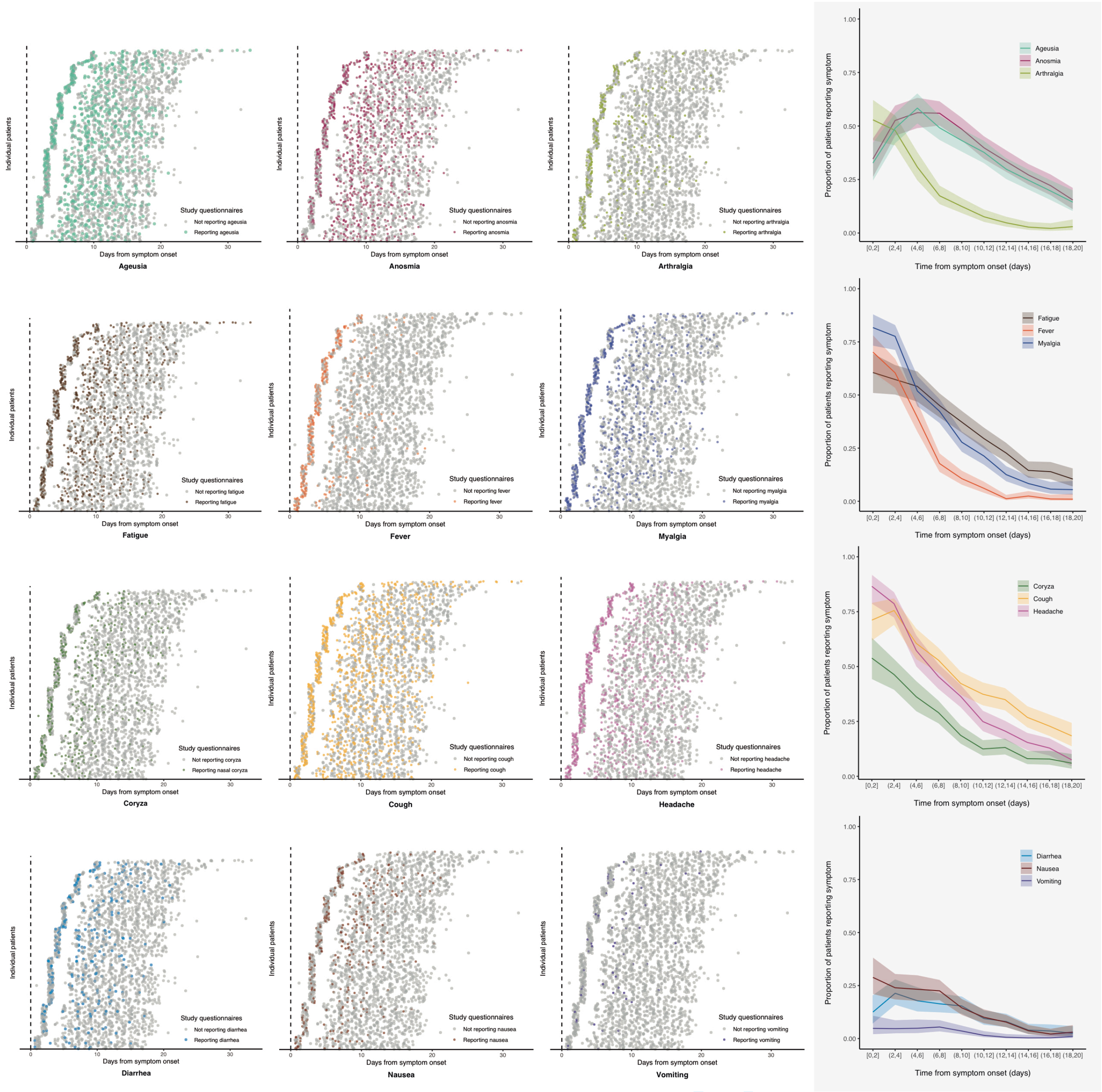

Figure S5 Left hand figures show symptoms at each follow-up questionnaire among patients testing RT-PCR positive and undergoing follow-up. Individual patients are stacked on the y-axis ordered according to the delay from symptom onset to presentation. Each point represents the response to a questionnaire and its position on the horizontal axis the time after symptom onset that the questionnaire was filled in. Grey points are questionnaires where the patient denied the presence of a given symptom. The coloured points correspond to questionnaires in which the patient reported a given symptom. The right-hand figures results from grouping the horizontal axis time into two-day windows and calculating the proportion of completed questionnaires in which each symptom was reported. The denominators for the horizontal axis groups (number of questionnaires completed within a given time window from symptom onset) are 104 at [0-2] days, 192 at (2-4], 185 at (4-6], 293 at (6-8], 338 at (8-10], 329 at (10-12], 335 at (12-14], 324 at (14-16], 280 at (16-18] and 201 at (18-20].

STROBE Statement—Checklist of items that should be included in reports of ***cohort studies***

	Item No	Recommendation	Page No
**Title and abstract**	1	(*a*) Indicate the study's design with a commonly used term in the title or the abstract<br>(*b*) Provide in the abstract an informative and balanced summary of what was done and what was found	1 and 2
**Introduction**			
Background/rationale	2	Explain the scientific background and rationale for the investigation being reported	4
Objectives	3	State specific objectives, including any prespecified hypotheses	4
**Methods**			
Study design	4	Present key elements of study design early in the paper	5 to 7
Setting	5	Describe the setting, locations, and relevant dates, including periods of recruitment, exposure, follow-up, and data collection	5 to 6
Participants	6	(*a*) Give the eligibility criteria, and the sources and methods of selection of participants. Describe methods of follow-up<br>(*b*) For matched studies, give matching criteria and number of exposed and unexposed	A – 5<br>B - NA
Variables	7	Clearly define all outcomes, exposures, predictors, potential confounders, and effect modifiers. Give diagnostic criteria, if applicable	7 to 8
Data sources/ measurement	8*	For each variable of interest, give sources of data and details of methods of assessment (measurement). Describe comparability of assessment methods if there is more than one group	5 to 7
Bias	9	Describe any efforts to address potential sources of bias	7 to 8
Study size	10	Explain how the study size was arrived at	8
Quantitative variables	11	Explain how quantitative variables were handled in the analyses. If applicable, describe which groupings were chosen and why	
Statistical methods	12	(*a*) Describe all statistical methods, including those used to control for confounding<br>(*b*) Describe any methods used to examine subgroups and interactions<br>(*c*) Explain how missing data were addressed<br>(*d*) If applicable, explain how loss to follow-up was addressed<br>(*e*) Describe any sensitivity analyses	A – 7 to 8<br>B – NA<br>C – 8<br>D – NA<br>E – NA
**Results**			
Participants	13*	(a) Report numbers of individuals at each stage of study—eg numbers potentially eligible, examined for eligibility, confirmed eligible, included in the study, completing follow-up, and analysed<br>(b) Give reasons for non-participation at each stage<br>(c) Consider use of a flow diagram	Figure 1 and page 9
Descriptive data	14*	(a) Give characteristics of study participants (eg demographic, clinical, social) and information on exposures and potential confounders<br>(b) Indicate number of participants with missing data for each variable of interest	A – table 1 and pages 9to10<br>B - Table 1 and 2 legends

For peer review only

		(c) Summarise follow-up time (eg, average and total amount)	C – page 9
Outcome data	15*	Report numbers of outcome events or summary measures over time	11
Main results	16	(*a*) Give unadjusted estimates and, if applicable, confounder-adjusted estimates and their precision (eg, 95% confidence interval). Make clear which confounders were adjusted for and why they were included<br>(*b*) Report category boundaries when continuous variables were categorized<br>(*c*) If relevant, consider translating estimates of relative risk into absolute risk for a meaningful time period	Page 7 and results section
Other analyses	17	Report other analyses done—eg analyses of subgroups and interactions, and sensitivity analyses	NA
**Discussion**			
Key results	18	Summarise key results with reference to study objectives	12
Limitations	19	Discuss limitations of the study, taking into account sources of potential bias or imprecision. Discuss both direction and magnitude of any potential bias	13
Interpretation	20	Give a cautious overall interpretation of results considering objectives, limitations, multiplicity of analyses, results from similar studies, and other relevant evidence	13
Generalisability	21	Discuss the generalisability (external validity) of the study results	13
**Other information**			
Funding	22	Give the source of funding and the role of the funders for the present study and, if applicable, for the original study on which the present article is based	14

*Give information separately for exposed and unexposed groups.

Note: An Explanation and Elaboration article discusses each checklist item and gives methodological background and published examples of transparent reporting. The STROBE checklist is best used in conjunction with this article (freely available on the Web sites of PLoS Medicine at http://www.plosmedicine.org/, Annals of Internal Medicine at http://www.annals.org/, and Epidemiology at http://www.epidem.com/). Information on the STROBE Initiative is available at http://www.strobe-statement.org.

For peer review only

BMJ Open

Clinical features and natural history of the first 2,073 suspected COVID-19 cases in the Corona São Caetano primary care programme: a prospective cohort study

Journal:	*BMJ Open*
Manuscript ID	bmjopen-2020-042745.R2
Article Type:	Original research
Date Submitted by the Author:	02-Dec-2020
Complete List of Authors:	Leal, Fabio ; Universidade Municipal de São Caetano do Sul; Instituto Nacional de Cancer<br>Mendes-Correa, Maria; Universidade de São Paulo, Instituto de Medicina Tropical (LIM-52, LIM-46, LIM-49) and Departamento de Moléstias Infecciosas e Parasitarias<br>Buss, Lewis; Universidade de São Paulo, Instituto de Medicina Tropical (LIM-52, LIM-46, LIM-49) and Departamento de Moléstias Infecciosas e Parasitarias<br>Costa, Silvia; Universidade de São Paulo, Instituto de Medicina Tropical (LIM-52, LIM-46, LIM-49) and Departamento de Moléstias Infecciosas e Parasitarias<br>Bizario, Joao; Universidade Municipal de São Caetano do Sul<br>de Souza, Sonia ; Universidade Municipal de São Caetano do Sul<br>Thomaz, Osorio; Instituto de Pesquisas Tecnológicas<br>Tozetto-Mendoza, Tania; Universidade de São Paulo, Instituto de Medicina Tropical (LIM-52, LIM-46, LIM-49) and Departamento de Moléstias Infecciosas e Parasitarias<br>Villas-Boas, Lucy; Universidade de São Paulo, Instituto de Medicina Tropical (LIM-52, LIM-46, LIM-49) and Departamento de Moléstias Infecciosas e Parasitarias<br>Oliveira, Lea; Universidade de São Paulo, Instituto de Medicina Tropical (LIM-52, LIM-46, LIM-49) and Departamento de Moléstias Infecciosas e Parasitarias<br>Grespan, Regina; Universidade Municipal de São Caetano do Sul<br>Capuani, Ligia; Modular Research System<br>Buccheri, Renata; Universidade de São Paulo, Instituto de Medicina Tropical (LIM-52, LIM-46, LIM-49) and Departamento de Moléstias Infecciosas e Parasitarias<br>Domingues, Helves; Modular Research System<br>Alexander, Neal; London School of Hygiene & Tropical Medicine<br>Mayaud, Philippe; London School of Hygiene & Tropical Medicine<br>Sabino, Ester; Universidade de São Paulo, Instituto de Medicina Tropical (LIM-52, LIM-46, LIM-49) and Departamento de Moléstias Infecciosas e Parasitarias
**Primary Subject Heading**:	Infectious diseases
Secondary Subject Heading:	Epidemiology, Public health, Global health

Keywords:	PRIMARY CARE, Epidemiology < TROPICAL MEDICINE, INFECTIOUS DISEASES, PUBLIC HEALTH

SCHOLARONE™
Manuscripts

I, the Submitting Author has the right to grant and does grant on behalf of all authors of the Work (as defined in the below author licence), an exclusive licence and/or a non-exclusive licence for contributions from authors who are: i) UK Crown employees; ii) where BMJ has agreed a CC-BY licence shall apply, and/or iii) in accordance with the terms applicable for US Federal Government officers or employees acting as part of their official duties; on a worldwide, perpetual, irrevocable, royalty-free basis to BMJ Publishing Group Ltd ("BMJ") its licensees and where the relevant Journal is co-owned by BMJ to the co-owners of the Journal, to publish the Work in this journal and any other BMJ products and to exploit all rights, as set out in our licence.

The Submitting Author accepts and understands that any supply made under these terms is made by BMJ to the Submitting Author unless you are acting as an employee on behalf of your employer or a postgraduate student of an affiliated institution which is paying any applicable article publishing charge ("APC") for Open Access articles. Where the Submitting Author wishes to make the Work available on an Open Access basis (and intends to pay the relevant APC), the terms of reuse of such Open Access shall be governed by a Creative Commons licence – details of these licences and which Creative Commons licence will apply to this Work are set out in our licence referred to above.

Other than as permitted in any relevant BMJ Author's Self Archiving Policies, I confirm this Work has not been accepted for publication elsewhere, is not being considered for publication elsewhere and does not duplicate material already published. I confirm all authors consent to publication of this Work and authorise the granting of this licence.

For peer review only

Clinical features and natural history of the first 2,073 suspected COVID-19 cases in the Corona São Caetano primary care programme: a prospective cohort study

Fabio E Leal[1,2]* (PhD), Maria C Mendes-Correa[3]* (PhD), Lewis F Buss[3]*# (MBBS), Silvia F Costa[3] (PhD), Joao CS Bizario[1] (PhD), Sonia RP de Souza[1] (PhD), Osorio Thomaz[4] (PhD), Tania R Tozetto-Mendoza[1] (PhD), Lucy S Villas-Boas[3], Lea CO Silva[3] (PhD), Regina MZ Grespan (MD)[1], Ligia Capuani[5] (PhD), Renata Buccheri[3] (MD), Helves Domingues (PhD)[5], Neal DE Alexander[6] (PhD), Philippe Mayaud[6] (MD), Ester C Sabino[3] (PhD)

* These authors contributed equally

Affiliations

1. Universidade Municipal de São Caetano do Sul (USCS), Rua Santo Antonio, São Caetano do Sul, Brazil
2. National Cancer Institute (INCA), Praça Cruz Vermelha, Rio de Janeiro, Brazil
3. Instituto de Medicina Tropical (LIM-52, LIM-46, LIM-49) and Departamento de Moléstias Infecciosas e Parasitarias da Faculdade de Medicina da Universidade de São Paulo Avenida Dr. Enéas Carvalho de Aguiar, São Paulo, Brazil
4. Instituto de Pesquisas Tecnológicas do Estado de São Paulo - IPT
5. Modular Research System, Avenida Paulista, São Paulo, Brazil
6. London School of Hygiene and Tropical Medicine (LSHTM), Keppel Street, London, United Kingdom

Corresponding author:

Dr Lewis F Buss

Instituto de Medicina Tropical da Faculdade de Medicina da Universidade de São Paulo

lewisbuss@usp.br

KEY WORDS: SARS-CoV-2, COVID-19, pandemic, community, primary care, Brazil

For peer review only

ABSTRACT

Background: Despite most cases not requiring hospital care, there are limited community-based clinical data on COVID-19.

Methods: The Corona São Caetano program is a primary care initiative providing care to all residents with COVID-19 in São Caetano do Sul, Brazil. It was designed to capture standardised clinical data on community COVID-19 cases. After triage of potentially severe cases, consecutive patients presenting to a multimedia screening platform between 13th April and 13th May 2020 were tested at home with SARS-CoV-2 reverse transcriptase (RT) PCR; positive patients were followed up for 14 days with phone calls every 2 days. RT-PCR-negative patients were offered additional SARS-CoV-2 serology testing to establish their infection status. We describe the clinical, virologic and natural history features of this prospective population-based cohort.

Findings: Of 2,073 suspected COVID-19 cases, 1,583 (76.4%) were tested by RT-PCR, of whom 444 (28.0%, 95%CI: 25.9-30.3) were positive; 604/1,136 (53%) RT-PCR-negative patients underwent serology, of whom 52 (8.6%) tested SARS-CoV-2 seropositive. The most common symptoms of confirmed COVID-19 were cough, fatigue, myalgia and headache; whereas self-reported fever (OR 3.0, 95%CI: 2.4-3.9), anosmia (OR 3.3, 95%CI: 2.6-4.4), and ageusia (2.9, 95%CI: 2.3-3.8) were most strongly associated with a positive COVID-19 diagnosis by RT-PCR or serology. RT-PCR cycle thresholds were lower in men, older patients, those with fever and arthralgia, and closer to symptom onset. The rates of hospitalization and death among 444 RT-PCR-positive cases were 6.7% and 0.7%, respectively, with older age and obesity more frequent in the hospitalized group.

Conclusion: COVID-19 presents in a similar way to other mild community-acquired respiratory diseases, but the presence of fever, anosmia, and ageusia can assist the specific diagnosis. Most patients recovered without requiring hospitalization with a low fatality rate compared to other hospital-based studies.

For peer review only - http://bmjopen.bmj.com/site/about/guidelines.xhtml

Strengths and limitations of this study

1. The clinical features of COVID-19 have mostly been described in hospital-based studies which are biased towards severe disease
2. We report a prospective cohort of suspected and confirmed COVID-19 cases from a primary care initiative in the Brazilian municipality of São Caetano do Sul
3. By systematically testing consecutive suspected community cases with molecular and serological tests we were able to address the diagnostic value of clinical features of mild-moderate COVID-19 in primary care
4. Prospective follow-up of confirmed cases and linkage with hospital datasets allowed us to describe the natural history of a primary care COVID-19 population
5. A limitation of the work was that not all PCR-negative participants underwent serology testing due to loss to follow-up

For peer review only

INTRODUCTION

A comprehensive public health response is vital but difficult to achieve during an epidemic. The COVID-19 pandemic, caused by the novel severe acute respiratory syndrome coronavirus 2 (SARS-CoV-2), started in China in late 2019.[1] According to the World Health Organization (WHO)[2,3] and others[4,5], the ideal early response should have been multipronged, with identification, isolation, treatment and contact tracing of symptomatic cases, relying on a strong testing programme. Primary health care is well placed to implement such a response, by identifying cases early and managing them in a way that minimizes overcrowding of emergency rooms and intensive care units.[6,7] Real-time data analysis coming from these primary care response systems can inform policy decisions.

Primary health care (PHC) in Brazil is provided by the publicly funded Unified Health System (SUS – Portuguese acronym) within the family health strategy (*Estratégia Saúde da Família*). Provision of care is centred around a healthcare unit with a multi-professional team that is responsible for all residents in the immediate catchment area[8]. Nearly two-thirds of the Brazilian population is covered by the family health strategy[8].

In Brazil, the first case of COVID-19 was identified in the city of São Paulo on 26th February 2020.[9] As of 1st Dec 2020 there were over 6 million confirmed cases nationally, with São Paulo contributing a fifth of these.[10] The reasons for the exceptionally large epidemic in Brazil have been discussed elsewhere[11–13]. In March 2020, the Municipal Health Department of the municipality of São Caetano do Sul – part of the Greater Metropolitan Region of São Paulo – began to develop a clinical and testing platform to organize its COVID-19 response. The aim was to provide universal detection and management of symptomatic cases and their contacts. The platform was developed in partnership with two local universities – the Municipal University of São Caetano do Sul (USCS) and the University of Sao Paulo (USP) – and called "Corona São Caetano".

Large scale community-based observational cohorts are difficult to establish under epidemic circumstances, particularly if the risk of exposure for research personnel is high. Hence, most COVID-19 epidemiological and clinical studies have been hospital-based,[14–16] and therefore tend to include more severe cases whose findings may not be generalizable to the general

For peer review only - http://bmjopen.bmj.com/site/about/guidelines.xhtml

population[17], although some limited descriptions from ambulatory settings are available[18–20].

The objectives of this study were to describe the epidemiological indicators of the early phase of the programme rollout; and to describe the clinical, virological and natural history features (including hospitalization and deaths) of SARS-CoV-2 infection among patients identified in primary care.

METHODS

Setting

The municipality of São Caetano do Sul has a population of 161,000 inhabitants.[21] The city's population is older than the Brazilian population[21] and its Human Development Index is one of the highest in the country. Nearly all (97.4%) children aged 6-14 are in education and 31% of the population have completed higher education[22] (Brazilian national average is 11%).

Corona São Caetano platform

The objective of the platform was to offer clinical care for patients with flu syndrome and suspected COVID-19. Through the multimedia platform (website of phone call), patients could be triaged and guided in relation to their clinical needs and tested, without having to leave their homes or go to health facilities, unless seriously ill. This strategy aimed at reducing the workload in health units and the risk of SARS-CoV-2 transmission in the population served by these health units. Patients' GPs were informed of lab results and had access to clinical data stored in the platform. GPs were expected to call patients being assisted by the platform and provide medical assistance through home visits or at the primary care clinic if needed. In general, the drugs prescribed through the platform were restricted to analgesics and antipyretics. The platform was designed so that clinical information was collected in a standardized way for research purposes.

Residents of the municipality aged 12 years and older with suspected COVID-19 symptoms were encouraged, through local media reports, to contact the dedicated Corona São Caetano platform via the website or by phone. They were invited to complete an initial screening

For peer review only

questionnaire that included socio-demographic data; information on symptoms type, onset and duration; and recent contacts.

Patients meeting the suspected COVID-19 case definition (i.e., having at least two of the following symptoms: fever, cough, sore throat, coryza, or change in/loss of smell (anosmia); or one of these symptoms plus at least two other symptoms consistent with COVID-19) were further evaluated, whilst people not meeting these criteria were reassured, advised to stay at home and contact the service again if they were to develop new symptoms or worsening of current ones. The case definition was developed in consultation with infectious disease and primary care specialists to encompass the known symptoms of COVID-19 and is similar to the Brazilian national case definition[23]. Patients were then called by a medical student to complete a risk assessment. All pregnant women, and patients meeting pre-defined triage criteria for severe disease (see Supplemental Material), were advised to attend a hospital service - either an emergency department or outpatient service, depending on availability. All other patients were offered a home visit for self-collection of a nasopharyngeal swab.

Sample collection

Patients self-collected nasopharyngeal swabs (NPS – both nostrils and throat) at their own homes under the supervision of trained healthcare personnel. We sent a link to an instructional video (https://youtu.be/rWZzV2ZP7KY) before the home visit to provide guidance on self-collection procedures. Nasopharyngeal swabs for the molecular detection of SARS-CoV-2 has been recommended as an alternative method of collection for samples from patients with suspected COVID-19[24], as well as other respiratory diseases, and has the advantage of reducing the chance of aerosol transmission to healthcare professionals. Healthcare personnel were instructed to maintain a distance of six feet from the patient and to wear personal protective equipment at all times. Samples were immediately put on a cool box between 2-8°C and stored at 4°C in a fridge until shipment to the lab within 24 hours.

Follow-up procedures

Patients testing SARS-CoV-2 RT-PCR positive were followed up to 14 days[25] (a maximum of 7 phone calls) from completion of their initial questionnaire. They were contacted every 48 hours by a medical student who completed another risk assessment and recorded any ongoing

For peer review only - http://bmjopen.bmj.com/site/about/guidelines.xhtml

or new symptoms. The purpose of the follow-up was to assess clinical evolution. Where patients were judged to be deteriorating or developing severe disease they were signposted to secondary care services. Patients testing RT-PCR negative were followed up by the primary health care program for their residential area. They were advised to contact the platform for a new consultation if they developed new symptoms. Starting on May 19th, when serological testing became available, RT-PCR-negative patients were re-contacted to offer antibody (IgG/IgM combined) testing 14 days after their initial registration as long as they had become asymptomatic.

Study dates

The Corona São Caetano programme was launched on 6th April 2020, with a one week pilot phase designed to test instruments before roll-out. For this analysis, we included all patients making their first contact with the programme in its first month, ie between 13th April and 13th May 2020. The period of follow-up (last date of data extraction) was 4th June 2020, to account for the accrual period (three weeks) of possible hospitalizations in the last included patients.

Laboratory methods

Due to shortages of some reagents, we used two RT-PCR platforms at different times during the study: ALTONA RealStar® SARS-CoV-2 RT-PCR Kit 1.0 (Hamburg, Germany) and the Mico BioMed RT-qPCR kit (Seongnam, South Korea). For serology we tested 10µL of serum or plasma (equivalent in performance) using a qualitative rapid chromatographic immunoassay (Wondfo Biotech Co., Guangzhou, China), that jointly detects anti-SARS-CoV-2 IgG/IgM. The assay has been found to have a sensitivity of 81.5% and specificity of 99.1% in a US study[26]. In our local validation, after two weeks of symptoms, the sensitivity in 59 RT-PCR confirmed cases was 94.9%, and specificity in 106 biobank samples from 2019 was 100%.

Statistical methods

We estimated the contribution of our platform to total number of COVID-19 cases diagnosed in São Caetano do Sul. To do this, we compared the number of cases diagnosed in our

For peer review only

programme with official data released by the Municipal Department of Health in its daily bulletins (accessed here https://coronavirus.saocaetanodosul.sp.gov.br).

Clinical and demographic data were extracted directly from the Corona São Caetano information system. To analyse clinical presentation, we first calculated the proportion and exact binomial 95% confidence intervals (CI) of cases reporting each symptom in the three testing groups: SARS-CoV-2 RT-PCR positive; RT-PCR negative / seropositive; and RT-PCR negative / seronegative. We next combined RT-PCR and serology positive cases to make a confirmed COVID-19 group, and those negative on both tests to make a SARS-CoV-2 negative control group. We express the association between each symptom and a positive COVID-19 diagnosis as odds ratios (OR) and 95% CIs.

Next, we assessed associations between RT-PCR cycle thresholds (Cts) and other clinical features. ALTONA and MiCo BioMed RT-PCR kits each separately amplify two different SARS-CoV-2 viral genes, as such each patient had two Ct values. There was a high concordance between Cts for the two genes within each kit (Figure S1), and we opted therefore to use the mean of the two Ct values for each patient in all analyses. We calculated univariable associations between Cts and age, sex, delay from symptom onset to NPS collection, and presenting symptoms using simple linear regression. We then built a multivariable linear regression model to assess independent associations between presenting symptoms and RT-PCR Cts. As age, sex, and time of swab collection may confound this relationship we included these variables, as well as the RT-PCR platform (ALTONA vs MiCo BioMed), as covariates in the model.

For RT-PCR positive patients, hospitalizations and deaths were extracted from the study platform. To extend the follow-up period and to capture RT-PCR negative patients and those initially triaged to hospital (no study follow-up), hospitalization and vital status was confirmed by linkage with two administrative databases: the municipal epidemiological surveillance dataset, as well as the state-wide influenza-like illness notification system (SIVEP-Gripe). Linkage was last performed on 5th June 2020, 23 days after the last patient was enrolled, by the author SRPS who did not have access to the full analytic dataset. This author searched the SIVEP-Gripe system and the municipal epidemiological surveillance dataset using full name and date of birth. Categorical patient characteristics were compared between patients requiring and those not requiring hospitalization using a Chi-squared or

For peer review only - http://bmjopen.bmj.com/site/about/guidelines.xhtml

Fisher exact test. Continuous variables were compared using the Wilcoxon rank sum test. A multivariate analysis was not conducted due to the small number of individuals experiencing this outcome.

The cohort sample included consecutive cases presenting to the Corona São Caetano program and a formal sample size calculation was not performed. Missing data were excluded. All analyses were conducted in R Software for Statistical Computing, version 3.6.3.[27]

Ethics

The study was approved by the local ethics committee (Comissão de Ética para Análise de Projeto de Pesquisa - CAPPesq, protocol No. 13915, dated June 03, 2020). The committee waived the need for informed consent and allowed the development of an analytical dataset with no personal identification for the current analysis.

Patient and public involvement

Patients were not involved in the planning of this research.

RESULTS

Epidemiological and programmatic indicators

Over the study period, there were 2,073 presentations (49% phone call, 51% website), from 2,011 individual patients, that met the criteria for a suspected COVID-19 case (See Figure 1 for study flow). At initial phone interview, 132 (6%) potential cases were advised to go directly to a health service based on the triage questions, and 12 (0.6%) because of pregnancy. Only four (3%) of referred patients were admitted to hospital and none died.

In total 1,583 individual patients were tested with RT-PCR for SARS-CoV-2; 444 (28.0%, 95%CI: 25.9-30.3) were positive. The proportion of positive results was stable over the study (Figure S2). Among the RT-PCR negative group, 604 (53% of 1,136) underwent serology testing, of whom 52 (8.6%, 95%CI: 6.6-11.1) were seropositive. The median [IQR] time from symptom onset to serology collection was 31 [26–37] days. The age-sex structure of patients

For peer review only - http://bmjopen.bmj.com/site/about/guidelines.xhtml

being tested differed from the underlying population of São Caetano do Sul (Figure S3) with an overrepresentation of working-age adults and women. At the beginning of programme role out, 25% of notified COVID-19 cases in São Caetano do Sul were diagnosed in our programme. Over the study period, adherence to the programme increased, and by May 13th, 2020, this figure had risen to 78%.

Of 444 RT-PCR positive patients eligible for longitudinal follow-up, 326 (73%) had their final follow-up visit at least 14 days after their initial presentation. Of the seven possible follow-up questionnaires, 384 (86%) COVID-19 patients completed three or more, and 162 (36%) completed all seven.

Participant characteristics

Patient characteristics are shown in Table 1. Although women were overrepresented in the cohort, there were proportionally more males in the RT-PCR positive and seropositive groups compared to the seronegative group. Of note, 55% of RT-PCR negative/seronegative patients had completed higher education compared to 35% RT-PCR-positive patients ($p < 0.001$, Chi-squared test). The median number of days from symptom onset to swab collection was 5.0 (IQR, 4.0-7.0) among RT-PCR positive patients and 6.0 (IQR, 4.0-8.3) among RT-PCR negative/seropositive patients ($p = 0.06$, Wilcoxon rank sum) (Figure S4). Chronic respiratory disease was less frequent in RT-PCR positive than dual-negative patients.

Symptoms of COVID-19

The prevalence of individual symptoms at presentation is shown in Figure 2A stratified by final diagnostic category. The most frequent symptoms among RT-PCR and seropositive patients were headache (82% and 75%), myalgia (80% and 80%), cough (77% and 63%), and fatigue (77% and 79%). Anosmia was present in 56% and 63% of RT-PCR positive and seropositive patients, respectively, compared to 30% in those testing doubly negative. A similar pattern was observed for ageusia (53% and 53% versus 30%). Upper respiratory tract symptoms - including coryza, blocked nose, ageusia, and anosmia - were more frequent in younger people (Figure 2B). The evolution of symptoms over time among RT-PCR positive patients is shown in Figure S5.

For peer review only

The odds ratios for testing positive for SARS-CoV-2 (RT-PCR or serology) associated with each presenting symptom are shown in Figure 3. The symptoms with strongest associations were anosmia (OR 3.3, 95%CI: 2.6-4.4), fever (3.0, 95%CI: 2.4-3.9) and ageusia (2.9, 95%CI: 2.3-3.8). The presence of sore throat (0.53, 95%CI: 0.41-0.68) and diarrhoea (0.72, 95%CI: 0.55-0.96) were associated with a negative SARS-CoV-2 test.

Associations between SARS-CoV-2 RT-PCR Cycle threshold (Ct) values, and demographic and clinical features

Figure 4 shows the associations between mean RT-PCR cycle threshold and demographic features and symptoms at presentation (the median [IQR] time from presentation to swab was 1 [1-2] day). Older age was associated with lower cycle thresholds, with a change in mean Ct of -0.05 (95%CI -0.09 to -0.01) for each additional year of age. The mean difference in Ct value was -1.36 (95% CI -2.49 to -0.23) in men compared to women. For each doubling in the number of days from symptom onset to swab collection the mean Ct value increased by 3.28 (95%CI 2.33 to 4.03). Presenting symptoms of fever and arthralgia were associated with lower Cts, whereas anosmia, ageusia, vomiting, diarrhoea, and nausea were associated with higher Cts (Figure 4 and Table S1). After adjustment for age, sex, delay from symptom onset, and RT-PCR platform used, fever (-0.06, 95%CI: -2.11 to -0.001) and arthralgia (-1.24, 95%CI: -2.18 to -0.10) remained associated with lower Cts, and anosmia (2.21, 95%CI: 1.0-3.29), ageusia (1.96, 95%CI: 0.88-3.0), and diarrhoea (1.36, 95%CI: 0.12-2.61) with higher Cts (Table S1).

Hospitalizations and deaths

Of the 444 RT-PCR positive patients, 30 (6.8%) had been hospitalized by 5th June 2020, when the database linkage was last updated, and three (0.7%) had died; in-hospital mortality was therefore 10% (3/30). In 28 cases the date of admission was available. The median time from symptom onset to hospital admission was 7 (range 2 to 14) days. Among 1,136 RT-PCR-negative patients, six (0.5%) had been admitted to hospital. One (<0.01% of 1,136) of these six patients died. None of the 604 RT-PCR negative patients that underwent serology were admitted to hospital or died. Table 2 compares patient characteristics by hospitalization status. Notably, hospitalized patients were older, had more cardiovascular comorbidities and were more frequently obese.

For peer review only - http://bmjopen.bmj.com/site/about/guidelines.xhtml

DISCUSSION

We present a community-based cohort of suspected COVID-19 cases recruited through a primary care initiative in the Brazilian municipality of São Caetano do Sul. Offering RT-PCR testing to all patients presenting with symptoms compatible with COVID-19, the positivity rate was 28%, with 8.6% of those testing negative subsequently found to be seropositive - i.e. > 35% of the cohort had a diagnosis of COVID-19. Anosmia, ageusia, and self-reported fever provided the greatest diagnostic value in identifying COVID-19. The rate of hospitalization and deaths among RT-PCR positive patients was low, at 6.8% and 0.7%, respectively. Our results provide important information on the clinical presentation, diagnostic testing and natural history of COVID-19 identified in the community.

The profile of suspected cases that tested positive for COVID-19 differed in some important respects from those testing negative. The lower educational level among positive cases suggests that, in São Caetano do Sul, the risk of exposure to COVID-19 follows a socioeconomic gradient, consistent with other findings from Brazil[13,28]. Although more women presented to the platform, proportionally more men tested positive, consistent with data from São Paulo showing a higher seroprevalence in men than women[11], but also potentially reflecting different health seeking behaviours. Comorbidities were mostly similar, although chronic respiratory disease was less frequent in those testing RT-PCR positive. This may be due to a proportion of presentations in those with chronic respiratory disease being explained by exacerbations of their underlying pathology from aetiologies other than SARS-CoV-2, as well as higher anxiety about COVID-19 in those with pre-existing respiratory disease.

Extrapolating the seropositivity rate among RT-PCR negative patients to the 532 who were not tested with serology, we estimate that an additional 46 seropositive cases would have been identified. As such, 18% (98/542) of COVID-19 cases were missed by RT-PCR in the setting of symptomatic presentations to primary care. This is similar to a pooled analysis showing a false-negative rate for RT-PCR of 20% at three days post-symptom onset.[29] Viral load peaks around the time of symptom onset and remains high over the first symptomatic week (also see Figure 4A).[30,31] Consistent with this, we found a slightly longer delay to swab

For peer review only

collection (due to delay in presentation to the platform) in RT-PCR false-negative patients than RT-PCR positive patients (Figure S4).

COVID-19 presents in a similar way to other respiratory viral illnesses. Indeed, in our cohort the most common symptoms of COVID-19 - such as cough, fatigue, headache, etc. - were reported with a similar frequency among patients testing negative. It is therefore important to have identified anosmia, ageusia, self-reported fever, myalgia, and anorexia as the symptoms with greatest value in the differential diagnosis of COVID-19 in primary care. This is consistent with systematic review evidence highlighting anosmia and ageusia as key diagnostic features of COVID19[32]. It is of note that 30% of jointly RT-PCR and serology negative patients reported these symptoms, indicating that although indicative of COVID-19, the specificity of these symptoms is not high enough to rule in the diagnosis alone. Sore throat and diarrhoea - both considered symptoms of COVID-19 in other settings –[33] were more frequently due to other possible aetiologies in this primary care context.

These results are robust for a number of reasons. Firstly, our sample is representative of the population of interest - i.e. consecutive patients with suspected COVID-19 in the community - instead of extrapolating from hospital cases. Symptom data were collected prospectively, eliminating recall or interviewer bias. Finally, we have a control group of patients who were negative for both RT-PCR and serology, minimizing misclassification due to false negative RT-PCR.

In our study, the proportion of patients with a positive SARS-CoV-2 RT-PCR requiring hospitalization was low (7%). Early reports from China were of 13.8% of cases being severe[34], but this value was lower when under ascertainment of cases was accounted for.[35,36] This is because our cohort reflects mild to moderate cases, as severely ill patients are likely to have attended hospital directly. As such, only 3% of patients we triaged to attend health services were ultimately hospitalized, possibly due to self-selection of patients presenting to our service. Supporting this, our overall case fatality ratio among RT-PCR positive patients was 0.7%. The rate of hospitalization was lower (0.5%) in those testing PCR-negative. These patients were admitted with a severe acute respiratory syndrome of an aetiology other than SARS-CoV-2. The 14-fold higher admission rate among PCR-positive cases highlights the importance of molecular testing for SARS-CoV-2 in patients presenting with features of respiratory viral illness to primary care.

For peer review only - http://bmjopen.bmj.com/site/about/guidelines.xhtml

As expected, the main determinant of Ct was the delay between symptom onset and swab collection, mostly due to the delay in reporting to the platform. After adjusting for this, as well as age and sex, we found that a self-reported fever and arthralgia were associated with lower Cts. The presence of these symptoms may identify patients with a higher viral load in the community. However, these results should be seen as purely exploratory, and the wide spread of Ct values around the regression line precludes a direct clinical application at present.

Our study has some limitations. Firstly, serology was not performed on all RT-PCR negative patients due to on-going symptoms, loss to follow-up, or patient refusal. Of note, none of the RT-PCR-negative patients that were admitted to hospital underwent serology testing. This suggests that patients who were not tested with serology may have had a higher prevalence of COVID-19 than those that were tested. In addition, imperfect serology test performance (81% sensitivity)[26] will introduced false-negative results. Taken together, these biases may have underestimated the true seroprevalence among RT-PCR-negative cases, as well as the false-negative rate of RT-PCR. The latter calculation may also have been influenced by the inclusion of RT-PCR positive patients in the denominator, introducing an incorporation bias.[37] Furthermore, the association between symptoms and COVID-19 diagnosis was based on the comparison with doubly PCR and serology negative individuals. It is not clear how the exclusion of individuals that did not undergo serology testing would have influenced these associations. Finally, patients were not involved in the planning of the Corona platform or the research proposal.

A key strength to our study relates to the provision of primary healthcare in Brazil and its symbiosis with medical training nationwide. Primary health care - within the family health strategy (*Estratégia Saúde da Família*) - is centered around a healthcare unit with a multi-professional team that is responsible for all residents in the immediate catchment area [8]. São Caetano do Sul has enough GP units within the family health strategy that all residents have access to primary care. Medical students from the municipal university (USCS) are integrated into the primary healthcare teams and progressively trained from the first year of medical school. Our initiative took advantage of this existing system, with the addition of an online platform allowing remote clinical assessment and follow-up. The suspension of normal clinical training at the medical school provided the workforce. The partnership with the

For peer review only - http://bmjopen.bmj.com/site/about/guidelines.xhtml

University of São Paulo, which provided the laboratory diagnostics, created the unique opportunity to establish our prospective community cohort of suspected and confirmed COVID-19 cases. But we believe that this infrastructure could be implemented in other regions with less resources. Other respiratory disease such as influenza, measles, or tuberculosis may benefit from similar approach. However, further evaluation of the impact of the Corona Platform are required.

CONCLUSION

Systematic testing of all suspected COVID-19 cases was feasible within primary care services in a Brazilian municipality. Anosmia, agueusia, and fever provide the greatest diagnostic discrimination from other similar primary care presentations. Home-care is a valid approach for most of these patients with a low rate of hospitalization and death.
Our programme model – integrating multimedia technology, telehealth with universal access to primary care – may be successful in other contexts.

CONTRIBUTION STATEMENT

FEL, MCMC, SFC, MC, RB, and ECS conceived and designed the study. FEL, RMZG, and JCSB provided clinical oversight and supervision of medical students. FEL, MCMC, LFB, HD, OT, LC, and SRPS collected and curated the data. MCMC, TRTM, LSVB, and LCOS performed the laboratory analysis. LFB performed the formal statistical analysis with assistance from FEL, SRPS, NDEA, PM, ECS and OT. LFB, FEL, PM and ECS wrote the first draft, and all authors reviewed, contributed to and approved the final version.

CONFLICTS OF INTEREST STATEMENT

The authors have no conflicts of interests. FL, RG, and JB were involved in providing clinical care within the Corona São Caetano Platform.

FUNDING STATEMENT

The municipal health department of São Caetano do Sul (Secretaria Municipal de Saúde da Prefeitura de São Caetano do Sul) funded the establishment and implementation of the

For peer review only

platform. We also acknowledge an award from FAPESP (2018/14389-0) and the UK Medical Research Council (MR/S0195/1) to the Brazil-UK Centre for Arbovirus Discovery, Diagnosis, Genomics and Epidemiology (CADDE).

DATA SHARING STATEMENT

Anonymized data are available at https://figshare.com/articles/dataset/Clinical_features_and_natural_history_of_the_first_2_073_suspected_COVID-19_cases_in_the_Corona_S_o_Caetano_primary_care_programme_a_prospective_cohort_study/13322474

REFERENCES

1. World Health Organization. (2020). Pneumonia of unknown cause – China. (https://www.who.int/csr/don/05-january-2020-pneumonia-of-unkown-cause-china/en/, accessed 2020-06-18).
2. World Health Organization. Critical preparedness, readiness and response actions for COVID-19 (Interim Guidance) (https://www.who.int/publications-detail/critical-preparedness-readiness-and-response-actions-for-covid-19, accessed 15 June 2020).
3. Bedford, J. et al. COVID-19: towards controlling of a pandemic. The Lancet **395**, 1015–1018 (2020).
4. Hellewell, J. et al. Feasibility of controlling COVID-19 outbreaks by isolation of cases and contacts. Lancet Glob Health **8**, e488–e496 (2020).
5. Dunlop, C., Howe, A., Li, D. & Allen, L. N. The coronavirus outbreak: the central role of primary care in emergency preparedness and response. BJGP Open **4**, bjgpopen20X101041 (2020).

For peer review only - http://bmjopen.bmj.com/site/about/guidelines.xhtml

For peer review only

6. World Health Organization. Regional Office for the Western Pacific. (2020). Role of primary care in the COVID-19 response. (https://apps.who.int/iris/handle/10665/331921, accessed 15 June, 2020).
7. Redwood-Campbell, L. & Abrahams, J. Primary Health care and Disasters—The Current State of the Literature: What We Know, Gaps and Next Steps. Prehosp. Disaster med. **26**, 184–191 (2011).
8. Macinko, J. & Harris, M. J. Brazil's Family Health Strategy — Delivering Community-Based Primary Care in a Universal Health System. NEJM **372**, 2177–2181 (2015).
9. World Health Organization. Situation report 27 (2020). (https://www.who.int/emergencies/diseases/novel-coronavirus-2019/situation-reports, accessed 15 June 2020).
10. Brazilian Ministry of Health. (https://covid.saude.gov.br/).
11. Buss, L. F. et al. COVID-19 herd immunity in the Brazilian Amazon. http://medrxiv.org/lookup/doi/10.1101/2020.09.16.20194787 (2020) doi:10.1101/2020.09.16.20194787.
12. de Souza, W. M. et al. Epidemiological and clinical characteristics of the COVID-19 epidemic in Brazil. Nat Hum Behav **4**, 856–865 (2020).
13. Hallal, P. C. et al. SARS-CoV-2 antibody prevalence in Brazil: results from two successive nationwide serological household surveys. The Lancet Global Health S2214109X20303879 (2020) doi:10.1016/S2214-109X(20)30387-9.

For peer review only

14. Docherty, A. B. et al. Features of 20 133 UK patients in hospital with covid-19 using the ISARIC WHO Clinical Characterisation Protocol: prospective observational cohort study. BMJ m1985 (2020) doi:10.1136/bmj.m1985.
15. Richardson, S. et al. Presenting Characteristics, Comorbidities, and Outcomes Among 5700 Patients Hospitalized With COVID-19 in the New York City Area. JAMA **323**, 2052 (2020).
16. Zhou, F. et al. Clinical course and risk factors for mortality of adult inpatients with COVID-19 in Wuhan, China: a retrospective cohort study. The Lancet **395**, 1054–1062 (2020).
17. Rauh, A. L. & Linder, J. A. Covid-19 care before, during, and beyond the hospital. BMJ m2035 (2020) doi:10.1136/bmj.m2035.
18. Lapostolle, F. et al. Clinical features of 1487 COVID-19 patients with outpatient management in the Greater Paris: the COVID-call study. Intern Emerg Med **15**, 813–817 (2020).
19. Mizrahi, B. et al. Longitudinal symptom dynamics of COVID-19 infection in primary care. http://medrxiv.org/lookup/doi/10.1101/2020.07.13.20151795 (2020) doi:10.1101/2020.07.13.20151795.
20. Williamson, E. J. et al. Factors associated with COVID-19-related death using OpenSAFELY. Nature **584**, 430–436 (2020).
21. Instituto Brasileiro de Geografia e Estatística (IBGE). (2020). São Caetano do Sul / Panorama. (https://cidades.ibge.gov.br/brasil/sp/sao-caetano-do-sul/panorama).

For peer review only

22. Human Development Atlas Brazil. (2020). São Caetano do Sul (http://atlasbrasil.org.br/2013/pt/perfil_m/sao-caetano-do-sul_sp).

23. Brazilian COVID-19 case definition and notification criteria. Brazilian Ministry of Health (https://coronavirus.saude.gov.br/definicao-de-caso-e-notificacao).

24. Akmatov, M. K., Gatzemeier, A., Schughart, K. & Pessler, F. Equivalence of Self- and Staff-Collected Nasal Swabs for the Detection of Viral Respiratory Pathogens. PLoS ONE **7**, e48508 (2012).

25. Walsh, K. A. et al. SARS-CoV-2 detection, viral load and infectivity over the course of an infection. Journal of Infection **81**, 357–371 (2020).

26. Whitman, J. D. et al. Test performance evaluation of SARS-CoV-2 serological assays. medRxiv 2020.04.25.20074856 (2020) doi:10.1101/2020.04.25.20074856.

27. R Core Team. R: A language and environment for statistical computing. R Foundation for Statistical Computing, Vienna, Austria. URL https://www.R-project.org/. (2019).

28. Baqui, P., Bica, I., Marra, V., Ercole, A. & van der Schaar, M. Ethnic and regional variations in hospital mortality from COVID-19 in Brazil: a cross-sectional observational study. The Lancet Global Health **8**, e1018–e1026 (2020).

29. Kucirka, L. M., Lauer, S. A., Laeyendecker, O., Boon, D. & Lessler, J. Variation in False-Negative Rate of Reverse Transcriptase Polymerase Chain Reaction–Based SARS-CoV-2 Tests by Time Since Exposure. Ann Int Med M20-1495 (2020) doi:10.7326/M20-1495.

For peer review only - http://bmjopen.bmj.com/site/about/guidelines.xhtml

30. To, K. K.-W. et al. Temporal profiles of viral load in posterior oropharyngeal saliva samples and serum antibody responses during infection by SARS-CoV-2: an observational cohort study. Lancet Inf Dis **20**, 565–574 (2020).

31. He, X. et al. Temporal dynamics in viral shedding and transmissibility of COVID-19. Nat Med **26**, 672–675 (2020).

32. Carpenter, C. R., Mudd, P. A., West, C. P., Wilber, E. & Wilber, S. T. Diagnosing COVID-19 in the Emergency Department: A Scoping Review of Clinical Examinations, Laboratory Tests, Imaging Accuracy, and Biases. Acad Emerg Med **27**, 653–670 (2020).

33. Vetter, P. et al. Clinical features of covid-19. BMJ m1470 (2020) doi:10.1136/bmj.m1470.

34. T. Team, Vital Surveillances: The Epidemiological Characteristics of an Outbreak of 2019 Novel Coronavirus Diseases (COVID-19)-China. China CDC Weekly **2**, 113–122 (2020).

35. Salje, H. et al. Estimating the burden of SARS-CoV-2 in France. Science eabc3517 (2020) doi:10.1126/science.abc3517.

36. Verity, R. et al. Estimates of the severity of coronavirus disease 2019: a model-based analysis. Lancet Infect Dis **20**, 669–677 (2020).

37. Worster, A. & Carpenter, C. Incorporation bias in studies of diagnostic tests: how to avoid being biased about bias. CJEM **10**, 174–175 (2008).

For peer review only

For peer review only

For peer review only - http://bmjopen.bmj.com/site/about/guidelines.xhtml

TABLE LEGENDS

Table 1 Demographic and clinical characteristics of 1,048 suspected COVID-19 cases undergoing diagnostic testing in the Corona São Caetano program. * Security, emergency services, supermarket, public transport, and pharmacy workers. IQR: interquartile range; HCW: health care workers, COPD: chronic obstructive pulmonary disease. Missing data – educational level 2; essential occupation 2; body mass index 4; cardiovascular disease 28; diabetes 31 mellitus; chronic resp. disease 65; chronic kidney disease 27; COPD 28. P-values calculated by Chi-squared, Fisher exact, or Wilcoxon rank sum.

Table 2 Characteristics of RT-PCR positive patients stratified by hospitalization status. Missing data – body mass index 2; cardiovascular disease 12; diabetes mellitus 12; chronic respiratory disease 29; COPD 11; chronic kidney disease 12; COPD - chronic obstructive pulmonary disease; IQR - interquartile range.

FIGURE LEGENDS

Figure 1 Patient flowchart for the Corona São Caetano platform between 13th April and 13th May 2020. In the upper section (white background) the numbers correspond to individual presentations to the system; among suspected cases 2,073 suspected cases, 60 had two presentations and one had three. In the lower section (grey background) numbers correspond to individual patients making up the final analytic groups.

Figure 2 Panel A presents prevalence (point) and exact binomial 95% confidence intervals (vertical lines) of symptoms at presentation among patients with suspected COVID-19 according to RT-PCR result and serostatus (A). Panels B and C present the prevalence of presenting symptoms among patients with COVID-19 (RT-PCR and serology positive) stratified by age (B) and sex (C).

Figure 3 Odds ratios (black dot) and 95% confidence intervals (lines) for testing positive for COVID-19 (RT-PCR positive or serology positive) associated with the presence of each presenting symptom. Horizontal axis is on log scale. Point estimates of odds ratios are shown inline with their corresponding symptom.

For peer review only

Figure 4 Relationship between mean RT-PCR cycle threshold (Ct) and day of illness course when the nasopharyngeal swab was collected (A), patient age (B), patient sex (C), and different symptoms at presentation. Panels A and B show the best fit linear regression lines, panels C and D are violin plots (rotated kernel density plots showing the full distribution of data) of the Ct values with median (black dot) and interquartile range (black line).

For peer review only

Table 1

	**RT-PCR +ve (G1)** N = 444 n (%) or median (IQR)	**RT-PCR -ve Sero +ve (G2)** N=52 n (%) or median (IQR)	**RT-PCR -ve Sero -ve (G3)** N = 552 n (%) or median (IQR)	**p-value** G1 versus G2	**p-value** G1 versus G3
**Sex**					
Male	200 (45·0)	23 (44·2)	185 (33·5)		
Female	244 (55·0)	29 (55·8)	367 (66·5)	1·0	<0·001
**Age groups (years)**					
10 to 19	29 (6·5)	1 (1·9)	25 (4·5)		
20 to 39	197 (44·4)	17 (32·7)	236 (42·8)		
40 to 59	158 (35·6)	28 (53·8)	218 (39·5)		
60+	60 (13·5)	6 (11·5)	73 (13·2)	0·07	0·40
**Educational level**					
Up to primary education	75 (16·9)	7 (13·5)	56 (10·2)		
High school	214 (48·3)	19 (36·5)	194 (35·2)		
University	154 (34·8)	26 (50·0)	301 (54·6)	0·10	<0·001
**Essential Occupation**					
Non-HCW essential job *	137 (30·9)	12 (23·1)	148 (26·9)		
Carers	10 (2·3)	0 (0·0)	8 (1·5)		
HCW	32 (7·2)	5 (9·6)	73 (13·2)		
No	264 (59·6)	35 (67·3)	322 (58·4)	0·45	0·01
**Body mass index (kg/m^2)**					
<25	151 (34·2)	22 (42·3)	211 (38·4)		
25-29	182 (41·2)	17 (32·7)	187 (34·0)		
30-35	79 (17·9)	9 (17·3)	112 (20·4)		
35+	30 (6·8)	4 (7·7)	40 (7·3)	0·62	0·14
**Comorbidities**					
Cardiovascular disease	88 (20·4)	9 (17·6)	129 (24·0)	0·89	0·40
Diabetes mellitus	48 (11·1)	4 (7·8)	39 (7·3)	0·86	0·12
Any chronic resp. disease	37 (8·9)	9 (18·0)	79 (15·3)	0·13	0·01
COPD	24 (5·5)	5 (9·8)	54 (10·1)	0·47	0·03
Chronic kidney disease	1 (<1)	0 (0·0)	3 (1·0)	1·0	0·83
**Time from symptom onset to swab collection (days)**, median (IQR)	5·0 (4·0-7·0)	6·0 (4·0-8·3)	6·0 (4·0-9·0)	0·06	<0·001

For peer review only

For peer review only - http://bmjopen.bmj.com/site/about/guidelines.xhtml

Table 2

	Hospitalized<br>n=30<br>n (%) or median (IQR)	Not hospitalized<br>n=414<br>n (%) or median (IQR)	p-value
**Age (years)**			
10 to 19	1 (3)	28 (97)	
20 to 39	6 (3)	191 (97)	
40 to 59	14 (9)	144 (91)	
60+	9 (15)	51 (85)	0·006
**Sex**			
Female	16 (7)	228 (93)	
Male	14 (7)	186 (93)	0·852
**Comorbidities**			
Cardiovascular disease	11 (13)	77 (87)	0·001
Diabetes mellitus	8 (17)	40 (83)	0·007
Any chronic resp. disease	2 (5)	35 (95)	1·0
COPD	1 (5)	23 (95)	1·0
Chronic kidney disease	1 (100)	0 (0)	0·06
**Body mass index (Kg/m²)**			
<25	4 (3)	147 (97)	
25-29	8 (4)	174 (96)	
30-35	12 (15)	67 (85)	
35+	6 (20)	24 (80)	<0·001
**Time to presentation (days)**	3 (3 to 4)	4 (3 to 5)	0·072

For peer review only

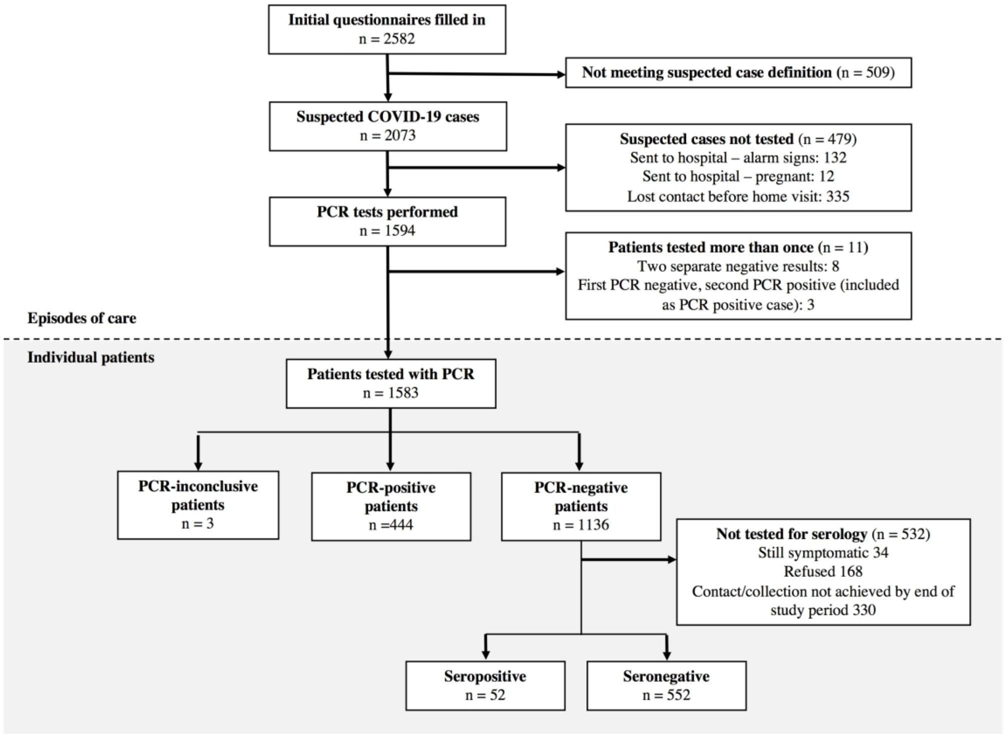

Figure 1

246x181mm (300 x 300 DPI)

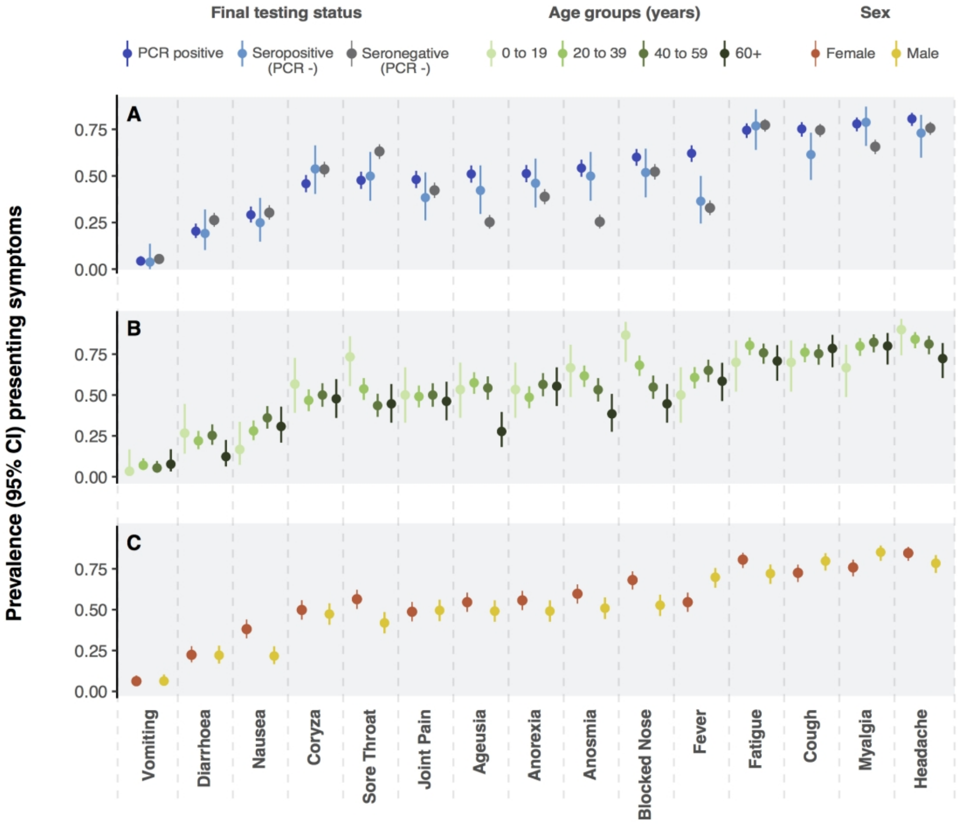

Figure 2

187x158mm (300 x 300 DPI)

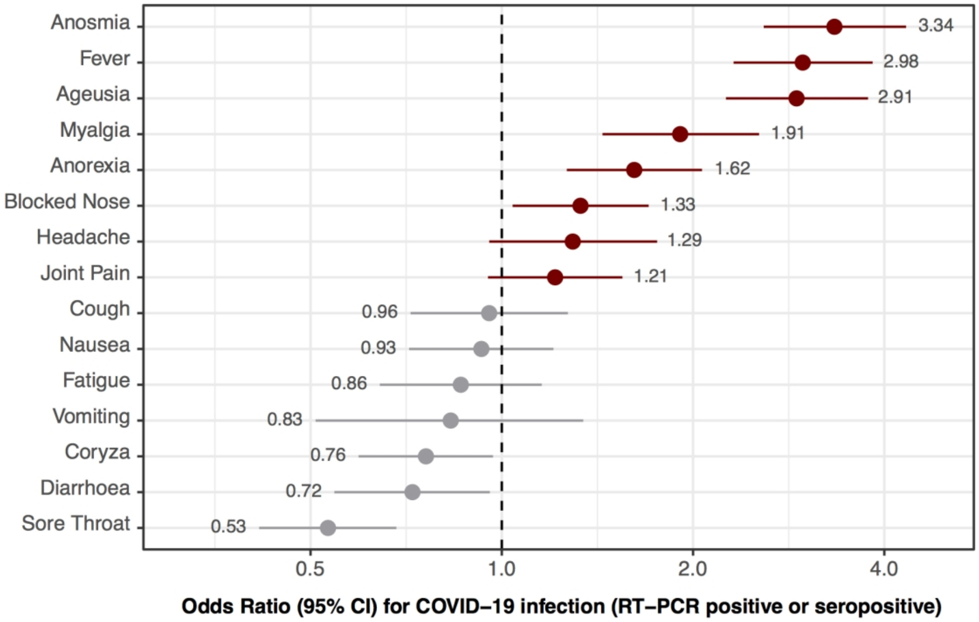

Figure 3

154x100mm (300 x 300 DPI)

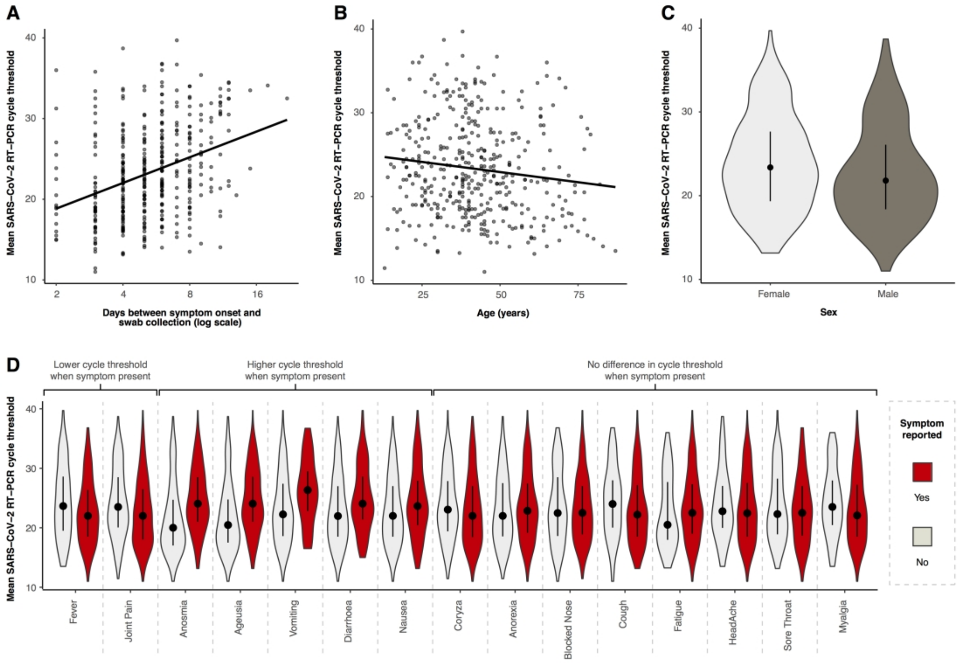

Figure 4

327x222mm (300 x 300 DPI)

Supplemental material

Contents:

1. **Initial risk assessment**
2. **Screen shots of multimedia platform and the initial questionnaire**
3. **Supplemental Table S1**
4. **Supplemental figs S1-S5**

Initial risk assessment

Patients meeting the definition of a suspected case were called by a medical student (under supervision) to complete a risk assessment. All patients were asked a set of standardized questions:

- Do you feel short of breath?
- Are you breathing quickly or finding it difficult to breath?
- If yes, can you count your respiratory rate over one minute? (respiratory rate >20 breaths/minute was considered tachypnoea)
- Has your fever worsened over the last 3 days or have you had a new fever after 2 days being fever-free?
- Have you felt confused or lethargic?

If the patient answered "yes" to any of these questions they were advised to attend a specialist health service. Among the 132 patients that were triaged to hospital, 76 (58% of 132) had shortness of breath, 76 (58% of 132) reported rapid breathing, 33 (25% of 132) persistent fever and 22 (17% of 132) altered mental status.

For peer review only

Screen shots showing examples of the initial questionnaire completed

1) Welcome page

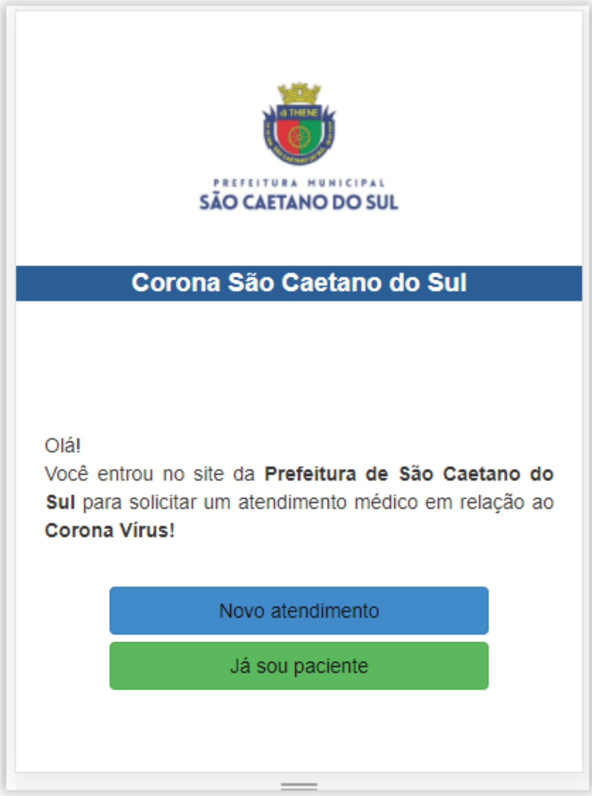

ew only

2) Zipcode confirmation

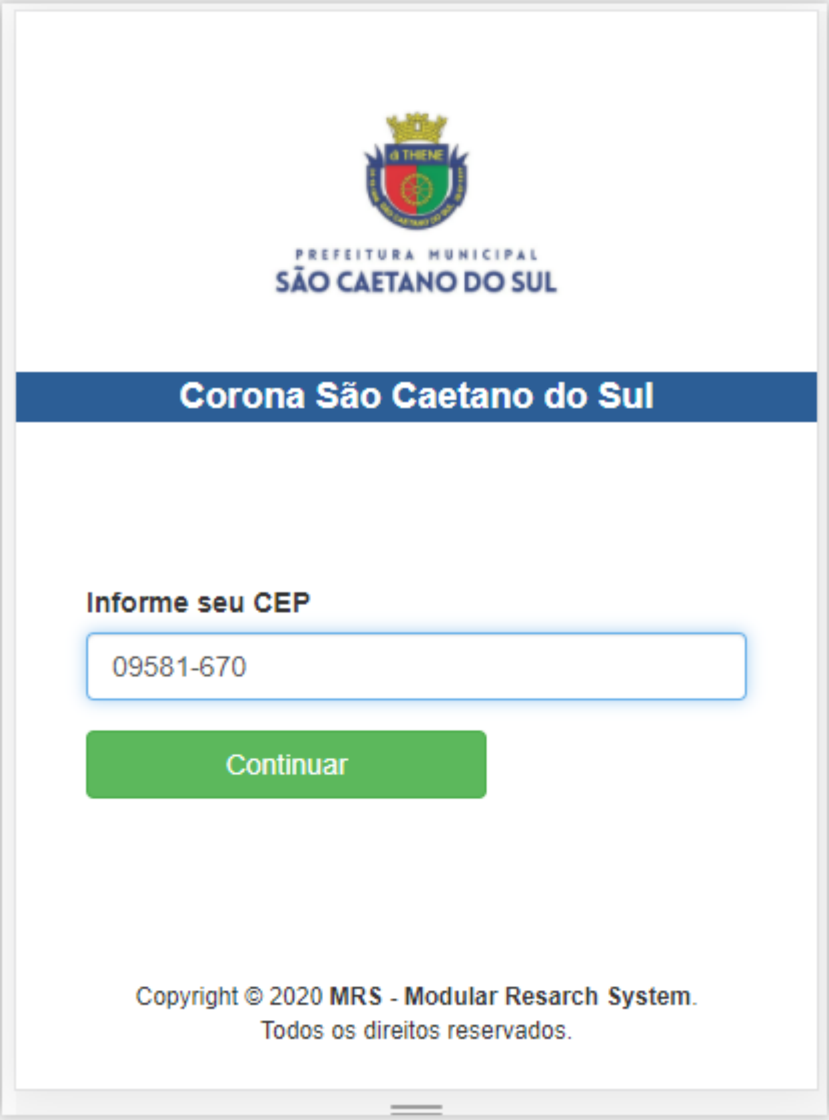

3) Patient basic information

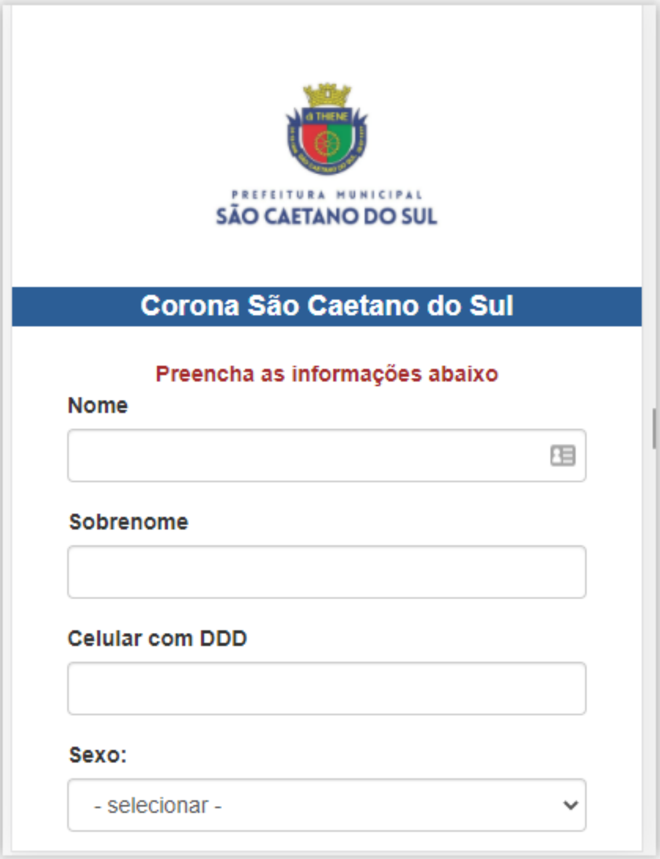

4) Access code confirmation

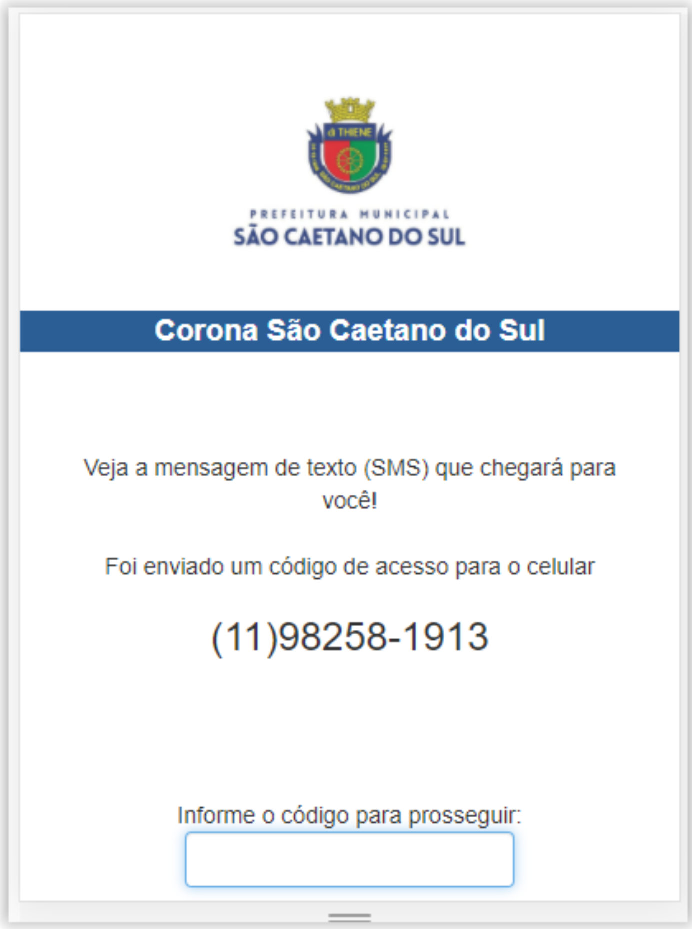

5) Questionnaire

Perguntas obrigatórias, favor responder:

Dados demográficos

Se do sexo feminino, está grávida?

- ◯ Não
- ◯ Sim
- ◯ Não se aplica

A partir do dia 1º de Março você atuou em alguma destas áreas:

- ◯ Não
- ◯ Profissional da saúde
- ◯ Áreas essenciais (segurança, bombeiro, farmácia, supermercado, transporte público)
- ◯ Cuidador (a)

Dados clínicos

Teve febre?

- ◯ Não
- ◯ Sim

Se sim, você mediu a febre?

- ◯ Não
- ◯ Sim

Se sim, qual foi a temperatura mais alta?

[ ]

Você tem tosse?

- ◯ Não
- ◯ Sim

6) Orientation page

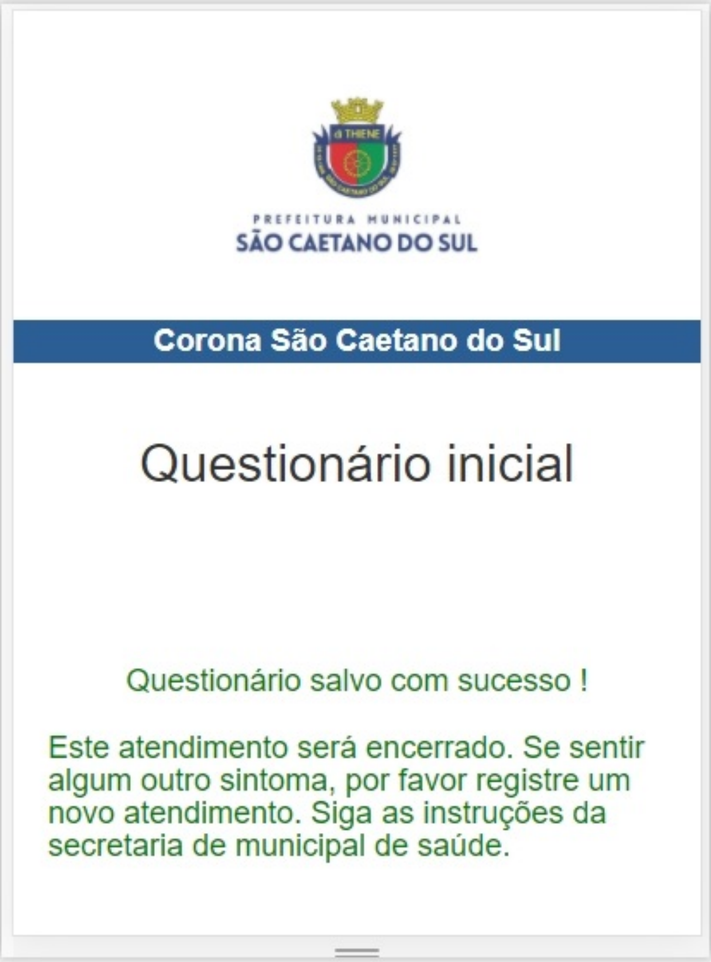

Table S1 Univariable and adjusted associations between RT-PCR cycle thresholds and patient characteristics

	**Unadjusted analysis**		**Adjusted analysis ***	
	**Beta** (difference in means)	**95% Confidence interval**	**Beta** (difference in means)	**95% Confidence interval**
**Age** (years)	**-0.05**	**-0.09 to -0.01**	**-0.06**	**-0.09 to -0.03**
**Male sex**	**-1.36**	**-2.49 to -0.23**	-1.05	-2.09 to <0.001
**Days from symptom onset to swab collection** (days, log_2)	**3.28**	**2.33 to 4.03**	**3.27**	**0.42 to 7.70**
**PCR platform** (ALTONA as reference)	**-1.19**	**-2.37 to -0.02**	**-1.53**	**-2.6 to -0.45**
**Symptoms at presentation**				
Fever	**-1.78**	**-2.96 to -0.59**	**-1.11**	**-2.11 to -0.001**
Myalgia	-1.31	-2.75 to 0.12	-0.78	-2.11 to 0.53
Arthralgia	**-1.64**	**-2.77 to -0.52**	**-1.24**	**-2.18 to -0.10**
Anosmia	**3.15**	**2.04 to 4.25**	**2.21**	**1.0 to 3.29**
Agusia	**2.99**	**1.89 to 4.09**	**1.96**	**0.88 to 3.0**
Diarrhea	**2.19**	**0.84 to 3.53**	**1.36**	**0.12 to 2.61**
Nausea	**1.50**	**0.28 to 2.72**	1.09	-0.04 to 2.24
Vomiting	**2.99**	**0.52 to 5.46**	2.02	-0.28 to 4.33
Anorexia	0.56	-0.57 to 1.70	0.47	-0.58 to 1.51
Headache	-0.58	-2.12 to 0.97	-0.81	-2.25 to 0.63
Fatigue	0.84	-0.50 to 2.18	0.34	-0.91 to 1.59
Coryza	-0.78	-1.92 to 0.34	-0.68	-1.72 to 0.34
Blocked nose	-0.36	-1.53 to 0.81	**-1.48**	**-2.59 to -0.37**
Cough	-1.33	-2.70 to 0.03	**-1.60**	**-2.86 to -0.33**
Sore throat	-0.49	-1.62 to 0.64	-0.45	-1.52 to 0.61

* All variables adjusted for age (continuous in years), sex (female as reference group), PCR platform (ALTONA platform as the reference group) and time between symptom onset and swab collection (log base 2). Analysis was performed within a linear regression framework. Positive beta coefficients indicate higher cycle thresholds (lower viral load) associated with that variable, whereas negative beta coefficients indicate lower cycle thresholds when the variable is present. Results in bold reached statistical significance.

For peer review only

Supplemental figures

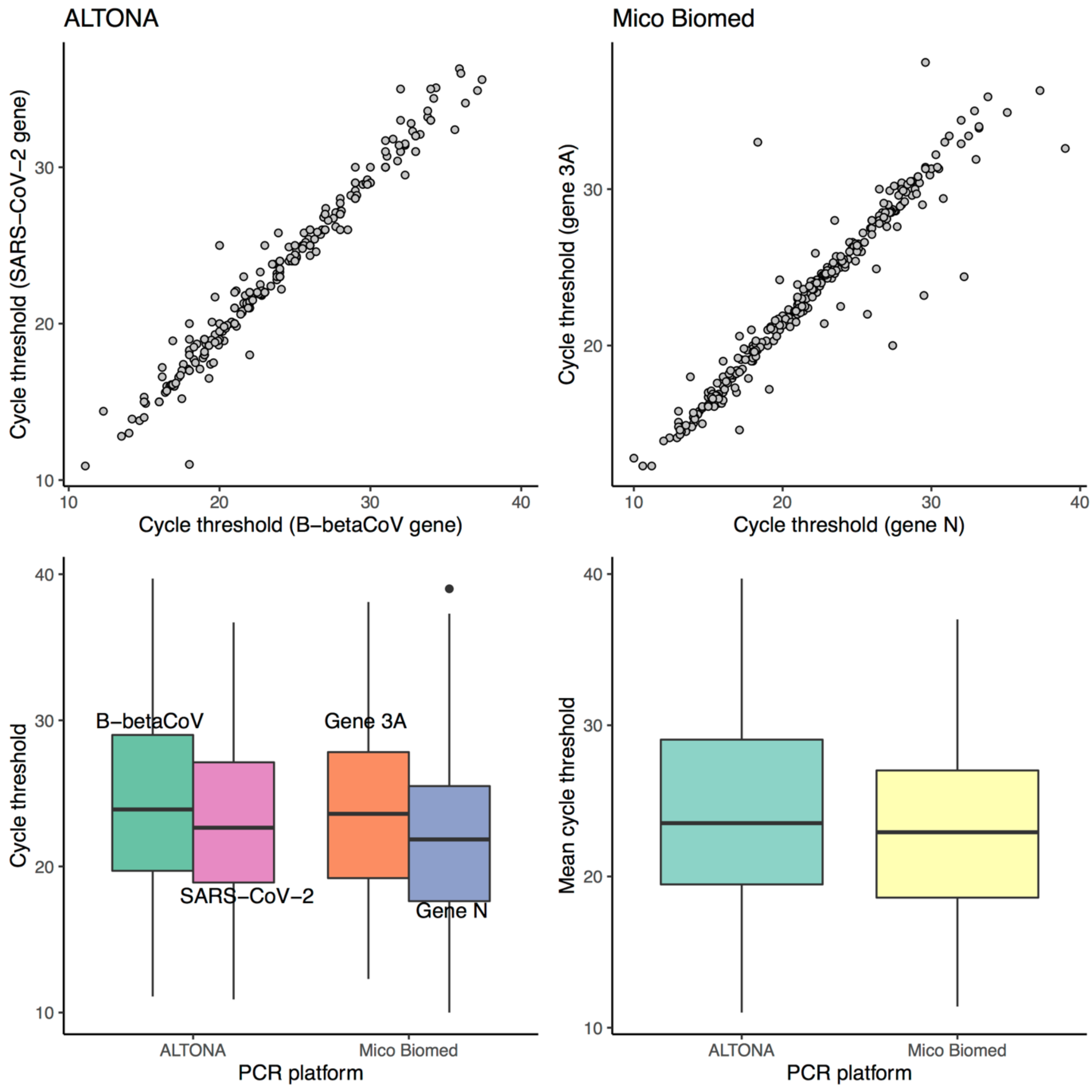

Figure S1 Comparison of cycle thresholds across PCR platforms and genes amplified. Upper two panels show the concordance between cycle thresholds for the two separate genes amplified by the ALTONA (left) and Mico Biomed (right) kits. Lower left panel – distribution of cycle thresholds by gene amplified and RT-PCR platform used. Lower right-hand panel – distribution of the mean cycle threshold (mean of cycle thresholds for separate genes) between different RT-PCR platforms.

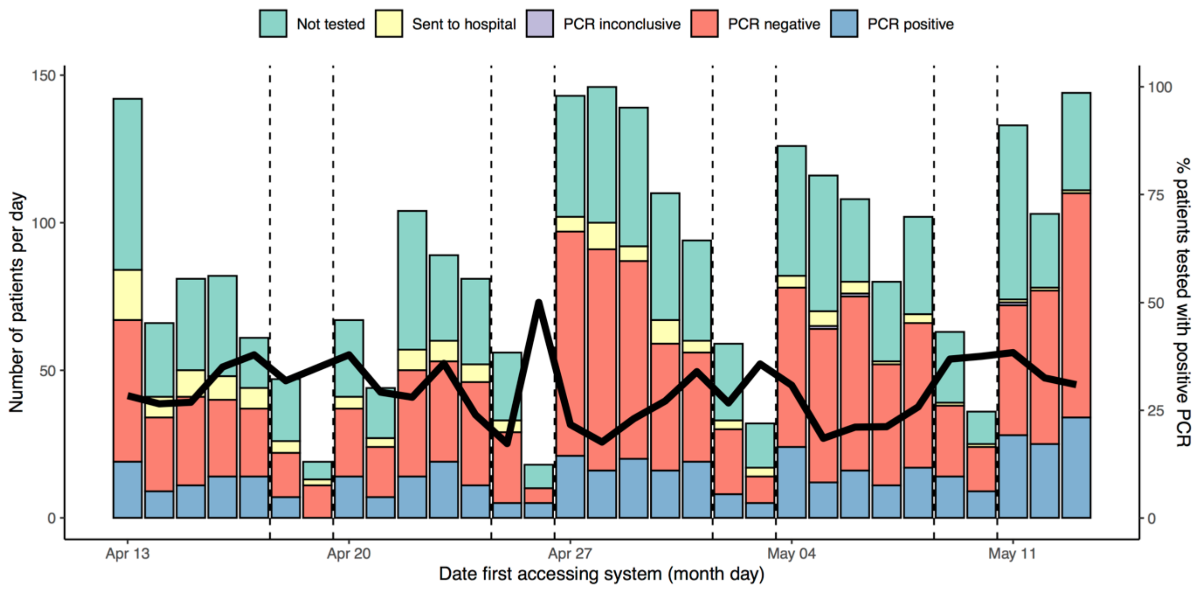

Figure S2 Time series of presentations to the Corona São Caetano platform. Dashed vertical lines denote the weekends with a reduced number of presentations. Thick black line corresponds to the right-hand y-axis: proportion of RT-PCRs performed with positive result.

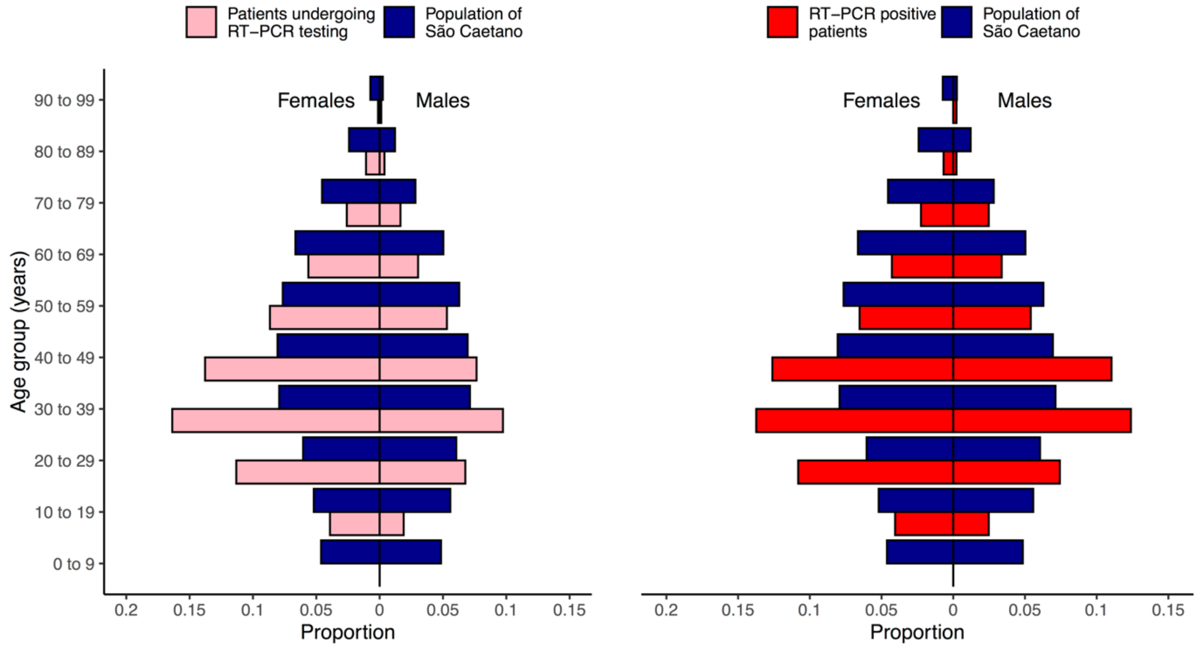

Figure S3 Age-sex distribution the city of São Caetano do Sul compared with that of patients accessing the Corona São Caetano system and being tested with RT-PCR (left-hand panel) and those testing positive for SARS-CoV-2 (right-hand panel).

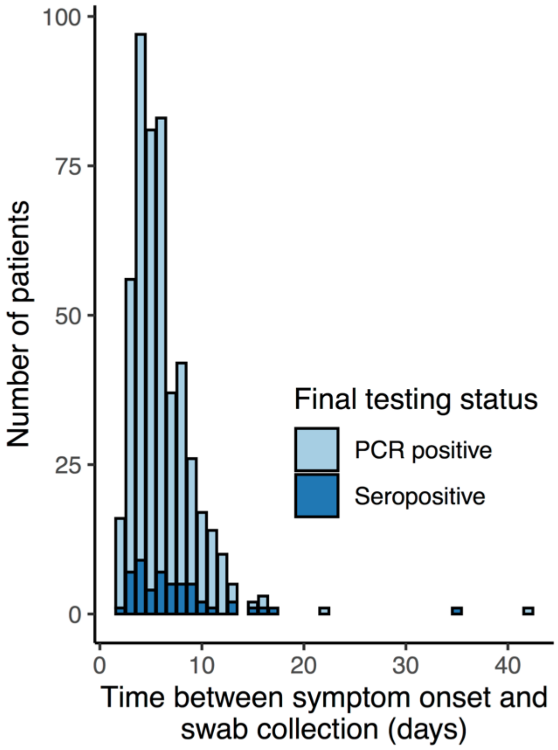

Figure S4 Histogram of delay between symptom onset and swab collection among patients with COVID-19.

For peer review only

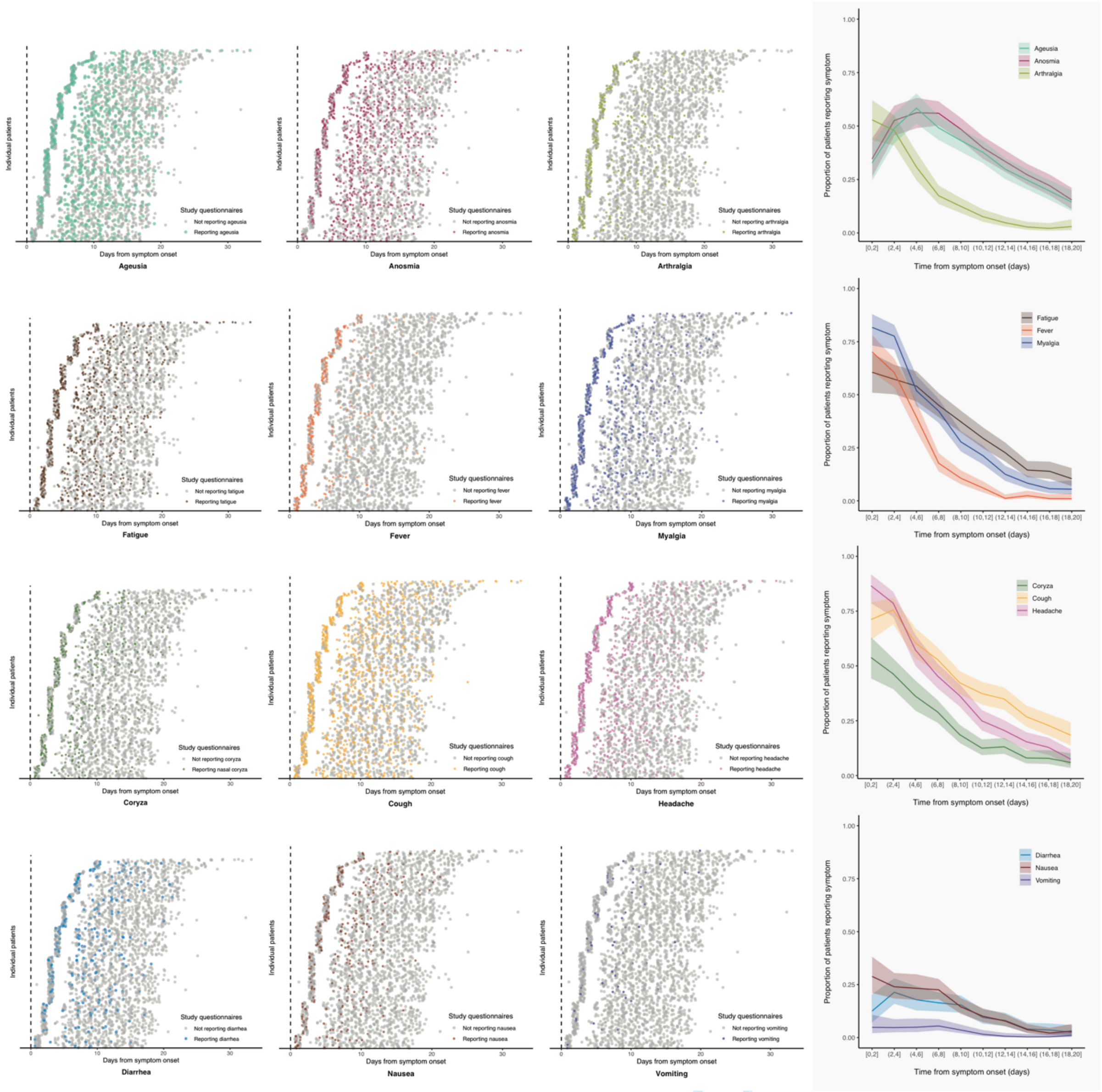

Figure S5 Left hand figures show symptoms at each follow-up questionnaire among patients testing RT-PCR positive and undergoing follow-up. Individual patients are stacked on the y-axis ordered according to the delay from symptom onset to presentation. Each point represents the response to a questionnaire and its position on the horizontal axis the time after symptom onset that the questionnaire was filled in. Grey points are questionnaires where the patient denied the presence of a given symptom. The coloured points correspond to questionnaires in which the patient reported a given symptom. The right-hand figures results from grouping the horizontal axis time into two-day windows and calculating the proportion of completed questionnaires in which each symptom was reported. The denominators for the horizontal axis groups (number of questionnaires completed within a given time window from symptom onset) are 104 at [0-2] days, 192 at (2-4], 185 at (4-6], 293 at (6-8], 338 at (8-10], 329 at (10-12], 335 at (12-14], 324 at (14-16], 280 at (16-18] and 201 at (18-20].

STROBE Statement—Checklist of items that should be included in reports of ***cohort studies***

	Item No	Recommendation	Page No
**Title and abstract**	1	(*a*) Indicate the study's design with a commonly used term in the title or the abstract<br>(*b*) Provide in the abstract an informative and balanced summary of what was done and what was found	1 and 2
**Introduction**			
Background/rationale	2	Explain the scientific background and rationale for the investigation being reported	4
Objectives	3	State specific objectives, including any prespecified hypotheses	4
**Methods**			
Study design	4	Present key elements of study design early in the paper	5 to 7
Setting	5	Describe the setting, locations, and relevant dates, including periods of recruitment, exposure, follow-up, and data collection	5 to 6
Participants	6	(*a*) Give the eligibility criteria, and the sources and methods of selection of participants. Describe methods of follow-up<br>(*b*) For matched studies, give matching criteria and number of exposed and unexposed	A – 5<br>B - NA
Variables	7	Clearly define all outcomes, exposures, predictors, potential confounders, and effect modifiers. Give diagnostic criteria, if applicable	7 to 8
Data sources/ measurement	8*	For each variable of interest, give sources of data and details of methods of assessment (measurement). Describe comparability of assessment methods if there is more than one group	5 to 7
Bias	9	Describe any efforts to address potential sources of bias	7 to 8
Study size	10	Explain how the study size was arrived at	8
Quantitative variables	11	Explain how quantitative variables were handled in the analyses. If applicable, describe which groupings were chosen and why	
Statistical methods	12	(*a*) Describe all statistical methods, including those used to control for confounding<br>(*b*) Describe any methods used to examine subgroups and interactions<br>(*c*) Explain how missing data were addressed<br>(*d*) If applicable, explain how loss to follow-up was addressed<br>(*e*) Describe any sensitivity analyses	A – 7 to 8<br>B – NA<br>C – 8<br>D – NA<br>E – NA
**Results**			
Participants	13*	(a) Report numbers of individuals at each stage of study—eg numbers potentially eligible, examined for eligibility, confirmed eligible, included in the study, completing follow-up, and analysed<br>(b) Give reasons for non-participation at each stage<br>(c) Consider use of a flow diagram	Figure 1 and page 9
Descriptive data	14*	(a) Give characteristics of study participants (eg demographic, clinical, social) and information on exposures and potential confounders<br>(b) Indicate number of participants with missing data for each variable of interest	A – table 1 and pages 9to10<br>B - Table 1 and 2 legends

For peer review only - http://bmjopen.bmj.com/site/about/guidelines.xhtml

		(c) Summarise follow-up time (eg, average and total amount)	C – page 9
Outcome data	15*	Report numbers of outcome events or summary measures over time	11
Main results	16	(*a*) Give unadjusted estimates and, if applicable, confounder-adjusted estimates and their precision (eg, 95% confidence interval). Make clear which confounders were adjusted for and why they were included<br>(*b*) Report category boundaries when continuous variables were categorized<br>(*c*) If relevant, consider translating estimates of relative risk into absolute risk for a meaningful time period	Page 7 and results section
Other analyses	17	Report other analyses done—eg analyses of subgroups and interactions, and sensitivity analyses	NA
**Discussion**			
Key results	18	Summarise key results with reference to study objectives	12
Limitations	19	Discuss limitations of the study, taking into account sources of potential bias or imprecision. Discuss both direction and magnitude of any potential bias	13
Interpretation	20	Give a cautious overall interpretation of results considering objectives, limitations, multiplicity of analyses, results from similar studies, and other relevant evidence	13
Generalisability	21	Discuss the generalisability (external validity) of the study results	13
**Other information**			
Funding	22	Give the source of funding and the role of the funders for the present study and, if applicable, for the original study on which the present article is based	14

*Give information separately for exposed and unexposed groups.

Note: An Explanation and Elaboration article discusses each checklist item and gives methodological background and published examples of transparent reporting. The STROBE checklist is best used in conjunction with this article (freely available on the Web sites of PLoS Medicine at http://www.plosmedicine.org/, Annals of Internal Medicine at http://www.annals.org/, and Epidemiology at http://www.epidem.com/). Information on the STROBE Initiative is available at http://www.strobe-statement.org.

For peer review only

BMJ Open

Clinical features and natural history of the first 2,073 suspected COVID-19 cases in the Corona São Caetano primary care programme: a prospective cohort study

Journal:	*BMJ Open*
Manuscript ID	bmjopen-2020-042745.R3
Article Type:	Original research
Date Submitted by the Author:	04-Dec-2020
Complete List of Authors:	Leal, Fabio ; Universidade Municipal de São Caetano do Sul; Instituto Nacional de Cancer<br>Mendes-Correa, Maria; Universidade de São Paulo, Instituto de Medicina Tropical (LIM-52, LIM-46, LIM-49) and Departamento de Moléstias Infecciosas e Parasitarias<br>Buss, Lewis; Universidade de São Paulo, Instituto de Medicina Tropical (LIM-52, LIM-46, LIM-49) and Departamento de Moléstias Infecciosas e Parasitarias<br>Costa, Silvia; Universidade de São Paulo, Instituto de Medicina Tropical (LIM-52, LIM-46, LIM-49) and Departamento de Moléstias Infecciosas e Parasitarias<br>Bizario, Joao; Universidade Municipal de São Caetano do Sul<br>de Souza, Sonia ; Universidade Municipal de São Caetano do Sul<br>Thomaz, Osorio; Instituto de Pesquisas Tecnológicas<br>Tozetto-Mendoza, Tania; Universidade de São Paulo, Instituto de Medicina Tropical (LIM-52, LIM-46, LIM-49) and Departamento de Moléstias Infecciosas e Parasitarias<br>Villas-Boas, Lucy; Universidade de São Paulo, Instituto de Medicina Tropical (LIM-52, LIM-46, LIM-49) and Departamento de Moléstias Infecciosas e Parasitarias<br>Oliveira, Lea; Universidade de São Paulo, Instituto de Medicina Tropical (LIM-52, LIM-46, LIM-49) and Departamento de Moléstias Infecciosas e Parasitarias<br>Grespan, Regina; Universidade Municipal de São Caetano do Sul<br>Capuani, Ligia; Modular Research System<br>Buccheri, Renata; Universidade de São Paulo, Instituto de Medicina Tropical (LIM-52, LIM-46, LIM-49) and Departamento de Moléstias Infecciosas e Parasitarias<br>Domingues, Helves; Modular Research System<br>Alexander, Neal; London School of Hygiene & Tropical Medicine<br>Mayaud, Philippe; London School of Hygiene & Tropical Medicine<br>Sabino, Ester; Universidade de São Paulo, Instituto de Medicina Tropical (LIM-52, LIM-46, LIM-49) and Departamento de Moléstias Infecciosas e Parasitarias
**Primary Subject Heading**:	Infectious diseases
Secondary Subject Heading:	Epidemiology, Public health, Global health

Keywords:	PRIMARY CARE, Epidemiology < TROPICAL MEDICINE, INFECTIOUS DISEASES, PUBLIC HEALTH

SCHOLARONE™ Manuscripts

I, the Submitting Author has the right to grant and does grant on behalf of all authors of the Work (as defined in the below author licence), an exclusive licence and/or a non-exclusive licence for contributions from authors who are: i) UK Crown employees; ii) where BMJ has agreed a CC-BY licence shall apply, and/or iii) in accordance with the terms applicable for US Federal Government officers or employees acting as part of their official duties; on a worldwide, perpetual, irrevocable, royalty-free basis to BMJ Publishing Group Ltd ("BMJ") its licensees and where the relevant Journal is co-owned by BMJ to the co-owners of the Journal, to publish the Work in this journal and any other BMJ products and to exploit all rights, as set out in our licence.

The Submitting Author accepts and understands that any supply made under these terms is made by BMJ to the Submitting Author unless you are acting as an employee on behalf of your employer or a postgraduate student of an affiliated institution which is paying any applicable article publishing charge ("APC") for Open Access articles. Where the Submitting Author wishes to make the Work available on an Open Access basis (and intends to pay the relevant APC), the terms of reuse of such Open Access shall be governed by a Creative Commons licence – details of these licences and which Creative Commons licence will apply to this Work are set out in our licence referred to above.

Other than as permitted in any relevant BMJ Author's Self Archiving Policies, I confirm this Work has not been accepted for publication elsewhere, is not being considered for publication elsewhere and does not duplicate material already published. I confirm all authors consent to publication of this Work and authorise the granting of this licence.

For peer review only

Clinical features and natural history of the first 2,073 suspected COVID-19 cases in the Corona São Caetano primary care programme: a prospective cohort study

Fabio E Leal[1,2]* (PhD), Maria C Mendes-Correa[3]* (PhD), Lewis F Buss[3]*# (MBBS), Silvia F Costa[3] (PhD), Joao CS Bizario[1] (PhD), Sonia RP de Souza[1] (PhD), Osorio Thomaz[4] (PhD), Tania R Tozetto-Mendoza[1] (PhD), Lucy S Villas-Boas[3], Lea CO Silva[3] (PhD), Regina MZ Grespan (MD)[1], Ligia Capuani[5] (PhD), Renata Buccheri[3] (MD), Helves Domingues (PhD)[5], Neal DE Alexander[6] (PhD), Philippe Mayaud[6] (MD), Ester C Sabino[3] (PhD)

* These authors contributed equally

Affiliations

1. Universidade Municipal de São Caetano do Sul (USCS), Rua Santo Antonio, São Caetano do Sul, Brazil
2. National Cancer Institute (INCA), Praça Cruz Vermelha, Rio de Janeiro, Brazil
3. Instituto de Medicina Tropical (LIM-52, LIM-46, LIM-49) and Departamento de Moléstias Infecciosas e Parasitarias da Faculdade de Medicina da Universidade de São Paulo Avenida Dr. Enéas Carvalho de Aguiar, São Paulo, Brazil
4. Instituto de Pesquisas Tecnológicas do Estado de São Paulo - IPT
5. Modular Research System, Avenida Paulista, São Paulo, Brazil
6. London School of Hygiene and Tropical Medicine (LSHTM), Keppel Street, London, United Kingdom

Corresponding author:

Dr Lewis F Buss

Instituto de Medicina Tropical da Faculdade de Medicina da Universidade de São Paulo

lewisbuss@usp.br

KEY WORDS: SARS-CoV-2, COVID-19, pandemic, community, primary care, Brazil

For peer review only

ABSTRACT

Background: Despite most cases not requiring hospital care, there are limited community-based clinical data on COVID-19.

Methods: The Corona São Caetano program is a primary care initiative providing care to all residents with COVID-19 in São Caetano do Sul, Brazil. It was designed to capture standardised clinical data on community COVID-19 cases. After triage of potentially severe cases, consecutive patients presenting to a multimedia screening platform between 13th April and 13th May 2020 were tested at home with SARS-CoV-2 reverse transcriptase (RT) PCR; positive patients were followed up for 14 days with phone calls every 2 days. RT-PCR-negative patients were offered additional SARS-CoV-2 serology testing to establish their infection status. We describe the clinical, virologic and natural history features of this prospective population-based cohort.

Findings: Of 2,073 suspected COVID-19 cases, 1,583 (76.4%) were tested by RT-PCR, of whom 444 (28.0%, 95%CI: 25.9-30.3) were positive; 604/1,136 (53%) RT-PCR-negative patients underwent serology, of whom 52 (8.6%) tested SARS-CoV-2 seropositive. The most common symptoms of confirmed COVID-19 were cough, fatigue, myalgia and headache; whereas self-reported fever (OR 3.0, 95%CI: 2.4-3.9), anosmia (OR 3.3, 95%CI: 2.6-4.4), and ageusia (2.9, 95%CI: 2.3-3.8) were most strongly associated with a positive COVID-19 diagnosis by RT-PCR or serology. RT-PCR cycle thresholds were lower in men, older patients, those with fever and arthralgia, and closer to symptom onset. The rates of hospitalization and death among 444 RT-PCR-positive cases were 6.7% and 0.7%, respectively, with older age and obesity more frequent in the hospitalized group.

Conclusion: COVID-19 presents in a similar way to other mild community-acquired respiratory diseases, but the presence of fever, anosmia, and ageusia can assist the specific diagnosis. Most patients recovered without requiring hospitalization with a low fatality rate compared to other hospital-based studies.

For peer review only - http://bmjopen.bmj.com/site/about/guidelines.xhtml

Strengths and limitations of this study

1. The clinical features of COVID-19 have mostly been described in hospital-based studies which are biased towards severe disease
2. We report a prospective cohort of suspected and confirmed COVID-19 cases from a primary care initiative in the Brazilian municipality of São Caetano do Sul
3. By systematically testing consecutive suspected community cases with molecular and serological tests we were able to address the diagnostic value of clinical features of mild-moderate COVID-19 in primary care
4. Prospective follow-up of confirmed cases and linkage with hospital datasets allowed us to describe the natural history of a primary care COVID-19 population
5. A limitation of the work was that not all PCR-negative participants underwent serology testing due to loss to follow-up

For peer review only

For peer review only - http://bmjopen.bmj.com/site/about/guidelines.xhtml

INTRODUCTION

A comprehensive public health response is vital but difficult to achieve during an epidemic. The COVID-19 pandemic, caused by the novel severe acute respiratory syndrome coronavirus 2 (SARS-CoV-2), started in China in late 2019.[1] According to the World Health Organization (WHO)[2,3] and others[4,5], the ideal early response should have been multipronged, with identification, isolation, treatment and contact tracing of symptomatic cases, relying on a strong testing programme. Primary health care is well placed to implement such a response, by identifying cases early and managing them in a way that minimizes overcrowding of emergency rooms and intensive care units.[6,7] Real-time data analysis coming from these primary care response systems can inform policy decisions.

Primary health care (PHC) in Brazil is provided by the publicly funded Unified Health System (SUS – Portuguese acronym) within the family health strategy (*Estratégia Saúde da Família*). Provision of care is centred around a healthcare unit with a multi-professional team that is responsible for all residents in the immediate catchment area[8]. Nearly two-thirds of the Brazilian population is covered by the family health strategy[8].

In Brazil, the first case of COVID-19 was identified in the city of São Paulo on 26th February 2020.[9] As of 1st Dec 2020 there were over 6 million confirmed cases nationally, with São Paulo contributing a fifth of these.[10] The reasons for the exceptionally large epidemic in Brazil have been discussed elsewhere[11–13]. In March 2020, the Municipal Health Department of the municipality of São Caetano do Sul – part of the Greater Metropolitan Region of São Paulo – began to develop a clinical and testing platform to organize its COVID-19 response. The aim was to provide universal detection and management of symptomatic cases and their contacts. The platform was developed in partnership with two local universities – the Municipal University of São Caetano do Sul (USCS) and the University of Sao Paulo (USP) – and called “Corona São Caetano”.

Large scale community-based observational cohorts are difficult to establish under epidemic circumstances, particularly if the risk of exposure for research personnel is high. Hence, most COVID-19 epidemiological and clinical studies have been hospital-based,[14–16] and therefore tend to include more severe cases whose findings may not be generalizable to the general

For peer review only - http://bmjopen.bmj.com/site/about/guidelines.xhtml

population[17], although some limited descriptions from ambulatory settings are available[18–20].

The objectives of this study were to describe the epidemiological indicators of the early phase of the programme rollout; and to describe the clinical, virological and natural history features (including hospitalization and deaths) of SARS-CoV-2 infection among patients identified in primary care.

METHODS

Setting

The municipality of São Caetano do Sul has a population of 161,000 inhabitants.[21] The city's population is older than the Brazilian population[21] and its Human Development Index is one of the highest in the country. Nearly all (97.4%) children aged 6-14 are in education and 31% of the population have completed higher education[22] (Brazilian national average is 11%).

Corona São Caetano platform

The objective of the platform was to offer clinical care for patients with flu syndrome and suspected COVID-19. Through the multimedia platform (website or phone call), patients could be triaged and guided in relation to their clinical needs and tested, without having to leave their homes or go to health facilities, unless seriously ill. This strategy aimed at reducing the workload in health units and the risk of SARS-CoV-2 transmission in the population served by these health units. Patients' GPs were informed of lab results and had access to clinical data stored in the platform. GPs were expected to call patients being assisted by the platform and provide medical assistance through home visits or at the primary care clinic if needed. In general, the drugs prescribed through the platform were restricted to analgesics and antipyretics. The platform was designed so that clinical information was collected in a standardized way for research purposes.

Residents of the municipality aged 12 years and older with suspected COVID-19 symptoms were encouraged, through local media reports, to contact the dedicated Corona São Caetano platform via the website or by phone. They were invited to complete an initial screening

For peer review only

questionnaire that included socio-demographic data; information on symptoms type, onset and duration; and recent contacts.

Patients meeting the suspected COVID-19 case definition (i.e., having at least two of the following symptoms: fever, cough, sore throat, coryza, or change in/loss of smell (anosmia); or one of these symptoms plus at least two other symptoms consistent with COVID-19) were further evaluated, whilst people not meeting these criteria were reassured, advised to stay at home and contact the service again if they were to develop new symptoms or worsening of current ones. The case definition was developed in consultation with infectious disease and primary care specialists to encompass the known symptoms of COVID-19 and is similar to the Brazilian national case definition[23]. Patients were then called by a medical student to complete a risk assessment. All pregnant women, and patients meeting pre-defined triage criteria for severe disease (see Supplemental Material), were advised to attend a hospital service - either an emergency department or outpatient service, depending on availability. All other patients were offered a home visit for self-collection of a nasopharyngeal swab.

Sample collection

Patients self-collected nasopharyngeal swabs (NPS – both nostrils and throat) at their own homes under the supervision of trained healthcare personnel. We sent a link to an instructional video (https://youtu.be/rWZzV2ZP7KY) before the home visit to provide guidance on self-collection procedures. Nasopharyngeal swabs for the molecular detection of SARS-CoV-2 has been recommended as an alternative method of collection for samples from patients with suspected COVID-19[24], as well as other respiratory diseases, and has the advantage of reducing the chance of aerosol transmission to healthcare professionals. Healthcare personnel were instructed to maintain a distance of six feet from the patient and to wear personal protective equipment at all times. Samples were immediately put on a cool box between 2-8°C and stored at 4°C in a fridge until shipment to the lab within 24 hours.

Follow-up procedures

Patients testing SARS-CoV-2 RT-PCR positive were followed up to 14 days[25] (a maximum of 7 phone calls) from completion of their initial questionnaire. They were contacted every 48 hours by a medical student who completed another risk assessment and recorded any ongoing

For peer review only - http://bmjopen.bmj.com/site/about/guidelines.xhtml

or new symptoms. The purpose of the follow-up was to assess clinical evolution. Where patients were judged to be deteriorating or developing severe disease they were signposted to secondary care services. Patients testing RT-PCR negative were followed up by the primary health care program for their residential area. They were advised to contact the platform for a new consultation if they developed new symptoms. Starting on May 19th, when serological testing became available, RT-PCR-negative patients were re-contacted to offer antibody (IgG/IgM combined) testing 14 days after their initial registration as long as they had become asymptomatic.

Study dates

The Corona São Caetano programme was launched on 6th April 2020, with a one week pilot phase designed to test instruments before roll-out. For this analysis, we included all patients making their first contact with the programme in its first month, ie between 13th April and 13th May 2020. The period of follow-up (last date of data extraction) was 4th June 2020, to account for the accrual period (three weeks) of possible hospitalizations in the last included patients.

Laboratory methods

Due to shortages of some reagents, we used two RT-PCR platforms at different times during the study: ALTONA RealStar® SARS-CoV-2 RT-PCR Kit 1.0 (Hamburg, Germany) and the Mico BioMed RT-qPCR kit (Seongnam, South Korea). For serology we tested 10μL of serum or plasma (equivalent in performance) using a qualitative rapid chromatographic immunoassay (Wondfo Biotech Co., Guangzhou, China), that jointly detects anti-SARS-CoV-2 IgG/IgM. The assay has been found to have a sensitivity of 81.5% and specificity of 99.1% in a US study[26]. In our local validation, after two weeks of symptoms, the sensitivity in 59 RT-PCR confirmed cases was 94.9%, and specificity in 106 biobank samples from 2019 was 100%.

Statistical methods

We estimated the contribution of our platform to total number of COVID-19 cases diagnosed in São Caetano do Sul. To do this, we compared the number of cases diagnosed in our

For peer review only - http://bmjopen.bmj.com/site/about/guidelines.xhtml

programme with official data released by the Municipal Department of Health in its daily bulletins (accessed here https://coronavirus.saocaetanodosul.sp.gov.br).

Clinical and demographic data were extracted directly from the Corona São Caetano information system. To analyse clinical presentation, we first calculated the proportion and exact binomial 95% confidence intervals (CI) of cases reporting each symptom in the three testing groups: SARS-CoV-2 RT-PCR positive; RT-PCR negative / seropositive; and RT-PCR negative / seronegative. We next combined RT-PCR and serology positive cases to make a confirmed COVID-19 group, and those negative on both tests to make a SARS-CoV-2 negative control group. We express the association between each symptom and a positive COVID-19 diagnosis as odds ratios (OR) and 95% CIs.

Next, we assessed associations between RT-PCR cycle thresholds (Cts) and other clinical features. ALTONA and MiCo BioMed RT-PCR kits each separately amplify two different SARS-CoV-2 viral genes, as such each patient had two Ct values. There was a high concordance between Cts for the two genes within each kit (Figure S1), and we opted therefore to use the mean of the two Ct values for each patient in all analyses. We calculated univariable associations between Cts and age, sex, delay from symptom onset to NPS collection, and presenting symptoms using simple linear regression. We then built a multivariable linear regression model to assess independent associations between presenting symptoms and RT-PCR Cts. As age, sex, and time of swab collection may confound this relationship we included these variables, as well as the RT-PCR platform (ALTONA vs MiCo BioMed), as covariates in the model.

For RT-PCR positive patients, hospitalizations and deaths were extracted from the study platform. To extend the follow-up period and to capture RT-PCR negative patients and those initially triaged to hospital (no study follow-up), hospitalization and vital status was confirmed by linkage with two administrative databases: the municipal epidemiological surveillance dataset, as well as the state-wide influenza-like illness notification system (SIVEP-Gripe). Linkage was last performed on 5th June 2020, 23 days after the last patient was enrolled, by the author SRPS who did not have access to the full analytic dataset. This author searched the SIVEP-Gripe system and the municipal epidemiological surveillance dataset using full name and date of birth. Categorical patient characteristics were compared between patients requiring and those not requiring hospitalization using a Chi-squared or

For peer review only - http://bmjopen.bmj.com/site/about/guidelines.xhtml

Fisher exact test. Continuous variables were compared using the Wilcoxon rank sum test. A multivariate analysis was not conducted due to the small number of individuals experiencing this outcome.

The cohort sample included consecutive cases presenting to the Corona São Caetano program and a formal sample size calculation was not performed. Missing data were excluded. All analyses were conducted in R Software for Statistical Computing, version 3.6.3.[27]

Ethics

The study was approved by the local ethics committee (Comissão de Ética para Análise de Projeto de Pesquisa - CAPPesq, protocol No. 13915, dated June 03, 2020). The committee waived the need for informed consent and allowed the development of an analytical dataset with no personal identification for the current analysis.

Patient and public involvement

Patients were not involved in the planning of this research.

RESULTS

Epidemiological and programmatic indicators

Over the study period, there were 2,073 presentations (49% phone call, 51% website), from 2,011 individual patients, that met the criteria for a suspected COVID-19 case (See Figure 1 for study flow). At initial phone interview, 132 (6%) potential cases were advised to go directly to a health service based on the triage questions, and 12 (0.6%) because of pregnancy. Only four (3%) of referred patients were admitted to hospital and none died.

In total 1,583 individual patients were tested with RT-PCR for SARS-CoV-2; 444 (28.0%, 95%CI: 25.9-30.3) were positive. The proportion of positive results was stable over the study (Figure S2). Among the RT-PCR negative group, 604 (53% of 1,136) underwent serology testing, of whom 52 (8.6%, 95%CI: 6.6-11.1) were seropositive. The median [IQR] time from symptom onset to serology collection was 31 [26–37] days. The age-sex structure of patients

For peer review only - http://bmjopen.bmj.com/site/about/guidelines.xhtml

being tested differed from the underlying population of São Caetano do Sul (Figure S3) with an overrepresentation of working-age adults and women. At the beginning of programme role out, 25% of notified COVID-19 cases in São Caetano do Sul were diagnosed in our programme. Over the study period, adherence to the programme increased, and by May 13th, 2020, this figure had risen to 78%.

Of 444 RT-PCR positive patients eligible for longitudinal follow-up, 326 (73%) had their final follow-up visit at least 14 days after their initial presentation. Of the seven possible follow-up questionnaires, 384 (86%) COVID-19 patients completed three or more, and 162 (36%) completed all seven.

Participant characteristics

Patient characteristics are shown in Table 1. Although women were overrepresented in the cohort, there were proportionally more males in the RT-PCR positive and seropositive groups compared to the seronegative group. Of note, 55% of RT-PCR negative/seronegative patients had completed higher education compared to 35% RT-PCR-positive patients ($p <0.001$, Chi-squared test). The median number of days from symptom onset to swab collection was 5.0 (IQR, 4.0-7.0) among RT-PCR positive patients and 6.0 (IQR, 4.0-8.3) among RT-PCR negative/seropositive patients ($p = 0.06$, Wilcoxon rank sum) (Figure S4). Chronic respiratory disease was less frequent in RT-PCR positive than dual-negative patients.

Symptoms of COVID-19

The prevalence of individual symptoms at presentation is shown in Figure 2A stratified by final diagnostic category. The most frequent symptoms among RT-PCR and seropositive patients were headache (82% and 75%), myalgia (80% and 80%), cough (77% and 63%), and fatigue (77% and 79%). Anosmia was present in 56% and 63% of RT-PCR positive and seropositive patients, respectively, compared to 30% in those testing doubly negative. A similar pattern was observed for ageusia (53% and 53% versus 30%). Upper respiratory tract symptoms - including coryza, blocked nose, ageusia, and anosmia - were more frequent in younger people (Figure 2B). The evolution of symptoms over time among RT-PCR positive patients is shown in Figure S5.

For peer review only - http://bmjopen.bmj.com/site/about/guidelines.xhtml

The odds ratios for testing positive for SARS-CoV-2 (RT-PCR or serology) associated with each presenting symptom are shown in Figure 3. The symptoms with strongest associations were anosmia (OR 3.3, 95%CI: 2.6-4.4), fever (3.0, 95%CI: 2.4-3.9) and ageusia (2.9, 95%CI: 2.3-3.8). The presence of sore throat (0.53, 95%CI: 0.41-0.68) and diarrhoea (0.72, 95%CI: 0.55-0.96) were associated with a negative SARS-CoV-2 test.

Associations between SARS-CoV-2 RT-PCR Cycle threshold (Ct) values, and demographic and clinical features

Figure 4 shows the associations between mean RT-PCR cycle threshold and demographic features and symptoms at presentation (the median [IQR] time from presentation to swab was 1 [1-2] day). Older age was associated with lower cycle thresholds, with a change in mean Ct of -0.05 (95%CI -0.09 to -0.01) for each additional year of age. The mean difference in Ct value was -1.36 (95% CI -2.49 to -0.23) in men compared to women. For each doubling in the number of days from symptom onset to swab collection the mean Ct value increased by 3.28 (95%CI 2.33 to 4.03). Presenting symptoms of fever and arthralgia were associated with lower Cts, whereas anosmia, ageusia, vomiting, diarrhoea, and nausea were associated with higher Cts (Figure 4 and Table S1). After adjustment for age, sex, delay from symptom onset, and RT-PCR platform used, fever (-0.06, 95%CI: -2.11 to -0.001) and arthralgia (-1.24, 95%CI: -2.18 to -0.10) remained associated with lower Cts, and anosmia (2.21, 95%CI: 1.0-3.29), ageusia (1.96, 95%CI: 0.88-3.0), and diarrhoea (1.36, 95%CI: 0.12-2.61) with higher Cts (Table S1).

Hospitalizations and deaths

Of the 444 RT-PCR positive patients, 30 (6.8%) had been hospitalized by 5th June 2020, when the database linkage was last updated, and three (0.7%) had died; in-hospital mortality was therefore 10% (3/30). In 28 cases the date of admission was available. The median time from symptom onset to hospital admission was 7 (range 2 to 14) days. Among 1,136 RT-PCR-negative patients, six (0.5%) had been admitted to hospital. One (<0.01% of 1,136) of these six patients died. None of the 604 RT-PCR negative patients that underwent serology were admitted to hospital or died. Table 2 compares patient characteristics by hospitalization status. Notably, hospitalized patients were older, had more cardiovascular comorbidities and were more frequently obese.

For peer review only - http://bmjopen.bmj.com/site/about/guidelines.xhtml

DISCUSSION

We present a community-based cohort of suspected COVID-19 cases recruited through a primary care initiative in the Brazilian municipality of São Caetano do Sul. Offering RT-PCR testing to all patients presenting with symptoms compatible with COVID-19, the positivity rate was 28%, with 8.6% of those testing negative subsequently found to be seropositive - i.e. > 35% of the cohort had a diagnosis of COVID-19. Anosmia, ageusia, and self-reported fever provided the greatest diagnostic value in identifying COVID-19. The rate of hospitalization and deaths among RT-PCR positive patients was low, at 6.8% and 0.7%, respectively. Our results provide important information on the clinical presentation, diagnostic testing and natural history of COVID-19 identified in the community.

The profile of suspected cases that tested positive for COVID-19 differed in some important respects from those testing negative. The lower educational level among positive cases suggests that, in São Caetano do Sul, the risk of exposure to COVID-19 follows a socioeconomic gradient, consistent with other findings from Brazil[13,28]. Although more women presented to the platform, proportionally more men tested positive, consistent with data from São Paulo showing a higher seroprevalence in men than women[11], but also potentially reflecting different health seeking behaviours. Comorbidities were mostly similar, although chronic respiratory disease was less frequent in those testing RT-PCR positive. This may be due to a proportion of presentations in those with chronic respiratory disease being explained by exacerbations of their underlying pathology from aetiologies other than SARS-CoV-2, as well as higher anxiety about COVID-19 in those with pre-existing respiratory disease.

Extrapolating the seropositivity rate among RT-PCR negative patients to the 532 who were not tested with serology, we estimate that an additional 46 seropositive cases would have been identified. As such, 18% (98/542) of COVID-19 cases were missed by RT-PCR in the setting of symptomatic presentations to primary care. This is similar to a pooled analysis showing a false-negative rate for RT-PCR of 20% at three days post-symptom onset.[29] Viral load peaks around the time of symptom onset and remains high over the first symptomatic week (also see Figure 4A).[30,31] Consistent with this, we found a slightly longer delay to swab

collection (due to delay in presentation to the platform) in RT-PCR false-negative patients than RT-PCR positive patients (Figure S4).

COVID-19 presents in a similar way to other respiratory viral illnesses. Indeed, in our cohort the most common symptoms of COVID-19 - such as cough, fatigue, headache, etc. - were reported with a similar frequency among patients testing negative. It is therefore important to have identified anosmia, ageusia, self-reported fever, myalgia, and anorexia as the symptoms with greatest value in the differential diagnosis of COVID-19 in primary care. This is consistent with systematic review evidence highlighting anosmia and ageusia as key diagnostic features of COVID19[32]. It is of note that 30% of jointly RT-PCR and serology negative patients reported these symptoms, indicating that although indicative of COVID-19, the specificity of these symptoms is not high enough to rule in the diagnosis alone. Sore throat and diarrhoea - both considered symptoms of COVID-19 in other settings –[33] were more frequently due to other possible aetiologies in this primary care context.

These results are robust for a number of reasons. Firstly, our sample is representative of the population of interest - i.e. consecutive patients with suspected COVID-19 in the community - instead of extrapolating from hospital cases. Symptom data were collected prospectively, eliminating recall or interviewer bias. Finally, we have a control group of patients who were negative for both RT-PCR and serology, minimizing misclassification due to false negative RT-PCR.

In our study, the proportion of patients with a positive SARS-CoV-2 RT-PCR requiring hospitalization was low (7%). Early reports from China were of 13.8% of cases being severe[34], but this value was lower when under ascertainment of cases was accounted for.[35,36] This is because our cohort reflects mild to moderate cases, as severely ill patients are likely to have attended hospital directly. As such, only 3% of patients we triaged to attend health services were ultimately hospitalized, possibly due to self-selection of patients presenting to our service. Supporting this, our overall case fatality ratio among RT-PCR positive patients was 0.7%. The rate of hospitalization was lower (0.5%) in those testing PCR-negative. These patients were admitted with a severe acute respiratory syndrome of an aetiology other than SARS-CoV-2. The 14-fold higher admission rate among PCR-positive cases highlights the importance of molecular testing for SARS-CoV-2 in patients presenting with features of respiratory viral illness to primary care.

For peer review only - http://bmjopen.bmj.com/site/about/guidelines.xhtml

As expected, the main determinant of Ct was the delay between symptom onset and swab collection, mostly due to the delay in reporting to the platform. After adjusting for this, as well as age and sex, we found that a self-reported fever and arthralgia were associated with lower Cts. The presence of these symptoms may identify patients with a higher viral load in the community. However, these results should be seen as purely exploratory, and the wide spread of Ct values around the regression line precludes a direct clinical application at present.

Our study has some limitations. Firstly, the small sample size preluded a multivariate analysis of factors associated with hospitalization or death. Next, serology was not performed on all RT-PCR negative patients due to on-going symptoms, loss to follow-up, or patient refusal. Of note, none of the RT-PCR-negative patients that were admitted to hospital underwent serology testing. This suggests that patients who were not tested with serology may have had a higher prevalence of COVID-19 than those that were tested. In addition, imperfect serology test performance (81% sensitivity)[26] will introduced false-negative results. Taken together, these biases may have underestimated the true seroprevalence among RT-PCR-negative cases, as well as the false-negative rate of RT-PCR. The latter calculation may also have been influenced by the inclusion of RT-PCR positive patients in the denominator, introducing an incorporation bias.[37] Furthermore, the association between symptoms and COVID-19 diagnosis was based on the comparison with doubly PCR and serology negative individuals. It is not clear how the exclusion of individuals that did not undergo serology testing would have influenced these associations. Finally, patients were not involved in the planning of the Corona platform or the research proposal.

A key strength to our study relates to the provision of primary healthcare in Brazil and its symbiosis with medical training nationwide. Primary health care - within the family health strategy (*Estratégia Saúde da Família*) - is centered around a healthcare unit with a multi-professional team that is responsible for all residents in the immediate catchment area [8]. São Caetano do Sul has enough GP units within the family health strategy that all residents have access to primary care. Medical students from the municipal university (USCS) are integrated into the primary healthcare teams and progressively trained from the first year of medical school. Our initiative took advantage of this existing system, with the addition of an online platform allowing remote clinical assessment and follow-up. The suspension of normal

For peer review only - http://bmjopen.bmj.com/site/about/guidelines.xhtml

clinical training at the medical school provided the workforce. The partnership with the University of São Paulo, which provided the laboratory diagnostics, created the unique opportunity to establish our prospective community cohort of suspected and confirmed COVID-19 cases. But we believe that this infrastructure could be implemented in other regions with less resources. Other respiratory disease such as influenza, measles, or tuberculosis may benefit from similar approach. However, further evaluation of the impact of the Corona Platform are required.

CONCLUSION

Systematic testing of all suspected COVID-19 cases was feasible within primary care services in a Brazilian municipality. Anosmia, agueusia, and fever provide the greatest diagnostic discrimination from other similar primary care presentations. Home-care is a valid approach for most of these patients with a low rate of hospitalization and death.
Our programme model – integrating multimedia technology, telehealth with universal access to primary care – may be successful in other contexts.

CONTRIBUTION STATEMENT

FEL, MCMC, SFC, MC, RB, and ECS conceived and designed the study. FEL, RMZG, and JCSB provided clinical oversight and supervision of medical students. FEL, MCMC, LFB, HD, OT, LC, and SRPS collected and curated the data. MCMC, TRTM, LSVB, and LCOS performed the laboratory analysis. LFB performed the formal statistical analysis with assistance from FEL, SRPS, NDEA, PM, ECS and OT. LFB, FEL, PM and ECS wrote the first draft, and all authors reviewed, contributed to and approved the final version.

CONFLICTS OF INTEREST STATEMENT

The authors have no conflicts of interests. FL, RG, and JB were involved in providing clinical care within the Corona São Caetano Platform.

FUNDING STATEMENT

For peer review only

The municipal health department of São Caetano do Sul (Secretaria Municipal de Saúde da Prefeitura de São Caetano do Sul) funded the establishment and implementation of the platform. We also acknowledge an award from FAPESP (2018/14389-0) and the UK Medical Research Council (MR/S0195/1) to the Brazil-UK Centre for Arbovirus Discovery, Diagnosis, Genomics and Epidemiology (CADDE).

DATA SHARING STATEMENT

Anonymized data are available at https://figshare.com/articles/dataset/Clinical_features_and_natural_history_of_the_first_2_073_suspected_COVID-19_cases_in_the_Corona_S_o_Caetano_primary_care_programme_a_prospective_cohort_study/13322474

REFERENCES

1. World Health Organization. (2020). Pneumonia of unknown cause – China. (https://www.who.int/csr/don/05-january-2020-pneumonia-of-unkown-cause-china/en/, accessed 2020-06-18).
2. World Health Organization. Critical preparedness, readiness and response actions for COVID-19 (Interim Guidance) (https://www.who.int/publications-detail/critical-preparedness-readiness-and-response-actions-for-covid-19, accessed 15 June 2020).
3. Bedford, J. et al. COVID-19: towards controlling of a pandemic. The Lancet **395**, 1015–1018 (2020).
4. Hellewell, J. et al. Feasibility of controlling COVID-19 outbreaks by isolation of cases and contacts. Lancet Glob Health **8**, e488–e496 (2020).

For peer review only

5. Dunlop, C., Howe, A., Li, D. & Allen, L. N. The coronavirus outbreak: the central role of primary care in emergency preparedness and response. BJGP Open **4**, bjgpopen20X101041 (2020).

6. World Health Organization. Regional Office for the Western Pacific. (2020). Role of primary care in the COVID-19 response. (https://apps.who.int/iris/handle/10665/331921, accessed 15 June, 2020).

7. Redwood-Campbell, L. & Abrahams, J. Primary Health care and Disasters—The Current State of the Literature: What We Know, Gaps and Next Steps. Prehosp. Disaster med. **26**, 184–191 (2011).

8. Macinko, J. & Harris, M. J. Brazil's Family Health Strategy — Delivering Community-Based Primary Care in a Universal Health System. NEJM **372**, 2177–2181 (2015).

9. World Health Organization. Situation report 27 (2020). (https://www.who.int/emergencies/diseases/novel-coronavirus-2019/situation-reports, accessed 15 June 2020).

10. Brazilian Ministry of Health. (https://covid.saude.gov.br/).

11. Buss, L. F. et al. COVID-19 herd immunity in the Brazilian Amazon. http://medrxiv.org/lookup/doi/10.1101/2020.09.16.20194787 (2020) doi:10.1101/2020.09.16.20194787.

12. de Souza, W. M. et al. Epidemiological and clinical characteristics of the COVID-19 epidemic in Brazil. Nat Hum Behav **4**, 856–865 (2020).

For peer review only

13. Hallal, P. C. et al. SARS-CoV-2 antibody prevalence in Brazil: results from two successive nationwide serological household surveys. The Lancet Global Health S2214109X20303879 (2020) doi:10.1016/S2214-109X(20)30387-9.
14. Docherty, A. B. et al. Features of 20 133 UK patients in hospital with covid-19 using the ISARIC WHO Clinical Characterisation Protocol: prospective observational cohort study. BMJ m1985 (2020) doi:10.1136/bmj.m1985.
15. Richardson, S. et al. Presenting Characteristics, Comorbidities, and Outcomes Among 5700 Patients Hospitalized With COVID-19 in the New York City Area. JAMA **323**, 2052 (2020).
16. Zhou, F. et al. Clinical course and risk factors for mortality of adult inpatients with COVID-19 in Wuhan, China: a retrospective cohort study. The Lancet **395**, 1054–1062 (2020).
17. Rauh, A. L. & Linder, J. A. Covid-19 care before, during, and beyond the hospital. BMJ m2035 (2020) doi:10.1136/bmj.m2035.
18. Lapostolle, F. et al. Clinical features of 1487 COVID-19 patients with outpatient management in the Greater Paris: the COVID-call study. Intern Emerg Med **15**, 813–817 (2020).
19. Mizrahi, B. et al. Longitudinal symptom dynamics of COVID-19 infection in primary care. http://medrxiv.org/lookup/doi/10.1101/2020.07.13.20151795 (2020) doi:10.1101/2020.07.13.20151795.
20. Williamson, E. J. et al. Factors associated with COVID-19-related death using OpenSAFELY. Nature **584**, 430–436 (2020).

For peer review only

For peer review only - http://bmjopen.bmj.com/site/about/guidelines.xhtml

21. Instituto Brasileiro de Geografia e Estatística (IBGE). (2020). São Caetano do Sul / Panorama. (https://cidades.ibge.gov.br/brasil/sp/sao-caetano-do-sul/panorama).

22. Human Development Atlas Brazil. (2020). São Caetano do Sul (http://atlasbrasil.org.br/2013/pt/perfil_m/sao-caetano-do-sul_sp).

23. Brazilian COVID-19 case definition and notification criteria. Brazilian Ministry of Health (https://coronavirus.saude.gov.br/definicao-de-caso-e-notificacao).

24. Akmatov, M. K., Gatzemeier, A., Schughart, K. & Pessler, F. Equivalence of Self- and Staff-Collected Nasal Swabs for the Detection of Viral Respiratory Pathogens. PLoS ONE **7**, e48508 (2012).

25. Walsh, K. A. et al. SARS-CoV-2 detection, viral load and infectivity over the course of an infection. Journal of Infection **81**, 357–371 (2020).

26. Whitman, J. D. et al. Test performance evaluation of SARS-CoV-2 serological assays. medRxiv 2020.04.25.20074856 (2020) doi:10.1101/2020.04.25.20074856.

27. R Core Team. R: A language and environment for statistical computing. R Foundation for Statistical Computing, Vienna, Austria. URL https://www.R-project.org/. (2019).

28. Baqui, P., Bica, I., Marra, V., Ercole, A. & van der Schaar, M. Ethnic and regional variations in hospital mortality from COVID-19 in Brazil: a cross-sectional observational study. The Lancet Global Health **8**, e1018–e1026 (2020).

29. Kucirka, L. M., Lauer, S. A., Laeyendecker, O., Boon, D. & Lessler, J. Variation in False-Negative Rate of Reverse Transcriptase Polymerase Chain Reaction–

For peer review only - http://bmjopen.bmj.com/site/about/guidelines.xhtml

Based SARS-CoV-2 Tests by Time Since Exposure. Ann Int Med M20-1495 (2020) doi:10.7326/M20-1495.

30. To, K. K.-W. et al. Temporal profiles of viral load in posterior oropharyngeal saliva samples and serum antibody responses during infection by SARS-CoV-2: an observational cohort study. Lancet Inf Dis **20**, 565–574 (2020).
31. He, X. et al. Temporal dynamics in viral shedding and transmissibility of COVID-19. Nat Med **26**, 672–675 (2020).
32. Carpenter, C. R., Mudd, P. A., West, C. P., Wilber, E. & Wilber, S. T. Diagnosing COVID-19 in the Emergency Department: A Scoping Review of Clinical Examinations, Laboratory Tests, Imaging Accuracy, and Biases. Acad Emerg Med **27**, 653–670 (2020).
33. Vetter, P. et al. Clinical features of covid-19. BMJ m1470 (2020) doi:10.1136/bmj.m1470.
34. T. Team, Vital Surveillances: The Epidemiological Characteristics of an Outbreak of 2019 Novel Coronavirus Diseases (COVID-19)-China. China CDC Weekly **2**, 113–122 (2020).
35. Salje, H. et al. Estimating the burden of SARS-CoV-2 in France. Science eabc3517 (2020) doi:10.1126/science.abc3517.
36. Verity, R. et al. Estimates of the severity of coronavirus disease 2019: a model-based analysis. Lancet Infect Dis **20**, 669–677 (2020).
37. Worster, A. & Carpenter, C. Incorporation bias in studies of diagnostic tests: how to avoid being biased about bias. CJEM **10**, 174–175 (2008).

For peer review only

For peer review only

For peer review only - http://bmjopen.bmj.com/site/about/guidelines.xhtml

TABLE LEGENDS

Table 1 Demographic and clinical characteristics of 1,048 suspected COVID-19 cases undergoing diagnostic testing in the Corona São Caetano program. * Security, emergency services, supermarket, public transport, and pharmacy workers. IQR: interquartile range; HCW: health care workers, COPD: chronic obstructive pulmonary disease. Missing data – educational level 2; essential occupation 2; body mass index 4; cardiovascular disease 28; diabetes 31 mellitus; chronic resp. disease 65; chronic kidney disease 27; COPD 28. P-values calculated by Chi-squared, Fisher exact, or Wilcoxon rank sum.

Table 2 Characteristics of RT-PCR positive patients stratified by hospitalization status. Missing data – body mass index 2; cardiovascular disease 12; diabetes mellitus 12; chronic respiratory disease 29; COPD 11; chronic kidney disease 12; COPD - chronic obstructive pulmonary disease; IQR - interquartile range.

FIGURE LEGENDS

Figure 1 Patient flowchart for the Corona São Caetano platform between 13th April and 13th May 2020. In the upper section (white background) the numbers correspond to individual presentations to the system; among suspected cases 2,073 suspected cases, 60 had two presentations and one had three. In the lower section (grey background) numbers correspond to individual patients making up the final analytic groups.

Figure 2 Panel A presents prevalence (point) and exact binomial 95% confidence intervals (vertical lines) of symptoms at presentation among patients with suspected COVID-19 according to RT-PCR result and serostatus (A). Panels B and C present the prevalence of presenting symptoms among patients with COVID-19 (RT-PCR and serology positive) stratified by age (B) and sex (C).

Figure 3 Odds ratios (black dot) and 95% confidence intervals (lines) for testing positive for COVID-19 (RT-PCR positive or serology positive) associated with the presence of each presenting symptom. Horizontal axis is on log scale. Point estimates of odds ratios are shown inline with their corresponding symptom.

For peer review only - http://bmjopen.bmj.com/site/about/guidelines.xhtml

Figure 4 Relationship between mean RT-PCR cycle threshold (Ct) and day of illness course when the nasopharyngeal swab was collected (A), patient age (B), patient sex (C), and different symptoms at presentation. Panels A and B show the best fit linear regression lines, panels C and D are violin plots (rotated kernel density plots showing the full distribution of data) of the Ct values with median (black dot) and interquartile range (black line).

For peer review only

Table 1

	**RT-PCR +ve (G1)** N = 444 n (%) or median (IQR)	**RT-PCR -ve Sero +ve (G2)** N=52 n (%) or median (IQR)	**RT-PCR -ve Sero -ve (G3)** N = 552 n (%) or median (IQR)	**p-value** G1 versus G2	**p-value** G1 versus G3
**Sex**					
Male	200 (45·0)	23 (44·2)	185 (33·5)		
Female	244 (55·0)	29 (55·8)	367 (66·5)	1·0	<0·001
**Age groups (years)**					
10 to 19	29 (6·5)	1 (1·9)	25 (4·5)		
20 to 39	197 (44·4)	17 (32·7)	236 (42·8)		
40 to 59	158 (35·6)	28 (53·8)	218 (39·5)		
60+	60 (13·5)	6 (11·5)	73 (13·2)	0·07	0·40
**Educational level**					
Up to primary education	75 (16·9)	7 (13·5)	56 (10·2)		
High school	214 (48·3)	19 (36·5)	194 (35·2)		
University	154 (34·8)	26 (50·0)	301 (54·6)	0·10	<0·001
**Essential Occupation**					
Non-HCW essential job *	137 (30·9)	12 (23·1)	148 (26·9)		
Carers	10 (2·3)	0 (0·0)	8 (1·5)		
HCW	32 (7·2)	5 (9·6)	73 (13·2)		
No	264 (59·6)	35 (67·3)	322 (58·4)	0·45	0·01
**Body mass index (kg/m²)**					
<25	151 (34·2)	22 (42·3)	211 (38·4)		
25-29	182 (41·2)	17 (32·7)	187 (34·0)		
30-35	79 (17·9)	9 (17·3)	112 (20·4)		
35+	30 (6·8)	4 (7·7)	40 (7·3)	0·62	0·14
**Comorbidities**					
Cardiovascular disease	88 (20·4)	9 (17·6)	129 (24·0)	0·89	0·40
Diabetes mellitus	48 (11·1)	4 (7·8)	39 (7·3)	0·86	0·12
Any chronic resp. disease	37 (8·9)	9 (18·0)	79 (15·3)	0·13	0·01
COPD	24 (5·5)	5 (9·8)	54 (10·1)	0·47	0·03
Chronic kidney disease	1 (<1)	0 (0·0)	3 (1·0)	1·0	0·83
**Time from symptom onset to swab collection (days)**, median (IQR)	5·0 (4·0-7·0)	6·0 (4·0-8·3)	6·0 (4·0-9·0)	0·06	<0·001

For peer review only

Table 2

	Hospitalized<br>n=30<br>n (%) or median (IQR)	Not hospitalized<br>n=414<br>n (%) or median (IQR)	p-value
**Age (years)**			
10 to 19	1 (3)	28 (97)	
20 to 39	6 (3)	191 (97)	
40 to 59	14 (9)	144 (91)	
60+	9 (15)	51 (85)	0·006
**Sex**			
Female	16 (7)	228 (93)	
Male	14 (7)	186 (93)	0·852
**Comorbidities**			
Cardiovascular disease	11 (13)	77 (87)	0·001
Diabetes mellitus	8 (17)	40 (83)	0·007
Any chronic resp. disease	2 (5)	35 (95)	1·0
COPD	1 (5)	23 (95)	1·0
Chronic kidney disease	1 (100)	0 (0)	0·06
**Body mass index (Kg/m²)**			
<25	4 (3)	147 (97)	
25-29	8 (4)	174 (96)	
30-35	12 (15)	67 (85)	
35+	6 (20)	24 (80)	<0·001
**Time to presentation (days)**	3 (3 to 4)	4 (3 to 5)	0·072

For peer review only

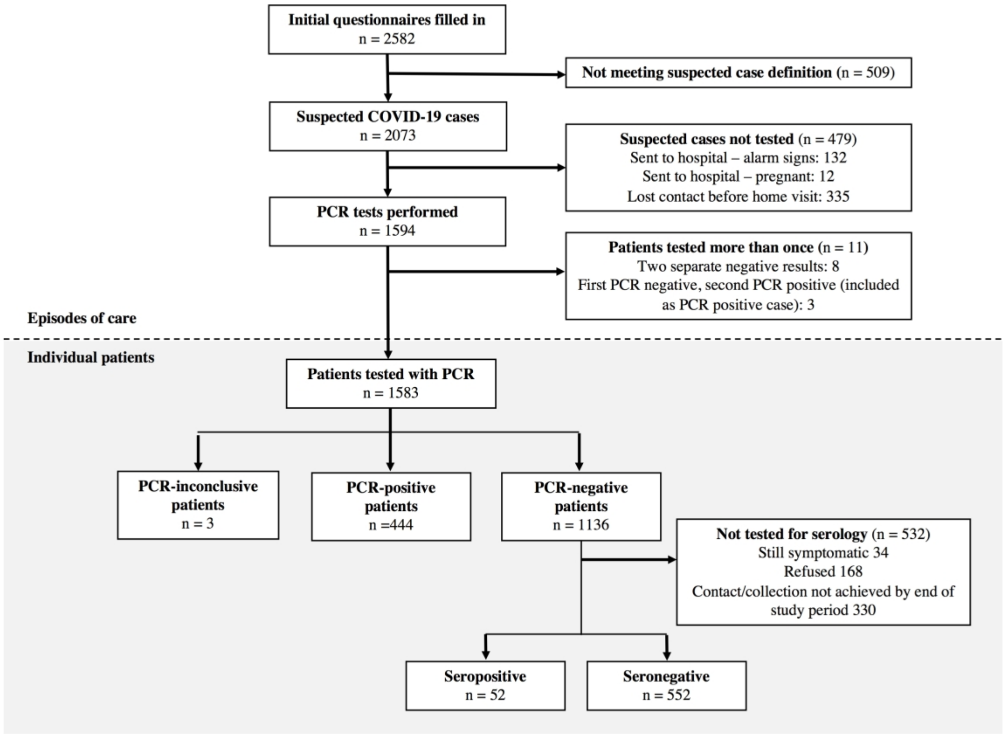

Figure 1

246x181mm (300 x 300 DPI)

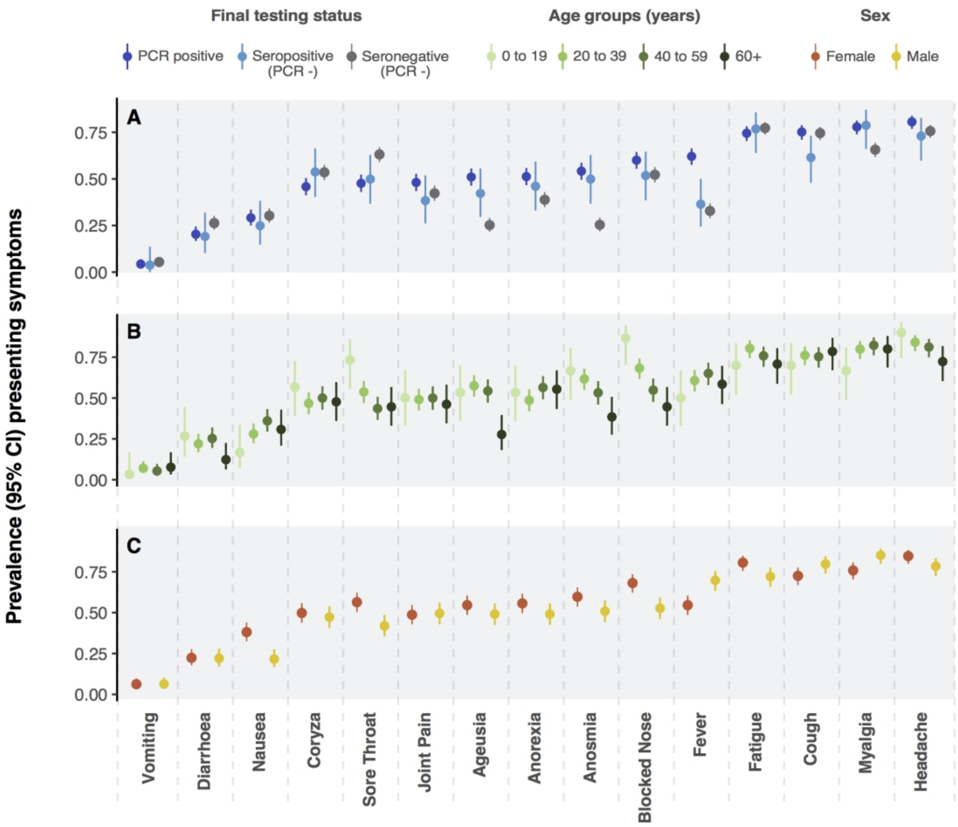

Figure 2

187x158mm (300 x 300 DPI)

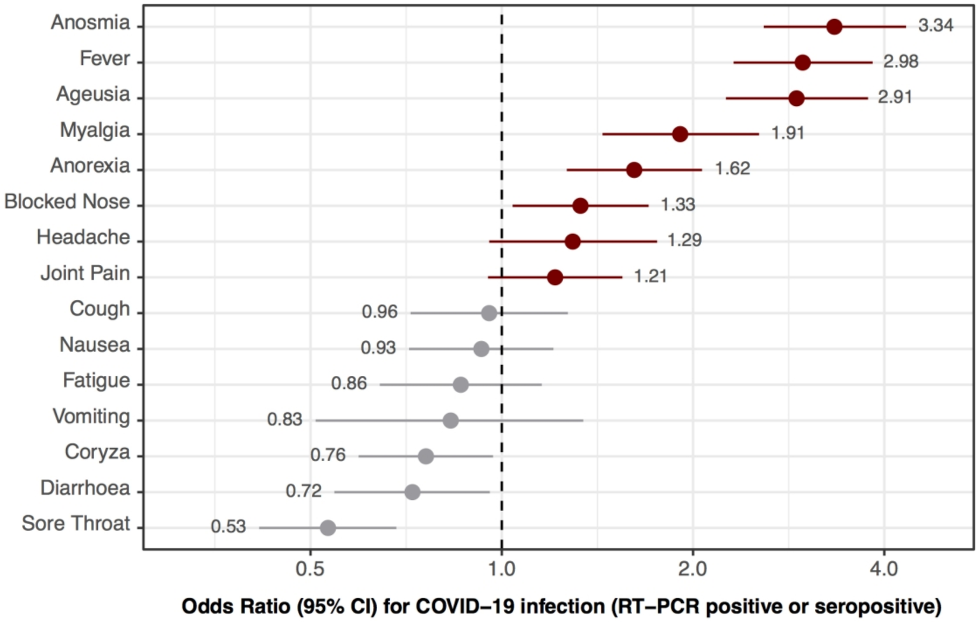

Figure 3

154x100mm (300 x 300 DPI)

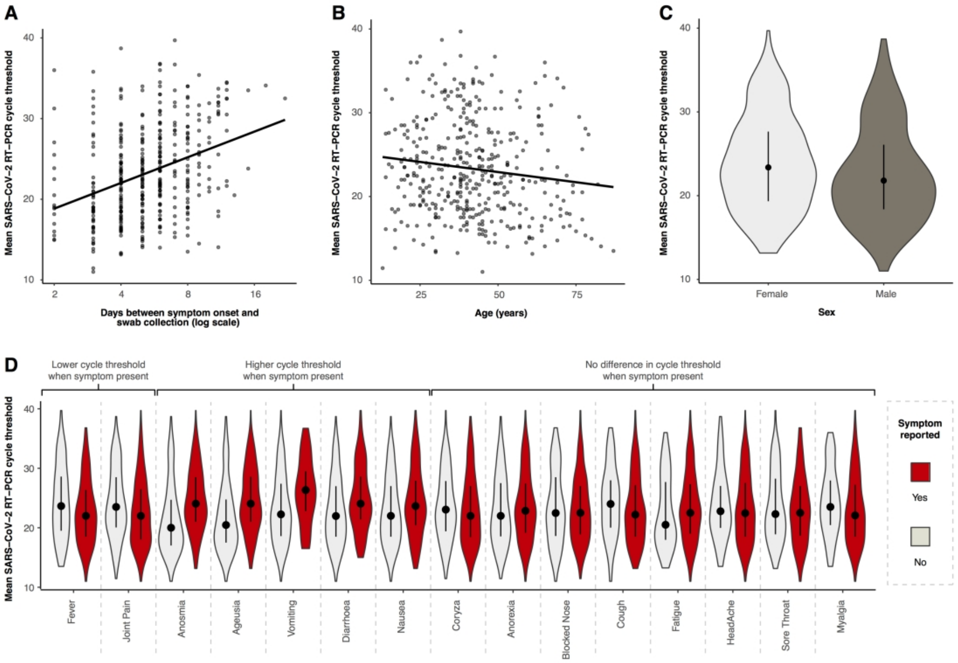

Figure 4

327x222mm (300 x 300 DPI)

Supplemental material

Contents:

1. **Initial risk assessment**
2. **Screen shots of multimedia platform and the initial questionnaire**
3. **Supplemental Table S1**
4. **Supplemental figs S1-S5**

Initial risk assessment

Patients meeting the definition of a suspected case were called by a medical student (under supervision) to complete a risk assessment. All patients were asked a set of standardized questions:

- Do you feel short of breath?
- Are you breathing quickly or finding it difficult to breath?
- If yes, can you count your respiratory rate over one minute? (respiratory rate >20 breaths/minute was considered tachypnoea)
- Has your fever worsened over the last 3 days or have you had a new fever after 2 days being fever-free?
- Have you felt confused or lethargic?

If the patient answered "yes" to any of these questions they were advised to attend a specialist health service. Among the 132 patients that were triaged to hospital, 76 (58% of 132) had shortness of breath, 76 (58% of 132) reported rapid breathing, 33 (25% of 132) persistent fever and 22 (17% of 132) altered mental status.

For peer review only

Screen shots showing examples of the initial questionnaire completed

1) Welcome page

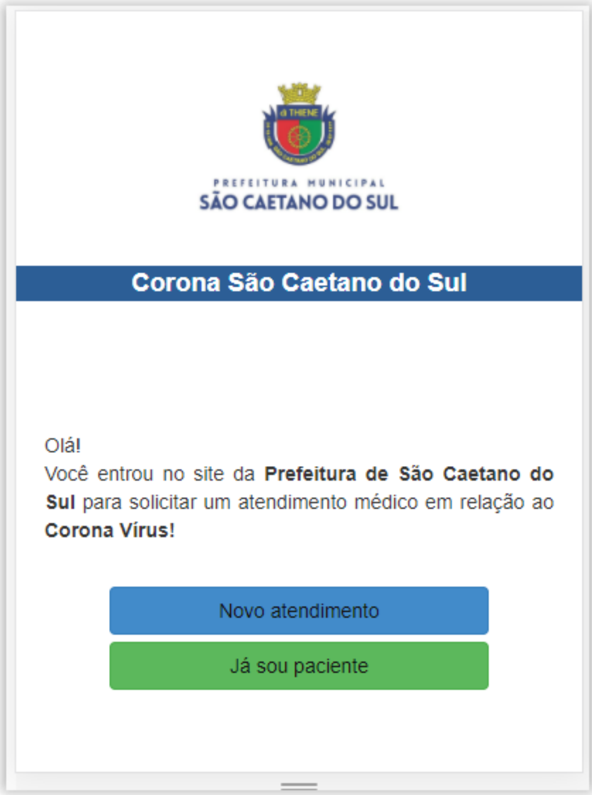

2) Zipcode confirmation

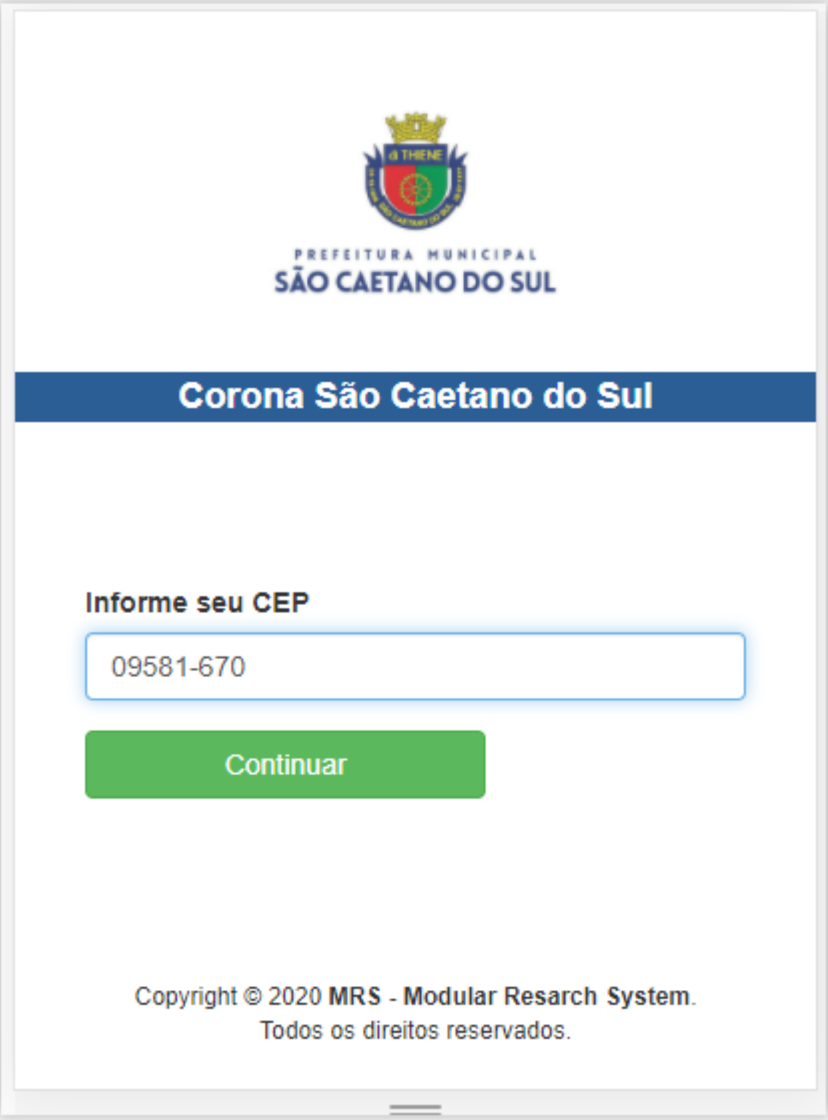

3) Patient basic information

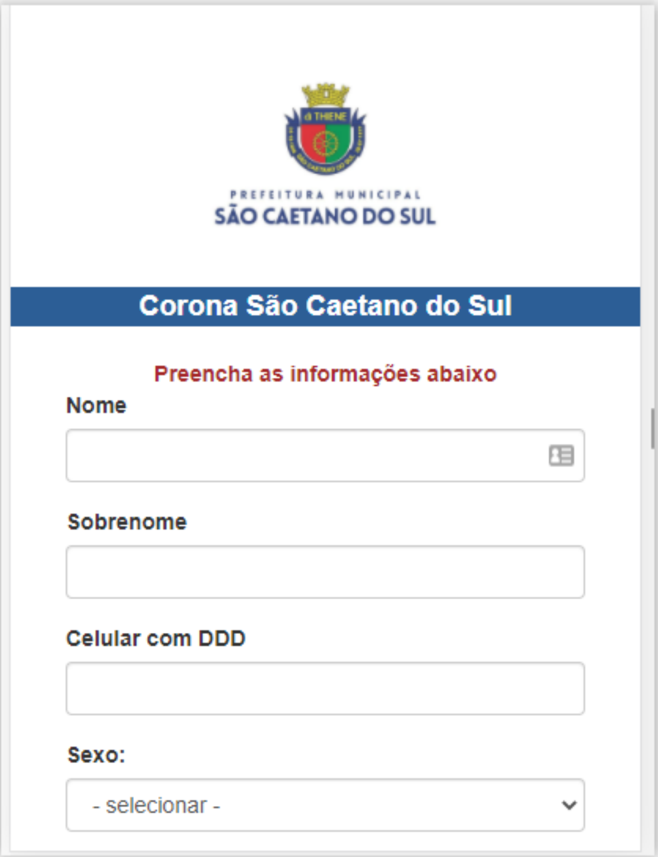

4) Access code confirmation

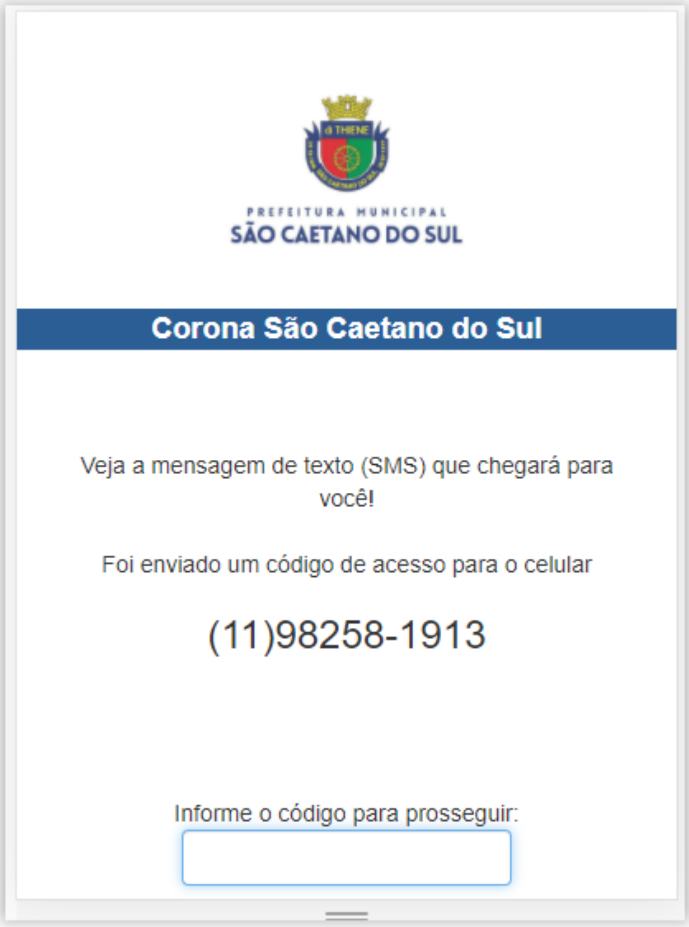

ew only

5) Questionnaire

Perguntas obrigatórias, favor responder:

Dados demográficos

Se do sexo feminino, está grávida?

- Não
- Sim
- Não se aplica

A partir do dia 1º de Março você atuou em alguma destas áreas:

- Não
- Profissional da saúde
- Áreas essenciais (segurança, bombeiro, farmácia, supermercado, transporte público)
- Cuidador (a)

Dados clínicos

Teve febre?

- Não
- Sim

Se sim, você mediu a febre?

- Não
- Sim

Se sim, qual foi a temperatura mais alta?

Você tem tosse?

- Não
- Sim

6) Orientation page

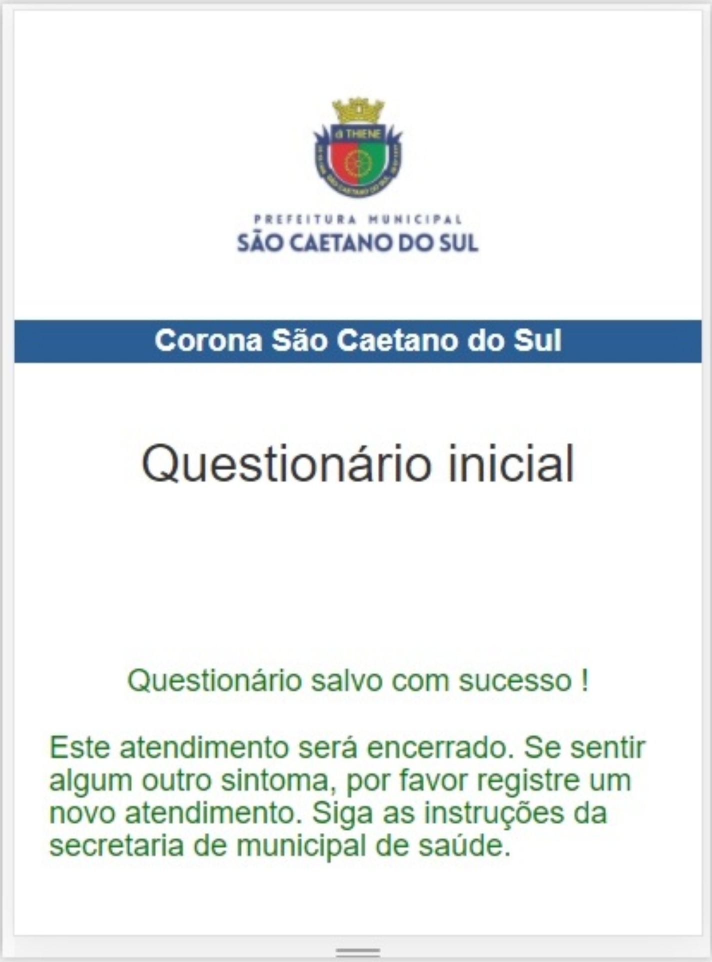

Table S1 Univariable and adjusted associations between RT-PCR cycle thresholds and patient characteristics

	**Unadjusted analysis**		**Adjusted analysis ***	
	**Beta** (difference in means)	**95% Confidence interval**	**Beta** (difference in means)	**95% Confidence interval**
**Age** (years)	**-0.05**	**-0.09 to -0.01**	**-0.06**	**-0.09 to -0.03**
**Male sex**	**-1.36**	**-2.49 to -0.23**	-1.05	-2.09 to <0.001
**Days from symptom onset to swab collection** (days, $\log_2$)	**3.28**	**2.33 to 4.03**	**3.27**	**0.42 to 7.70**
**PCR platform** (ALTONA as reference)	**-1.19**	**-2.37 to -0.02**	**-1.53**	**-2.6 to -0.45**
**Symptoms at presentation**				
Fever	**-1.78**	**-2.96 to -0.59**	**-1.11**	**-2.11 to -0.001**
Myalgia	-1.31	-2.75 to 0.12	-0.78	-2.11 to 0.53
Arthralgia	**-1.64**	**-2.77 to -0.52**	**-1.24**	**-2.18 to -0.10**
Anosmia	**3.15**	**2.04 to 4.25**	**2.21**	**1.0 to 3.29**
Agusia	**2.99**	**1.89 to 4.09**	**1.96**	**0.88 to 3.0**
Diarrhea	**2.19**	**0.84 to 3.53**	**1.36**	**0.12 to 2.61**
Nausea	**1.50**	**0.28 to 2.72**	1.09	-0.04 to 2.24
Vomiting	**2.99**	**0.52 to 5.46**	2.02	-0.28 to 4.33
Anorexia	0.56	-0.57 to 1.70	0.47	-0.58 to 1.51
Headache	-0.58	-2.12 to 0.97	-0.81	-2.25 to 0.63
Fatigue	0.84	-0.50 to 2.18	0.34	-0.91 to 1.59
Coryza	-0.78	-1.92 to 0.34	-0.68	-1.72 to 0.34
Blocked nose	-0.36	-1.53 to 0.81	**-1.48**	**-2.59 to -0.37**
Cough	-1.33	-2.70 to 0.03	**-1.60**	**-2.86 to -0.33**
Sore throat	-0.49	-1.62 to 0.64	-0.45	-1.52 to 0.61

* All variables adjusted for age (continuous in years), sex (female as reference group), PCR platform (ALTONA platform as the reference group) and time between symptom onset and swab collection (log base 2). Analysis was performed within a linear regression framework. Positive beta coefficients indicate higher cycle thresholds (lower viral load) associated with that variable, whereas negative beta coefficients indicate lower cycle thresholds when the variable is present. Results in bold reached statistical significance.

For peer review only

Supplemental figures

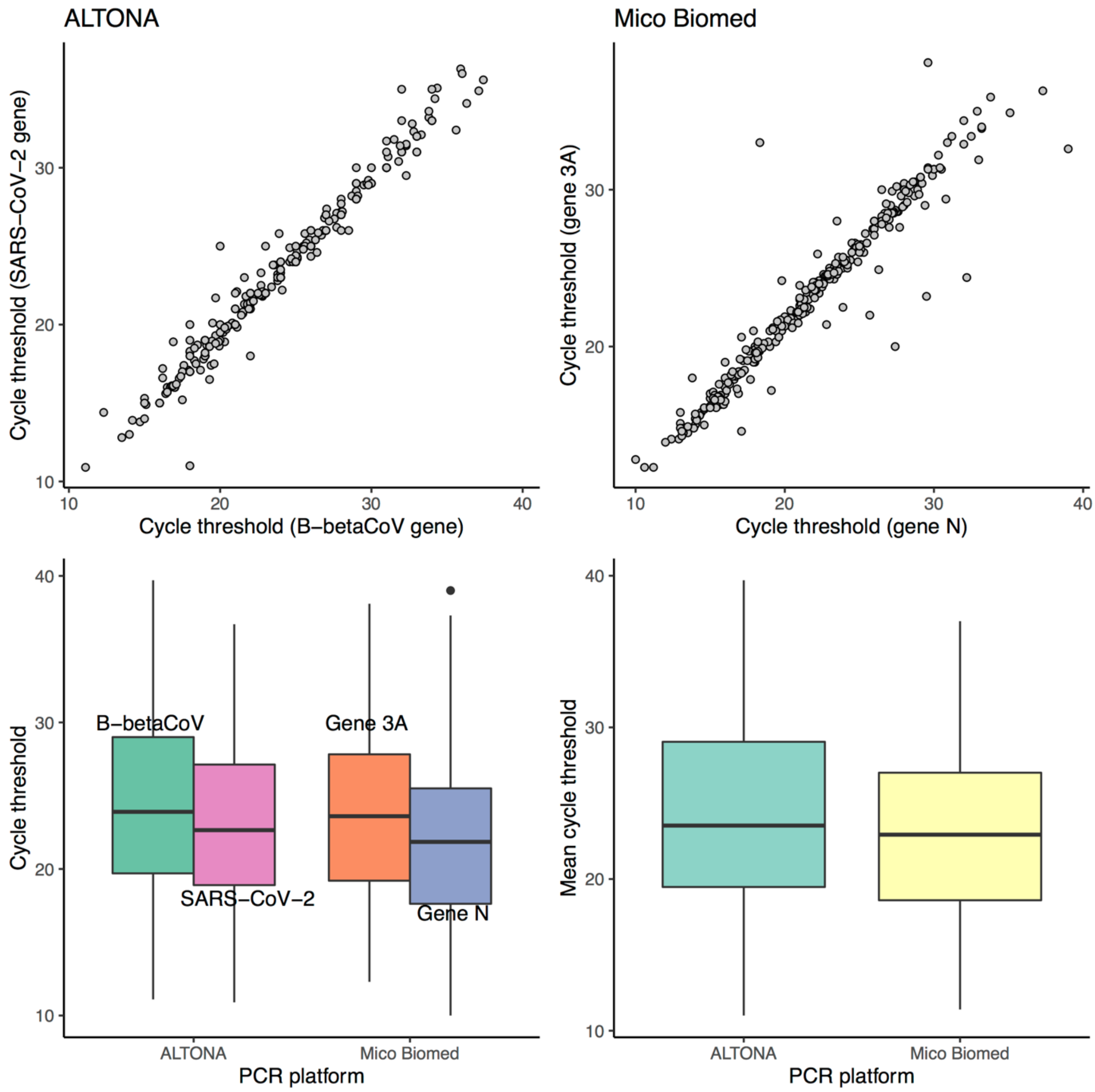

Figure S1 Comparison of cycle thresholds across PCR platforms and genes amplified. Upper two panels show the concordance between cycle thresholds for the two separate genes amplified by the ALTONA (left) and Mico Biomed (right) kits. Lower left panel – distribution of cycle thresholds by gene amplified and RT-PCR platform used. Lower right-hand panel – distribution of the mean cycle threshold (mean of cycle thresholds for separate genes) between different RT-PCR platforms.

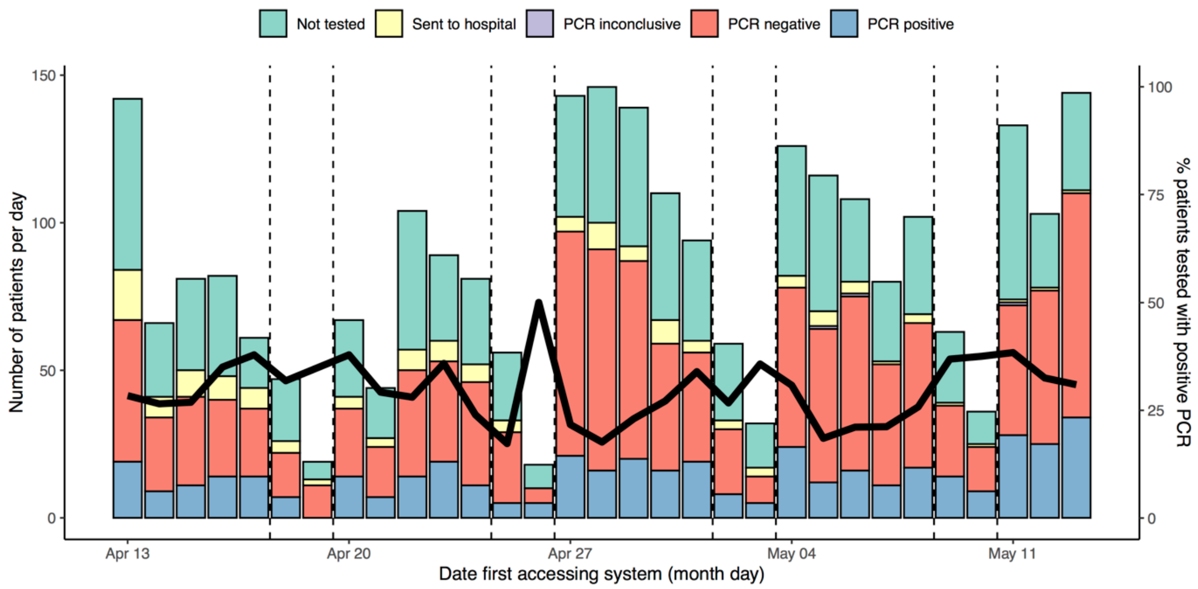

Figure S2 Time series of presentations to the Corona São Caetano platform. Dashed vertical lines denote the weekends with a reduced number of presentations. Thick black line corresponds to the right-hand y-axis: proportion of RT-PCRs performed with positive result.

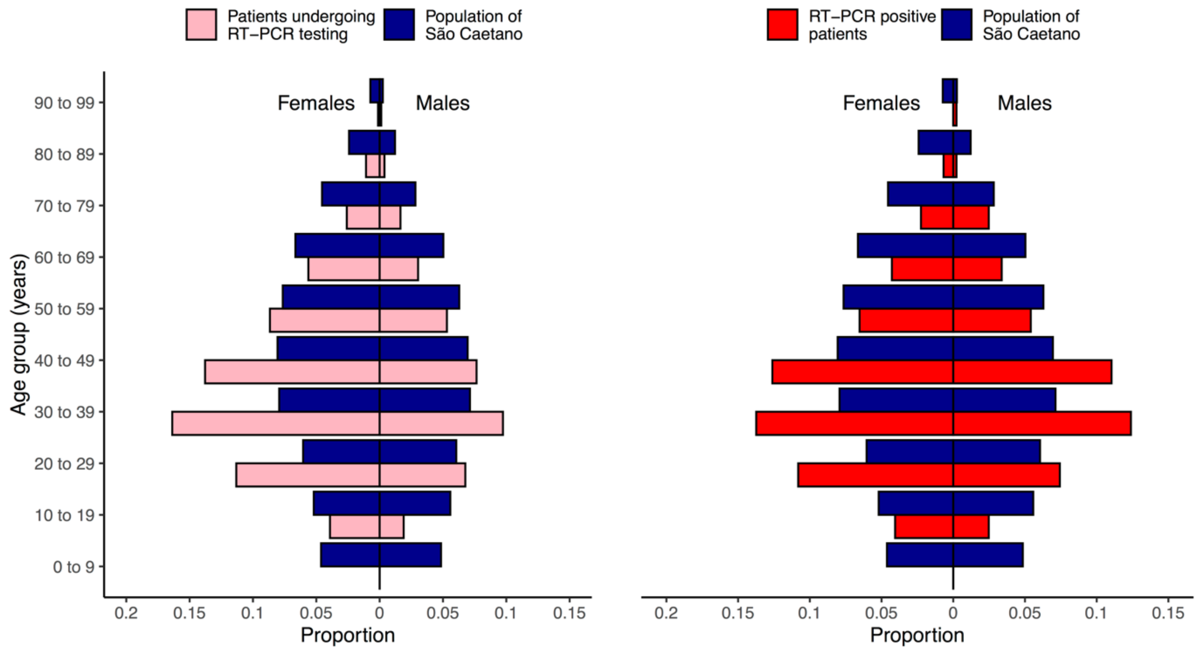

Figure S3 Age-sex distribution the city of São Caetano do Sul compared with that of patients accessing the Corona São Caetano system and being tested with RT-PCR (left-hand panel) and those testing positive for SARS-CoV-2 (right-hand panel).

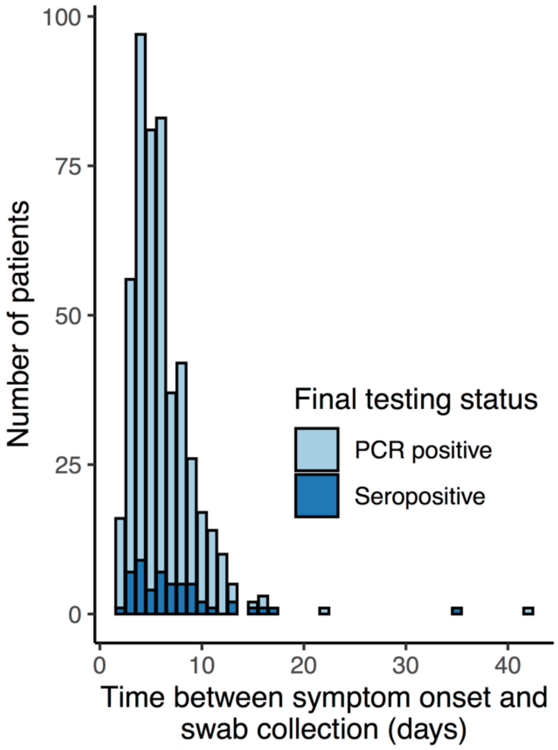

Figure S4 Histogram of delay between symptom onset and swab collection among patients with COVID-19.

For peer review only

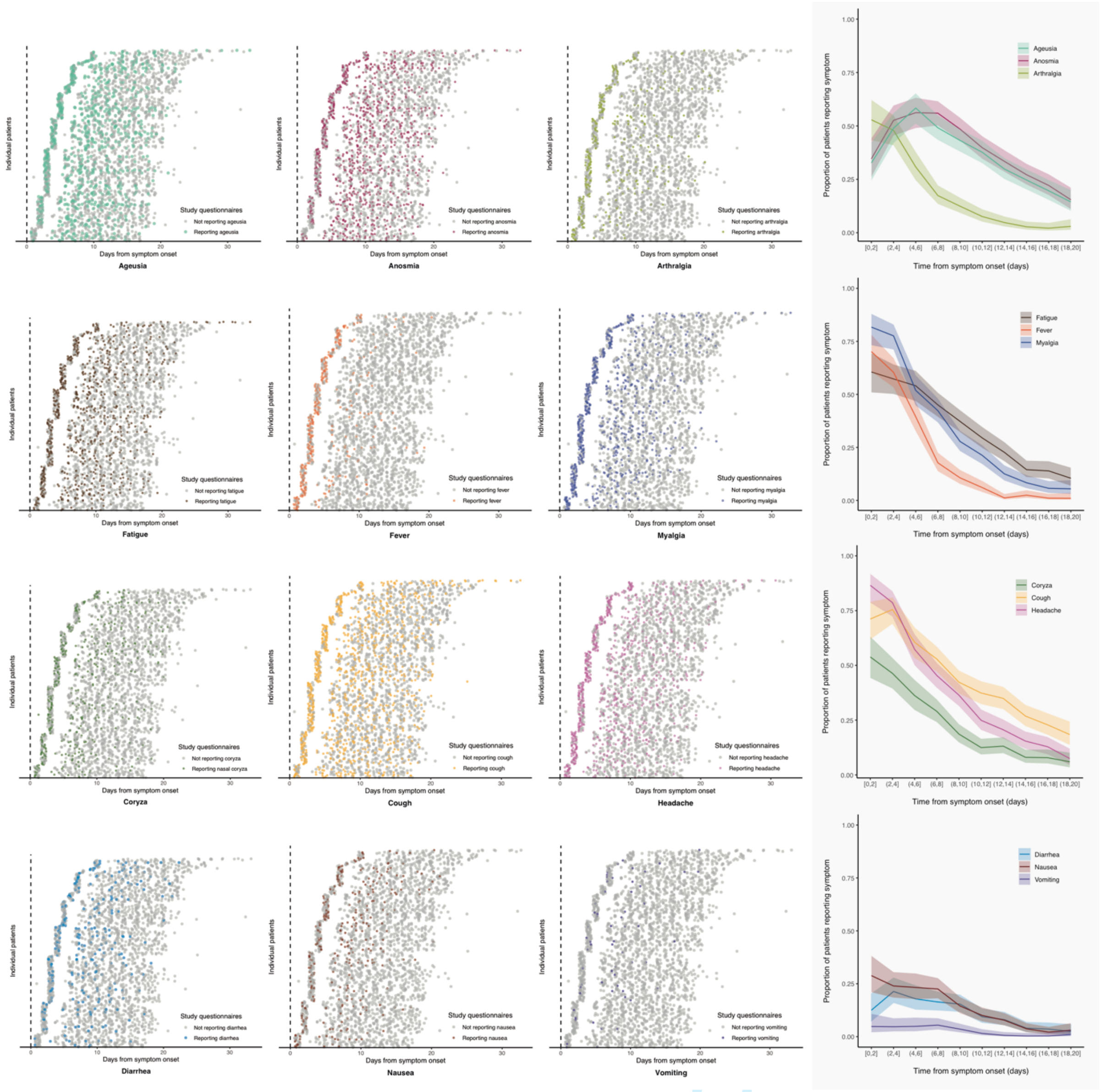

Figure S5 Left hand figures show symptoms at each follow-up questionnaire among patients testing RT-PCR positive and undergoing follow-up. Individual patients are stacked on the y-axis ordered according to the delay from symptom onset to presentation. Each point represents the response to a questionnaire and its position on the horizontal axis the time after symptom onset that the questionnaire was filled in. Grey points are questionnaires where the patient denied the presence of a given symptom. The coloured points correspond to questionnaires in which the patient reported a given symptom. The right-hand figures results from grouping the horizontal axis time into two-day windows and calculating the proportion of completed questionnaires in which each symptom was reported. The denominators for the horizontal axis groups (number of questionnaires completed within a given time window from symptom onset) are 104 at [0-2] days, 192 at (2-4], 185 at (4-6], 293 at (6-8], 338 at (8-10], 329 at (10-12], 335 at (12-14], 324 at (14-16], 280 at (16-18] and 201 at (18-20].

STROBE Statement—Checklist of items that should be included in reports of ***cohort studies***

	Item No	Recommendation	Page No
**Title and abstract**	1	(*a*) Indicate the study's design with a commonly used term in the title or the abstract<br>(*b*) Provide in the abstract an informative and balanced summary of what was done and what was found	1 and 2
**Introduction**			
Background/rationale	2	Explain the scientific background and rationale for the investigation being reported	4
Objectives	3	State specific objectives, including any prespecified hypotheses	4
**Methods**			
Study design	4	Present key elements of study design early in the paper	5 to 7
Setting	5	Describe the setting, locations, and relevant dates, including periods of recruitment, exposure, follow-up, and data collection	5 to 6
Participants	6	(*a*) Give the eligibility criteria, and the sources and methods of selection of participants. Describe methods of follow-up<br>(*b*) For matched studies, give matching criteria and number of exposed and unexposed	A – 5<br>B - NA
Variables	7	Clearly define all outcomes, exposures, predictors, potential confounders, and effect modifiers. Give diagnostic criteria, if applicable	7 to 8
Data sources/ measurement	8*	For each variable of interest, give sources of data and details of methods of assessment (measurement). Describe comparability of assessment methods if there is more than one group	5 to 7
Bias	9	Describe any efforts to address potential sources of bias	7 to 8
Study size	10	Explain how the study size was arrived at	8
Quantitative variables	11	Explain how quantitative variables were handled in the analyses. If applicable, describe which groupings were chosen and why	
Statistical methods	12	(*a*) Describe all statistical methods, including those used to control for confounding<br>(*b*) Describe any methods used to examine subgroups and interactions<br>(*c*) Explain how missing data were addressed<br>(*d*) If applicable, explain how loss to follow-up was addressed<br>(*e*) Describe any sensitivity analyses	A – 7 to 8<br>B – NA<br>C – 8<br>D – NA<br>E – NA
**Results**			
Participants	13*	(a) Report numbers of individuals at each stage of study—eg numbers potentially eligible, examined for eligibility, confirmed eligible, included in the study, completing follow-up, and analysed<br>(b) Give reasons for non-participation at each stage<br>(c) Consider use of a flow diagram	Figure 1 and page 9
Descriptive data	14*	(a) Give characteristics of study participants (eg demographic, clinical, social) and information on exposures and potential confounders<br>(b) Indicate number of participants with missing data for each variable of interest	A – table 1 and pages 9to10<br>B - Table 1 and 2 legends

For peer review only

		(c) Summarise follow-up time (eg, average and total amount)	C – page 9
Outcome data	15*	Report numbers of outcome events or summary measures over time	11
Main results	16	(*a*) Give unadjusted estimates and, if applicable, confounder-adjusted estimates and their precision (eg, 95% confidence interval). Make clear which confounders were adjusted for and why they were included<br>(*b*) Report category boundaries when continuous variables were categorized<br>(*c*) If relevant, consider translating estimates of relative risk into absolute risk for a meaningful time period	Page 7 and results section
Other analyses	17	Report other analyses done—eg analyses of subgroups and interactions, and sensitivity analyses	NA
**Discussion**			
Key results	18	Summarise key results with reference to study objectives	12
Limitations	19	Discuss limitations of the study, taking into account sources of potential bias or imprecision. Discuss both direction and magnitude of any potential bias	13
Interpretation	20	Give a cautious overall interpretation of results considering objectives, limitations, multiplicity of analyses, results from similar studies, and other relevant evidence	13
Generalisability	21	Discuss the generalisability (external validity) of the study results	13
**Other information**			
Funding	22	Give the source of funding and the role of the funders for the present study and, if applicable, for the original study on which the present article is based	14

*Give information separately for exposed and unexposed groups.

Note: An Explanation and Elaboration article discusses each checklist item and gives methodological background and published examples of transparent reporting. The STROBE checklist is best used in conjunction with this article (freely available on the Web sites of PLoS Medicine at http://www.plosmedicine.org/, Annals of Internal Medicine at http://www.annals.org/, and Epidemiology at http://www.epidem.com/). Information on the STROBE Initiative is available at http://www.strobe-statement.org.

For peer review only

For peer review only - http://bmjopen.bmj.com/site/about/guidelines.xhtml
